# Gerromorpha (Hemiptera: Heteroptera) from the Metropolitan Region of Santarém, Brazil, including three new species of *Microvelia* Westwood, 1834 (Veliidae: Microveliinae)

**DOI:** 10.3897/BDJ.9.e68567

**Published:** 2021-09-01

**Authors:** Suzane E. dos Santos, Juliana M. S. Rodrigues, Sheyla R. M. Couceiro, Felipe F. F. Moreira

**Affiliations:** 1 Laboratório de Ecologia e Taxonomia de Invertebrados Aquáticos, Universidade Federal do Oeste do Pará, Santarém, Brazil Laboratório de Ecologia e Taxonomia de Invertebrados Aquáticos, Universidade Federal do Oeste do Pará Santarém Brazil; 2 Laboratório de Biodiversidade Entomológica, Instituto Oswaldo Cruz, Fundação Oswaldo Cruz, Rio de Janeiro, Brazil Laboratório de Biodiversidade Entomológica, Instituto Oswaldo Cruz, Fundação Oswaldo Cruz Rio de Janeiro Brazil

**Keywords:** Amazon, aquatic ecosystems, aquatic insects, distribution, diversity, semi-aquatic bugs, systematics, taxonomy

## Abstract

**Background:**

Gerromorpha (Hemiptera: Heteroptera) comprises more than 2100 species of semiaquatic bugs, most of which have the ability to walk on the surface of the water. So far, 238 species have been recorded from Brazil, but several portions of the country remain poorly explored. The Metropolitan Region of Santarém (MRS), Pará State, Brazil, lacks faunistic and taxonomic studies concerning this group and the local fauna is under threat due to human actions.

**New information:**

Aiming to fill gaps concerning the diversity and distribution of Gerromorpha in the Amazon, a survey of the semi-aquatic bugs from the MRS is presented. Collections were made in 33 aquatic ecosystems in the different phytophysiognomies within this area from July 2019 to October 2020. As a result, a checklist with 44 species recorded from the three municipalities of the MRS is presented. Furthermore, three new species of the genus *Microvelia* Westwood, 1834 (*M.belterrensis* **sp. nov.**, *M.hamadae* **sp. nov.** and *M.sousorum* **sp. nov.**) are described, two species are recorded for the first time from Brazil (*Microveliaaschnakiranae* Makhan, 2014 and *Rhagoveliagraziae* Galindo-Malagón, Morales & Moreira, 2021), two from Pará State (*Microvelialongipes* Uhler, 1894 and *Paraveliadilatata* Polhemus & Polhemus, 1984) and 15 from the MRS (*Brachymetralata* Shaw, 1933, *B.shawi* Hungerford & Matsuda, 1957, *Tachygerrisadamsoni* (Drake, 1942), *Microveliapulchella* Westwood, 1834, *Rhagoveliabrunae* Magalhães & Moreira, 2016, *R.evidis* Bacon, 1948, *R.jubata* Bacon, 1948, *Calliveliaconata* (Hungerford, 1929), *Oioveliacunucunumana* Drake & Maldonado-Capriles, 1952, *Paraveliabullialata* Polhemus & Polhemus, 1984, *Striduliveliaalia* (Drake, 1957), *S.stridulata* (Hungerford, 1929), *S.strigosa* (Hungerford, 1929), *S.tersa* (Drake & Harris, 1941) and *S.transversa* (Hungerford, 1929)).

## Introduction

Gerromorpha (Hemiptera: Heteroptera) is an infra-order of predatory, semi-aquatic bugs, most of which live on the surface of the water or amongst floating plants (Nieser and Melo 1997). Contrary to the Nepomorpha, or truly aquatic bugs, their antennae are long and plainly visible dorsally, inserted in front of the eyes. The body is 1.0 to 36.0 mm long and usually velvety, covered with a double layer of micro- and macrotrichia (Andersen 1982). The legs are usually narrow, with apical or pre-apical pretarsal claws (Ribeiro et al. 2019).

More than 2100 species have been described in Gerromorpha, distributed in eight families: Gerridae, Hebridae, Hermatobatidae, Hydrometridae, Macroveliidae, Mesoveliidae, Paraphrynoveliidae and Veliidae. In the Neotropical Region, the infra-order is represented by more than 45 genera and 500 species (Polhemus and Polhemus 2008), of which 238 species have been recorded from Brazil (Moreira 2021a, Moreira 2021b, Moreira 2021c, Moreira 2021d, Moreira 2021e).

Despite the number of recorded species, semi-aquatic bugs are still poorly known in several portions of the country, whereas the south-eastern region and the central Amazon are better explored (Moreira et al. 2011). Aiming to fill gaps concerning the diversity and distribution of Gerromorpha in the Amazon (Nessimian et al. 2019), we present a survey of the semi-aquatic bugs from the Metropolitan Region of Santarém (MRS), Pará State, Brazil. The area lacks faunistic and taxonomic studies and the local fauna is under threat due to human actions (Couceiro and Hamada 2011).

## Materials and methods

**Figure 1.**
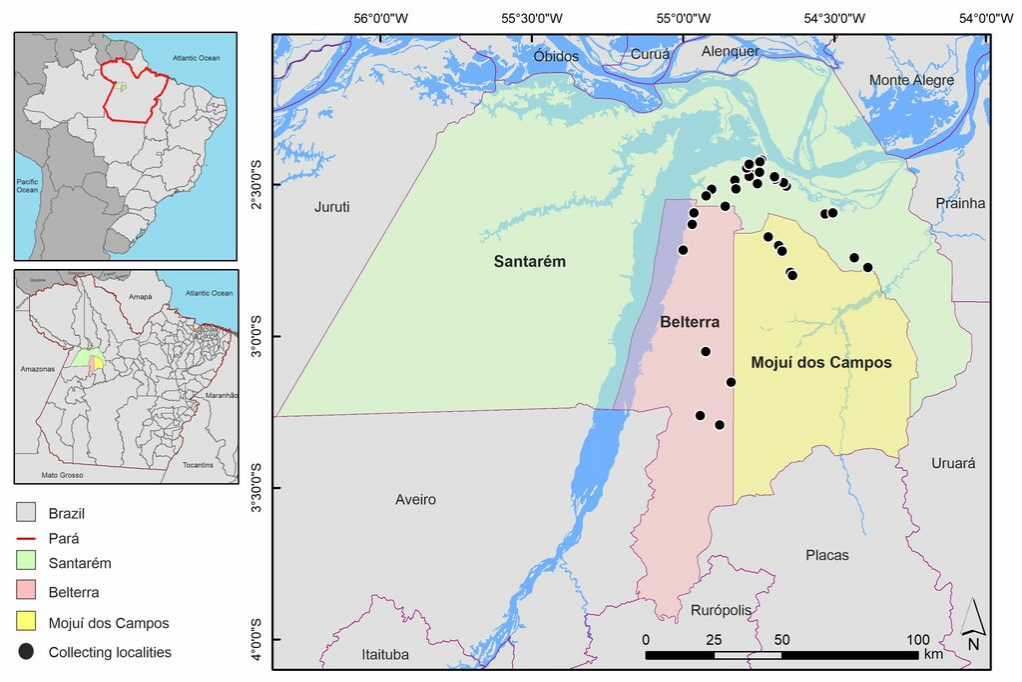
Collecting localities of Gerromorpha in the MRS, Pará State, northern Brazil.

**Figure 2.**
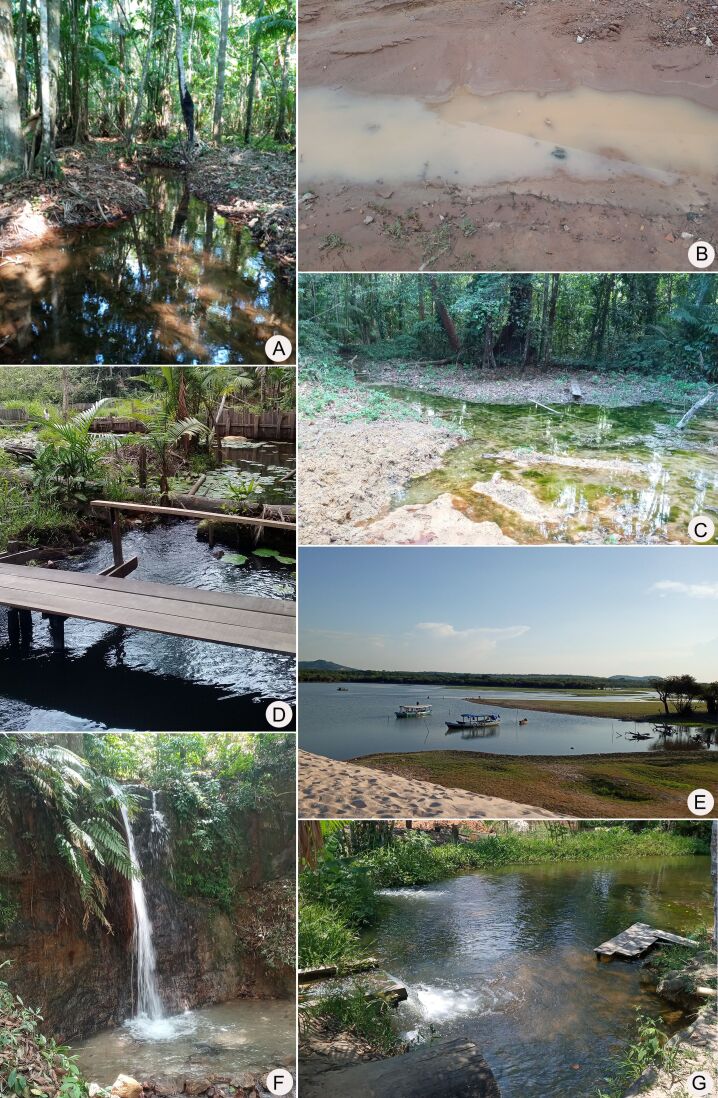
Photographs of some collecting localities. **A.** Igarapé Cajutuba II, Santarém; **B.** Temporary puddle, Santarém; **C.** Nascente, Igarapé Água Fria, Mojuí dos Campos; **D.** Igarapé Guaraná, Santarém; **E.** Lago do Juá, Santarém; **F.** Cachoeira Rocha Negra, Santarém; **G.** Igarapé Jatobá, Santarém.

**Figure 3.**
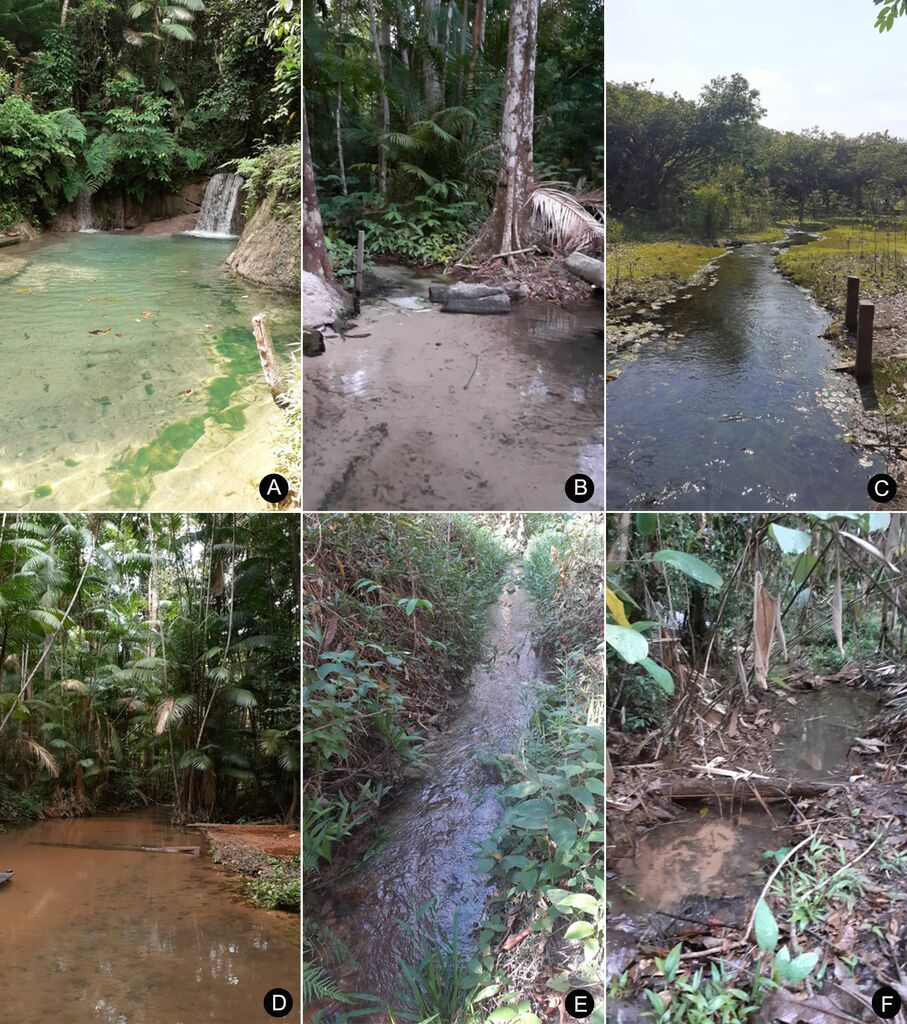
Photographs of some collecting localities. **A.** Cachoeira da Cavada, Santarém; **B.** Igarapé do Ailton, Belterra; **C.** Igarapé Aramanaí, Belterra; **D.** Igarapé Coronel Batista, Belterra; **E.** Igarapé do Rai, Santarém; **F.** Igarapé, BR-163, Km-115, Belterra.

We collected in the MRS, western Pará State, Brazil (Fig. 1). The region includes the Municipalities of Belterra, Mojuí dos Campos and Santarém, with an area of 27,285.426 km^2^ (Prefeitura Municipal de Santarém 2017). The climate type is Am, tropical, with average annual rainfall of 2000 mm (Rodrigues 2001). We sampled in 33 aquatic ecosystems in the different phytophysiognomies within the area, including natural lakes, temporary pools, waterfalls, rivers, streams and springs (Figs 2, 3; Table 1). We used a GARMIN eTrex 30 GPS receiver to georeference the sampling stations. We obtained specimens with aquatic D-nets and sieves on the surface of the water bodies, including riffles, pools, marginal vegetation and foam. We then fixed and preserved the material in 70% ethanol and labelled it with locality, date and collector data.

To identify the specimens, we used information available in Bacon (1956), Spangler (1990), Nieser (1994), Nieser and Melo (1997), Polhemus (1997), Moreira and Barbosa (2013), Makhan (2014), Rodrigues et al. (2014a), Rodrigues et al. (2014b), Floriano et al. (2016b), Floriano et al. (2016a), Floriano et al. (2017), Magalhães et al. (2016), Cordeiro (2017), Moreira et al. (2018), Galindo-Malagón et al. (2021). Type-specimens are deposited in the Coleção Entomológica do Instituto Oswaldo Cruz, Fundação Oswaldo Cruz, Rio de Janeiro, Brazil (CEIOC). Other specimens are deposited in the Laboratório de Ecologia e Taxonomia de Invertebrados, Universidade Federal do Oeste do Pará, Santarém, Brazil (LETIA). Distribution data presented for each species are based on Moreira (2021a), Moreira (2021b), Moreira (2021c), Moreira (2021d), Moreira (2021e).

We made descriptions and photographs, based on dry specimens. All measurements are given in millimetres and abbreviated as follows: body length (BL), head length (HL), head width (HW), minimum head width between the eyes (INT), length of antennomeres I–IV (ANT I, ANT II, ANT III, ANT IV), width of eye (EYE), pronotum length on mid-line (PL), pronotum width (PW), length of femur (FEM), length of tibia (TIB) and length of tarsomeres I–II (TAR I, TAR II).

## Taxon treatments

### Brachymetra lata

Shaw, 1933

76BF0FD1-5B6E-5F80-ADF1-6A33CA6C025B

Brachymetra lata – see Shaw (1933): 227, pl. XX, fig. 4.

#### Materials

- **Type status:**Other material. **Occurrence:** recordedBy: S.E. Santos; sex: 3 apterous ?, 5 apterous ?; **Location:** country: Brazil; stateProvince: Pará; municipality: Belterra; locality: Floresta Nacional do Tapajós; verbatimLatitude: 03°03'02.6"S; verbatimLongitude: 54°55'30.1"W; **Event:** verbatimEventDate: 20.I.2020; habitat: igarapé; **Record Level:** type: PhysicalObject; institutionCode: LETIA; basisOfRecord: PreservedSpecimen
- **Type status:**Other material. **Occurrence:** recordedBy: S.E. Santos; sex: 10 apterous ?, 7 apterous ?; **Location:** country: Brazil; stateProvince: Pará; municipality: Belterra; locality: BR-163, km-115; verbatimLatitude: 03°17'34.8"S; verbatimLongitude: 54°52'45.6"W; **Event:** verbatimEventDate: 23.XI.2019; habitat: igarapé; **Record Level:** type: PhysicalObject; institutionCode: LETIA; basisOfRecord: PreservedSpecimen
- **Type status:**Other material. **Occurrence:** recordedBy: S.E. Santos; sex: 2 apterous ?; **Location:** country: Brazil; stateProvince: Pará; municipality: Belterra; locality: Igarapé do Ailton; verbatimLatitude: 02°35'36.7''S; verbatimLongitude: 54°57'48.4''W; **Event:** verbatimEventDate: 06.XI.2019; **Record Level:** type: PhysicalObject; institutionCode: LETIA; basisOfRecord: PreservedSpecimen
- **Type status:**Other material. **Occurrence:** recordedBy: S.E. Santos; sex: 2 apterous ?, 2 apterous ?; **Location:** country: Brazil; stateProvince: Pará; municipality: Belterra; locality: Igarapé Coronel Batista; verbatimLatitude: 02°37'50.6"S; verbatimLongitude: 54°58'12.4"W; **Event:** verbatimEventDate: 08.XI.2019; **Record Level:** type: PhysicalObject; institutionCode: LETIA; basisOfRecord: PreservedSpecimen
- **Type status:**Other material. **Occurrence:** recordedBy: S.E. Santos; sex: 8 apterous ?, 3 apterous ?; **Location:** country: Brazil; stateProvince: Pará; municipality: Mojuí dos Campos; locality: Igarapé do Manel; verbatimLatitude: 02°25'06.3"S; verbatimLongitude: 54°44'26.3"W; **Event:** verbatimEventDate: 24.II.2020; **Record Level:** type: PhysicalObject; institutionCode: LETIA; basisOfRecord: PreservedSpecimen
- **Type status:**Other material. **Occurrence:** recordedBy: S.E. Santos; sex: 1 apterous ?; **Location:** country: Brazil; stateProvince: Pará; municipality: Mojuí dos Campos; locality: Igarapé Antonio Leite; verbatimLatitude: 03°09'06.2"S; verbatimLongitude: 54°50'28.7"W; **Event:** verbatimEventDate: 18.X.2019; **Record Level:** type: PhysicalObject; institutionCode: LETIA; basisOfRecord: PreservedSpecimen
- **Type status:**Other material. **Occurrence:** recordedBy: S.E. Santos; sex: 1 apterous ?, 4 apterous ?; **Location:** country: Brazil; stateProvince: Pará; municipality: Mojuí dos Campos; locality: Igarapé Terra Preta; verbatimLatitude: 02°43'09.1"S; verbatimLongitude: 54°40'20.7"W; **Event:** verbatimEventDate: 10.II.2020; **Record Level:** type: PhysicalObject; institutionCode: LETIA; basisOfRecord: PreservedSpecimen
- **Type status:**Other material. **Occurrence:** recordedBy: S.E. Santos; sex: 1 apterous ?; **Location:** country: Brazil; stateProvince: Pará; municipality: Mojuí dos Campos; locality: Igarapé Terra Preta; verbatimLatitude: 02°43'09.1"S; verbatimLongitude: 54°40'20.7"W; **Event:** verbatimEventDate: 24.II.2020; **Record Level:** type: PhysicalObject; institutionCode: LETIA; basisOfRecord: PreservedSpecimen
- **Type status:**Other material. **Occurrence:** recordedBy: S.E. Santos; sex: 1 apterous ?; **Location:** country: Brazil; stateProvince: Pará; municipality: Mojuí dos Campos; locality: Igarapé Terra de Areia; verbatimLatitude: 02°47'58.7"S; verbatimLongitude: 54°38'15.6"W; **Event:** verbatimEventDate: 24.I.2020; **Record Level:** type: PhysicalObject; institutionCode: LETIA; basisOfRecord: PreservedSpecimen
- **Type status:**Other material. **Occurrence:** recordedBy: S.E. Santos; sex: 2 apterous ?; **Location:** country: Brazil; stateProvince: Pará; municipality: Mojuí dos Campos; locality: Igarapé Água Fria; verbatimLatitude: 02°47'19.7"S; verbatimLongitude: 54°38'40.9"W; **Event:** verbatimEventDate: 24.IX.2020; **Record Level:** type: PhysicalObject; institutionCode: LETIA; basisOfRecord: PreservedSpecimen
- **Type status:**Other material. **Occurrence:** recordedBy: S.E. Santos; sex: 1 apterous ?, 1 apterous ?; **Location:** country: Brazil; stateProvince: Pará; municipality: Santarém; locality: Igarapé Jatobá; verbatimLatitude: 02°34'17.9"S; verbatimLongitude: 54°51'36.8"W; **Event:** verbatimEventDate: 10.X.2020; **Record Level:** type: PhysicalObject; institutionCode: LETIA; basisOfRecord: PreservedSpecimen
- **Type status:**Other material. **Occurrence:** recordedBy: S.E. Santos; sex: 10 apterous ?, 8 apterous ?; **Location:** country: Brazil; stateProvince: Pará; municipality: Santarém; locality: Cachoeira da Rocha Negra; verbatimLatitude: 02°29'48.5"S; verbatimLongitude: 54°45'13.3"W; **Event:** verbatimEventDate: 25.IX.2020; **Record Level:** type: PhysicalObject; institutionCode: LETIA; basisOfRecord: PreservedSpecimen
- **Type status:**Other material. **Occurrence:** recordedBy: S.E. Santos; sex: 1 apterous ?, 1 apterous ?; **Location:** country: Brazil; stateProvince: Pará; municipality: Santarém; locality: Igarapé Cajutuba II; verbatimLatitude: 02°27'39.2"S; verbatimLongitude: 54°46'53.4"W; **Event:** verbatimEventDate: 10.X.2020; **Record Level:** type: PhysicalObject; institutionCode: LETIA; basisOfRecord: PreservedSpecimen
- **Type status:**Other material. **Occurrence:** recordedBy: S.E. Santos; sex: 1 apterous ?, 5 apterous ?; **Location:** country: Brazil; stateProvince: Pará; municipality: Santarém; locality: Igarapé Sonrizal; verbatimLatitude: 02°32'13.6''S; verbatimLongitude: 54°55'2.6''W; **Event:** verbatimEventDate: 09.VIII.2019; **Record Level:** type: PhysicalObject; institutionCode: LETIA; basisOfRecord: PreservedSpecimen
- **Type status:**Other material. **Occurrence:** recordedBy: S.E. Santos; sex: 1 macropterous ?; **Location:** country: Brazil; stateProvince: Pará; municipality: Santarém; locality: Cachoeira da Cavada; verbatimLatitude: 02°35'48.9"S; verbatimLongitude: 54°31'47.3"W; **Event:** verbatimEventDate: 21.X.2019; **Record Level:** type: PhysicalObject; institutionCode: LETIA; basisOfRecord: PreservedSpecimen
- **Type status:**Other material. **Occurrence:** recordedBy: S.E. Santos; sex: 1 apterous ?, 1 apterous ?; **Location:** country: Brazil; stateProvince: Pará; municipality: Santarém; locality: Igarapé das Bananeiras; verbatimLatitude: 02°30'52"S; verbatimLongitude: 54°54'20"W; **Event:** verbatimEventDate: 20.X.2019; **Record Level:** type: PhysicalObject; institutionCode: LETIA; basisOfRecord: PreservedSpecimen
- **Type status:**Other material. **Occurrence:** recordedBy: S.E. Santos; sex: 11 apterous ?, 18 apterous ?, 1 macropterous ?; **Location:** country: Brazil; stateProvince: Pará; municipality: Santarém; locality: Igarapé Diamantino; verbatimLatitude: 02°30'16.2''S; verbatimLongitude: 54°39'32.9''W; **Event:** verbatimEventDate: 06.IX.2019; **Record Level:** type: PhysicalObject; institutionCode: LETIA; basisOfRecord: PreservedSpecimen
- **Type status:**Other material. **Occurrence:** recordedBy: S.E. Santos; sex: 1 macropterous ?, 4 macropterous ?; **Location:** country: Brazil; stateProvince: Pará; municipality: Santarém; locality: Igarapé do Rai; verbatimLatitude: 02°35'35.3"S; verbatimLongitude: 54°30'18.1"W; **Event:** verbatimEventDate: 13.XI.2019; **Record Level:** type: PhysicalObject; institutionCode: LETIA; basisOfRecord: PreservedSpecimen

#### Distribution

Brazil (Amapá, Amazonas, Maranhão, Mato Grosso, Pará, Rondônia, Roraima), Colombia, Ecuador, French Guyana, Suriname, Venezuela (Moreira 2021a).

#### Notes

First records from the study area.

#### Photograph

Fig. 4a

### Brachymetra shawi

Hungerford & Matsuda, 1957

32C39CE2-FABF-53C0-B9B0-7561A92C6D99

**Figure 5.**
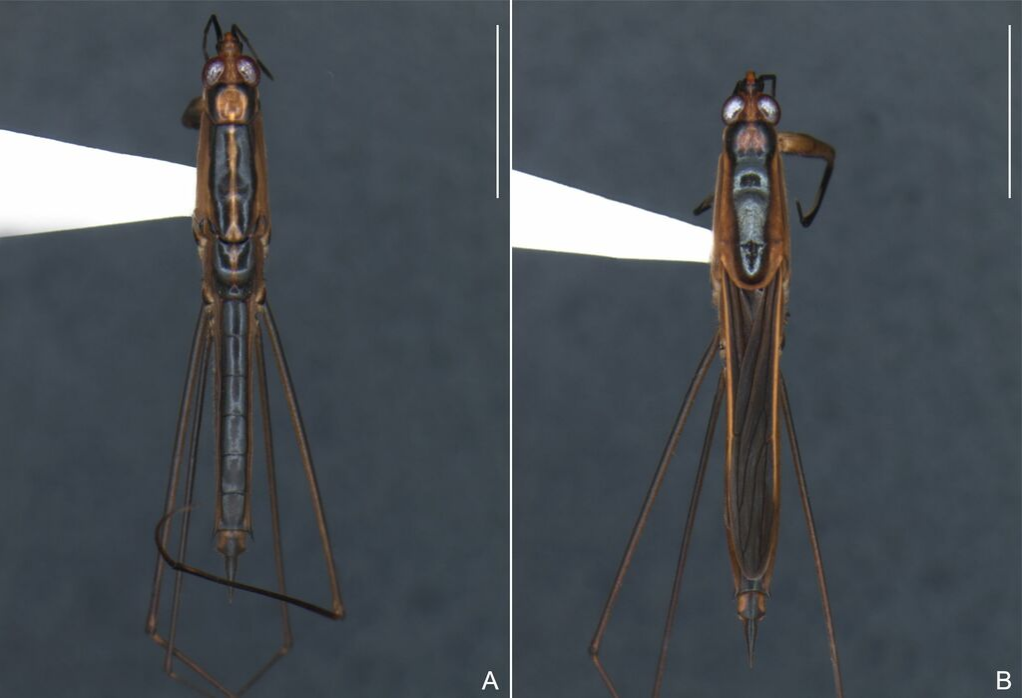
*Cylindrostethuspalmaris*, habitus, dorsal view. **A.** Apterous male; **B.** Macropterous male. Scale bars: 2 mm.

Brachymetra kleopatra – see Shaw (1933): 226, pl. XX, fig. 5 (misidentification).
Brachymetra shawi – see Hungerford and Matsuda (1957): 22, pl. I–II.

#### Materials

- **Type status:**Other material. **Occurrence:** recordedBy: S.E. Santos; sex: 3 apterous ?, 1 apterous ?, 5 macropterous ?; **Location:** country: Brazil; stateProvince: Pará; municipality: Santarém; locality: Igarapé da Débora; verbatimLatitude: 02°44'27.7"S; verbatimLongitude: 54°26'01.2"W; **Event:** verbatimEventDate: 21.X.2019; **Record Level:** type: PhysicalObject; institutionCode: LETIA; basisOfRecord: PreservedSpecimen
- **Type status:**Other material. **Occurrence:** recordedBy: S.E. Santos; sex: 5 macropterous ?; **Location:** country: Brazil; stateProvince: Pará; municipality: Santarém; locality: Cachoeira da Rocha Negra; verbatimLatitude: 02°29'48.5"S; verbatimLongitude: 54°45'13.3"W; **Event:** verbatimEventDate: 25.IX.2020; **Record Level:** type: PhysicalObject; institutionCode: LETIA; basisOfRecord: PreservedSpecimen
- **Type status:**Other material. **Occurrence:** recordedBy: S.E. Santos; sex: 1 macropterous ?; **Location:** country: Brazil; stateProvince: Pará; municipality: Santarém; locality: Caixa d'água; verbatimLatitude: 02°27'31.7"S; verbatimLongitude: 54°44'49.4"W; **Event:** verbatimEventDate: 10.IX.2020; **Record Level:** type: PhysicalObject; institutionCode: LETIA; basisOfRecord: PreservedSpecimen

#### Distribution

Bolivia, Brazil (Amazonas, Mato Grosso, Pará, Rondônia), Colombia, French Guiana, Guyana, Suriname, Trinidad and Tobago (Moreira 2021a).

#### Notes

First records from the study area.

#### Photographs

Fig. 4b, c

### Cylindrostethus drakei

Floriano, Paladini & Cavichioli, 2016

E51A247B-0C55-5B01-B5BB-FF82124ED396

Cylindrostethus linearis – see Drake and Harris (1934): 220, pl. XXV; Nieser (1970): 120, figs. 157–158; Moreira et al. (2011b): 274, figs. 8–9 (misidentification).
Cylindrostethus drakei – see Floriano et al. (2016a): 460, figs. 1, 7–9 and 13–15.

#### Distribution

Brazil (Amazonas, Pará, Rondônia), Peru (Moreira 2021a).

#### Notes

Previously recorded from Santarém (Nieser 1970; misidentified as *C.linearis*), but absent from our samples.

### Cylindrostethus palmaris

A002374A-2EC1-536D-826C-60C0CC23CD80

Cylindrostethus linearis – see Drake and Harris (1930): 238; Drake and Harris (1941a): 240 (partim; misidentification).
Cylindrostethus palmaris – see Drake and Harris (1934): 222.

#### Materials

- **Type status:**Other material. **Occurrence:** recordedBy: S.E. Santos; sex: 2 macropterous ?, 2 macropterous ?; **Location:** country: Brazil; stateProvince: Pará; municipality: Belterra; locality: BR-163, Km-115; verbatimLatitude: 03°17'34.8"S; verbatimLongitude: 54°52'45.6"W; **Event:** verbatimEventDate: 23.XI.2019; habitat: igarapé; **Record Level:** type: PhysicalObject; institutionCode: LETIA; basisOfRecord: PreservedSpecimen
- **Type status:**Other material. **Occurrence:** recordedBy: S.E. Santos; sex: 1 apterous ?, 1 apterous ?, 1 macropterous ?, 1 macropterous ?; **Location:** country: Brazil; stateProvince: Pará; municipality: Belterra; locality: Igarapé Coronel Batista; verbatimLatitude: 02°37'50.6"S; verbatimLongitude: 54°58'12.4"W; **Event:** verbatimEventDate: 08.XI.2019; **Record Level:** type: PhysicalObject; institutionCode: LETIA; basisOfRecord: PreservedSpecimen
- **Type status:**Other material. **Occurrence:** recordedBy: S.E. Santos; sex: 1 apterous ?, 2 apterous ?, 1 macropterous ?, 1 macropterous ?; **Location:** country: Brazil; stateProvince: Pará; municipality: Belterra; locality: Igarapé do Ailton; verbatimLatitude: 02°35'36.7"S; verbatimLongitude: 54°57'48.4"W; **Event:** verbatimEventDate: 06.XI.2019; **Record Level:** type: PhysicalObject; institutionCode: LETIA; basisOfRecord: PreservedSpecimen
- **Type status:**Other material. **Occurrence:** recordedBy: S.E. Santos; sex: 1 apterous ?, 2 apterous ?, 1 macropterous ?, 1 macropterous ?; **Location:** country: Brazil; stateProvince: Pará; municipality: Belterra; locality: Igarapé do Ailton; verbatimLatitude: 02°35'36.7"S; verbatimLongitude: 54°57'48.4"W; **Event:** verbatimEventDate: 06.XI.2019; **Record Level:** type: PhysicalObject; institutionCode: LETIA; basisOfRecord: PreservedSpecimen
- **Type status:**Other material. **Occurrence:** recordedBy: S.E. Santos; sex: 11 apterous ?, 7 apterous ?; **Location:** country: Brazil; stateProvince: Pará; municipality: Mojuí dos Campos; locality: Igarapé do Manel; verbatimLatitude: 02°25'06.3"S; verbatimLongitude: 54°44'26.3"W; **Event:** verbatimEventDate: 24.II.2020; **Record Level:** type: PhysicalObject; institutionCode: LETIA; basisOfRecord: PreservedSpecimen
- **Type status:**Other material. **Occurrence:** recordedBy: S.E. Santos; sex: 2 apterous ?, 1 apterous ?; **Location:** country: Brazil; stateProvince: Pará; municipality: Mojuí dos Campos; locality: Igarapé Terra Preta; verbatimLatitude: 02°43'09.1"S; verbatimLongitude: 54°40'20.7"W; **Event:** verbatimEventDate: 10.II.2020; **Record Level:** type: PhysicalObject; institutionCode: LETIA; basisOfRecord: PreservedSpecimen
- **Type status:**Other material. **Occurrence:** recordedBy: S.E. Santos; sex: 2 apterous ?; **Location:** country: Brazil; stateProvince: Pará; municipality: Mojuí dos Campos; locality: Igarapé Santa Júlia; verbatimLatitude: 02°40'19.7"S; verbatimLongitude: 54°43'06.9"W; **Event:** verbatimEventDate: 09.XII.2019; **Record Level:** type: PhysicalObject; institutionCode: LETIA; basisOfRecord: PreservedSpecimen
- **Type status:**Other material. **Occurrence:** recordedBy: S.E. Santos; sex: 1 macropterous ?, 1 macropterous ?; **Location:** country: Brazil; stateProvince: Pará; municipality: Mojuí dos Campos; locality: Igarapé Antonio Leite; verbatimLatitude: 03°09'06.2"S; verbatimLongitude: 54°50'28.7"W; **Event:** verbatimEventDate: 18.X.2019; **Record Level:** type: PhysicalObject; institutionCode: LETIA; basisOfRecord: PreservedSpecimen
- **Type status:**Other material. **Occurrence:** recordedBy: S.E. Santos; sex: 2 apterous ?; **Location:** country: Brazil; stateProvince: Pará; municipality: Mojuí dos Campos; locality: Igarapé Mojuí dos Caboclos; verbatimLatitude: 02°42'03.0"S; verbatimLongitude: 54°41'01.0"W; **Event:** verbatimEventDate: 21.I.2020; **Record Level:** type: PhysicalObject; institutionCode: LETIA; basisOfRecord: PreservedSpecimen
- **Type status:**Other material. **Occurrence:** recordedBy: S.E. Santos; sex: 22 apterous ?, 14 apterous ?, 1 macropterous ?; **Location:** country: Brazil; stateProvince: Pará; municipality: Santarém; locality: Igarapé Guaraná; verbatimLatitude: 02°46'25.9"S; verbatimLongitude: 54°23'20.6"W; **Event:** verbatimEventDate: 06.III.2020; **Record Level:** type: PhysicalObject; institutionCode: LETIA; basisOfRecord: PreservedSpecimen
- **Type status:**Other material. **Occurrence:** recordedBy: S.E. Santos; sex: 1 apterous ?; **Location:** country: Brazil; stateProvince: Pará; municipality: Santarém; locality: Igarapé São Braz; verbatimLatitude: 02°29'07.0"S; verbatimLongitude: 54°49'41.9"W; **Event:** verbatimEventDate: 26.VII.2019; **Record Level:** type: PhysicalObject; institutionCode: LETIA; basisOfRecord: PreservedSpecimen
- **Type status:**Other material. **Occurrence:** recordedBy: S.E. Santos; sex: 1 apterous ?, 1 apterous ?, 2 macropterous ?, 1 macropterous ?; **Location:** country: Brazil; stateProvince: Pará; municipality: Santarém; locality: Igarapé Diamantino; verbatimLatitude: 02°30'16.2"S; verbatimLongitude: 54°39'32.9"W; **Event:** verbatimEventDate: 06.IX.2019; **Record Level:** type: PhysicalObject; institutionCode: LETIA; basisOfRecord: PreservedSpecimen
- **Type status:**Other material. **Occurrence:** recordedBy: S.E. Santos; sex: 2 macropterous ?, 1 macropterous ?; **Location:** country: Brazil; stateProvince: Pará; municipality: Santarém; locality: Cachoeira da Rocha Negra; verbatimLatitude: 02°29'48.5"S; verbatimLongitude: 54°45'13.3"W; **Event:** verbatimEventDate: 25.IX.2020; **Record Level:** type: PhysicalObject; institutionCode: LETIA; basisOfRecord: PreservedSpecimen
- **Type status:**Other material. **Occurrence:** recordedBy: S.E. Santos; sex: 1 apterous ?, 1 apterous ?, 1 macropterous ?; **Location:** country: Brazil; stateProvince: Pará; municipality: Santarém; locality: Igarapé Jatobá; verbatimLatitude: 02°34'17.9"S; verbatimLongitude: 54°51'36.8"W; **Event:** verbatimEventDate: 10.X.2020; **Record Level:** type: PhysicalObject; institutionCode: LETIA; basisOfRecord: PreservedSpecimen

#### Distribution

Argentina, Bolivia, Brazil (Alagoas, Amapá, Amazonas, Bahia, Espírito Santo, Goiás, Maranhão, Mato Grosso, Mato Grosso do Sul, Minas Gerais, Pará, Rio de Janeiro, Rio Grande do Norte, Rondônia, Roraima, São Paulo, Sergipe), Colombia, Ecuador, French Guiana, Guyana, Peru, Suriname, Trinidad and Tobago, Venezuela (Moreira 2021a).

#### Notes

Previously recorded from Santarém (Kuitert 1942); first records from Belterra and Mojuí dos Campos.

#### Photographs

Fig. 5

### Limnogonus aduncus aduncus

Drake & Harris, 1933

C27DAD63-E8DB-5497-B1DD-584C9F0F1238

Limnogonus aduncus – see Drake and Harris (1933): 110.
Limnogonus aduncus aduncus – see Kuitert (1942): 130.
Limnogonus recurvus – see Mascarenhas (1979): 763, figs 1–3 (misidentification).
Tenagogonus spinulosus – see Poisson (1955): 68, figs 6, 7 (syn. by Andersen 1995: 115).

#### Materials

- **Type status:**Other material. **Occurrence:** recordedBy: S.E. Santos; sex: 3 macropterous ?, 1 macropterous ?; **Location:** country: Brazil; stateProvince: Pará; municipality: Santarém; locality: Cachoeira da Cavada; verbatimLatitude: 02°35'48.9"S; verbatimLongitude: 54°31'47.3"W; **Event:** verbatimEventDate: 13.XI.2019; **Record Level:** type: PhysicalObject; institutionCode: LETIA; basisOfRecord: PreservedSpecimen
- **Type status:**Other material. **Occurrence:** recordedBy: S.E. Santos; sex: 1 apterous ?, 1 apterous ?; **Location:** country: Brazil; stateProvince: Pará; municipality: Santarém; locality: Igarapé Jatobá; verbatimLatitude: 02°34'17.9"S; verbatimLongitude: 54°51'36.8"W; **Event:** verbatimEventDate: 10.X.2020; **Record Level:** type: PhysicalObject; institutionCode: LETIA; basisOfRecord: PreservedSpecimen
- **Type status:**Other material. **Occurrence:** recordedBy: S.E. Santos; sex: 5 apterous ?, 2 apterous ?; **Location:** country: Brazil; stateProvince: Pará; municipality: Santarém; locality: Igarapé do Rai; verbatimLatitude: 02°35'35.3"S; verbatimLongitude: 54°30'18.1"W; **Event:** verbatimEventDate: 13.XI.2019; **Record Level:** type: PhysicalObject; institutionCode: LETIA; basisOfRecord: PreservedSpecimen
- **Type status:**Other material. **Occurrence:** recordedBy: S.E. Santos; sex: 1 macropterous ?; **Location:** country: Brazil; stateProvince: Pará; municipality: Santarém; locality: Igarapé Diamantino; verbatimLatitude: 02°30'16.2"S; verbatimLongitude: 54°39'32.9"W; **Event:** verbatimEventDate: 06.IX.2019; **Record Level:** type: PhysicalObject; institutionCode: LETIA; basisOfRecord: PreservedSpecimen

#### Distribution

Argentina, Bolivia, Brazil (Alagoas, Amazonas, Bahia, Espírito Santo, Mato Grosso, Mato Grosso do Sul, Minas Gerais, Pará, Paraná, Pernambuco, Rio de Janeiro, Roraima, Santa Catarina, São Paulo, Sergipe), Colombia, Ecuador, Guyana, Panama, Paraguay, Peru, Suriname, Trinidad and Tobago, Venezuela (Moreira 2021a).

#### Notes

Previously recorded from Santarém (Nieser 1970).

#### Photograph

Fig. 6a

### Limnogonus recurvus

Drake & Harris, 1930

0945712F-B8D7-588D-9CEF-6BAAAB2CF910

Limnogonus recurvus – see Drake and Harris (1930): 236.

#### Materials

- **Type status:**Other material. **Occurrence:** recordedBy: S.E. Santos; sex: 1 apterous ?; **Location:** country: Brazil; stateProvince: Pará; municipality: Belterra; locality: Igarapé Jatuarana; verbatimLatitude: 03°15'44.7"S; verbatimLongitude: 54°56'37.5"W; **Event:** verbatimEventDate: 11.II.2020; **Record Level:** type: PhysicalObject; institutionCode: LETIA; basisOfRecord: PreservedSpecimen
- **Type status:**Other material. **Occurrence:** recordedBy: S.E. Santos; sex: 1 apterous ?, 1 apterous ?; **Location:** country: Brazil; stateProvince: Pará; municipality: Mojuí dos Campos; locality: Igarapé Terra Preta; verbatimLatitude: 02°43'09.1"S; verbatimLongitude: 54°40'20.7"W; **Event:** verbatimEventDate: 10.II.2020; **Record Level:** type: PhysicalObject; institutionCode: LETIA; basisOfRecord: PreservedSpecimen
- **Type status:**Other material. **Occurrence:** recordedBy: S.E. Santos; sex: 3 apterous ?, 3 apterous ?; **Location:** country: Brazil; stateProvince: Pará; municipality: Santarém; locality: Igarapé do Rai; verbatimLatitude: 02°43'09.1"S; verbatimLongitude: 54°40'20.7"W; **Event:** verbatimEventDate: 13.XI.2019; **Record Level:** type: PhysicalObject; institutionCode: LETIA; basisOfRecord: PreservedSpecimen
- **Type status:**Other material. **Occurrence:** recordedBy: S.E. Santos; sex: 2 apterous ?; **Location:** country: Brazil; stateProvince: Pará; municipality: Santarém; locality: Igarapé Guaraná; verbatimLatitude: 02°46'25.9"S; verbatimLongitude: 54°23'20.6"W; **Event:** verbatimEventDate: 06.III.2020; **Record Level:** type: PhysicalObject; institutionCode: LETIA; basisOfRecord: PreservedSpecimen
- **Type status:**Other material. **Occurrence:** recordedBy: S.E. Santos; sex: 2 macropterous ?; **Location:** country: Brazil; stateProvince: Pará; municipality: Santarém; locality: Igarapé Mararú; verbatimLatitude: 02°29'35.9"S; verbatimLongitude: 54°40'06.6"W; **Event:** verbatimEventDate: 23.VIII.2019; **Record Level:** type: PhysicalObject; institutionCode: LETIA; basisOfRecord: PreservedSpecimen
- **Type status:**Other material. **Occurrence:** recordedBy: S.E. Santos; sex: 6 apterous ?, 2 apterous ?; **Location:** country: Brazil; stateProvince: Pará; municipality: Santarém; locality: Igarapé Jatobá; verbatimLatitude: 02°34'17.9"S; verbatimLongitude: 54°51'36.8"W; **Event:** verbatimEventDate: 10.X.2020; **Record Level:** type: PhysicalObject; institutionCode: LETIA; basisOfRecord: PreservedSpecimen
- **Type status:**Other material. **Occurrence:** recordedBy: M. Galúcio; sex: 1 macropterous ?; **Location:** country: Brazil; stateProvince: Pará; municipality: Santarém; locality: Igarapé Mutunuy; verbatimLatitude: 02°28'53.1"S; verbatimLongitude: 54°41'45.9"W; **Event:** verbatimEventDate: 17.X.2015; **Record Level:** type: PhysicalObject; institutionCode: LETIA; basisOfRecord: PreservedSpecimen

#### Distribution

Bolivia, Brazil (Alagoas, Amazonas, Bahia, Maranhão, Mato Grosso, Minas Gerais, Pará, Pernambuco, Rondônia, São Paulo, Sergipe) (Moreira 2021a).

#### Notes

Previously recorded from Santarém (Kuitert 1942); first records from Belterra and Mojuí dos Campos.

#### Photograph

Fig. 6b

### Neogerris genticus

6CC75E81-B59A-51F8-851A-49AF6564435B

Limnogonus genticus – see Drake and Harris (1934): 213.
Neogerris genticus – see Andersen (1975): 8.

#### Materials

- **Type status:**Other material. **Occurrence:** recordedBy: S.E. Santos; sex: 1 apterous ?, 1 apterous ?; **Location:** country: Brazil; stateProvince: Pará; municipality: Belterra; locality: Igarapé Aramanaí; verbatimLatitude: 02°42'56.8"S; verbatimLongitude: 54°59'59.3"W; **Event:** verbatimEventDate: 07.XI.2019; **Record Level:** type: PhysicalObject; institutionCode: LETIA; basisOfRecord: PreservedSpecimen

#### Distribution

Brazil (Mato Grosso, Pará) (Moreira 2021a).

#### Notes

Described from Santarém (Drake and Harris 1934); first record from Belterra.

#### Photograph

Fig. 6c

### Neogerris lotus

B97056E9-F752-5003-ACF6-5576A0994E17

Limnogonus lotus – see White (1879a): 488.
Neogerris lotus – see Andersen (1975): 8.

#### Distribution

Brazil (Amazonas, Distrito Federal, Mato Grosso, Pará), Colombia, Guyana, Suriname, Trinidad and Tobago (Moreira 2021a).

#### Notes

Previously recorded from Santarém (Drake and Harris 1930), but absent from our samples.

### Neogerris lubricus

(White, 1879)

9743F075-DD52-5C75-9C81-1443861DC132

Limnogonus lubricus – see White (1879a): 489.
Neogerris lubricus – see Andersen (1975): 8.
Neogerris celeris – see Nieser and Alkins-Koo (1991): 71, figs. 172 and 174 (misidentification).
Neogerris lotus – see Cabette et al. (2010): 120 (misidentification).

#### Materials

- **Type status:**Other material. **Occurrence:** recordedBy: S.E. Santos; sex: 1 apterous ?, 3 apterous ?, 1 macropterous ?; **Location:** country: Brazil; stateProvince: Pará; municipality: Belterra; locality: Igarapé do Ailton; verbatimLatitude: 02°35'36.7"S; verbatimLongitude: 54°57'48.4"W; **Event:** verbatimEventDate: 06.XI.2019; **Record Level:** type: PhysicalObject; institutionCode: LETIA; basisOfRecord: PreservedSpecimen
- **Type status:**Other material. **Occurrence:** recordedBy: S.E. Santos; sex: 2 apterous ?, 3 apterous ?; **Location:** country: Brazil; stateProvince: Pará; municipality: Mojuí dos Campos; locality: Igarapé Água Fria; verbatimLatitude: 02°47'19.7"S; verbatimLongitude: 54°38'40.9"W; **Event:** verbatimEventDate: 24.IX.2020; **Record Level:** type: PhysicalObject; institutionCode: LETIA; basisOfRecord: PreservedSpecimen
- **Type status:**Other material. **Occurrence:** recordedBy: S.E. Santos; sex: 1 apterous ?; **Location:** country: Brazil; stateProvince: Pará; municipality: Mojuí dos Campos; locality: Igarapé Antonio Leite; verbatimLatitude: 03°09'06.2"S; verbatimLongitude: 54°50'28.7"W; **Event:** verbatimEventDate: 18.X.2019; **Record Level:** type: PhysicalObject; institutionCode: LETIA; basisOfRecord: PreservedSpecimen
- **Type status:**Other material. **Occurrence:** recordedBy: S.E. Santos; sex: 3 apterous ?, 3 macropterous ?, 1 macropterous ?; **Location:** country: Brazil; stateProvince: Pará; municipality: Mojuí dos Campos; locality: Igarapé Terra Preta; verbatimLatitude: 02°43'09.1"S; verbatimLongitude: 54°40'20.7"W; **Event:** verbatimEventDate: 10.II.2020; **Record Level:** type: PhysicalObject; institutionCode: LETIA; basisOfRecord: PreservedSpecimen
- **Type status:**Other material. **Occurrence:** recordedBy: S.E. Santos; sex: 1 apterous ?, 1 macropterous ?, 1 macropterous ?; **Location:** country: Brazil; stateProvince: Pará; municipality: Santarém; locality: Igarapé Sonrizal; verbatimLatitude: 02°32'13.6"S; verbatimLongitude: 54°55'26.6"W; **Event:** verbatimEventDate: 09.VIII.2019; **Record Level:** type: PhysicalObject; institutionCode: LETIA; basisOfRecord: PreservedSpecimen
- **Type status:**Other material. **Occurrence:** recordedBy: S.E. Santos; sex: 1 apterous ?; **Location:** country: Brazil; stateProvince: Pará; municipality: Santarém; locality: Igarapé Jatobá; verbatimLatitude: 02°34'17.9"S; verbatimLongitude: 54°51'36.8"W; **Event:** verbatimEventDate: 10.X.2020; **Record Level:** type: PhysicalObject; institutionCode: LETIA; basisOfRecord: PreservedSpecimen
- **Type status:**Other material. **Occurrence:** recordedBy: S.E. Santos; sex: 1 apterous ?; **Location:** country: Brazil; stateProvince: Pará; municipality: Santarém; locality: Igarapé Diamantino; verbatimLatitude: 02°30'16.2"S; verbatimLongitude: 54°39'32.9"W; **Event:** verbatimEventDate: 06.IX.2019; **Record Level:** type: PhysicalObject; institutionCode: LETIA; basisOfRecord: PreservedSpecimen

#### Distribution

Argentina, Bolivia, Brazil (Alagoas, Amapá, Amazonas, Bahia, Maranhão, Mato Grosso, Mato Grosso do Sul, Minas Gerais, Pará, Piauí, Rio de Janeiro, Rondônia, São Paulo, Sergipe), Colombia, Costa Rica, Ecuador, French Guiana, Guyana, Panama, Paraguay, Peru, Suriname, Trinidad and Tobago (Moreira 2021a).

#### Notes

Previously recorded from Santarém (Kuitert 1942, Nieser 1970); first records from Belterra and Mojuí dos Campos.

#### Photograph

Fig. 6d

### Neogerris visendus

(Drake & Harris, 1934)

28E74D2C-0D79-5E05-8123-D78BD36D998F

Limnogonus visendus – see Drake and Harris (1934): 215.
Neogerris visendus – see Andersen (1975): 8.

#### Materials

- **Type status:**Other material. **Occurrence:** recordedBy: S.E. Santos; sex: 3 apterous ?; **Location:** country: Brazil; stateProvince: Pará; municipality: Belterra; locality: Igarapé Aramanaí; verbatimLatitude: 02°42'56"S; verbatimLongitude: 54°59'59"W; **Event:** verbatimEventDate: 07.XI.2019; **Record Level:** type: PhysicalObject; institutionCode: LETIA; basisOfRecord: PreservedSpecimen
- **Type status:**Other material. **Occurrence:** recordedBy: S.E. Santos; sex: 1 macropterous ?, 1 macropterous ?; **Location:** country: Brazil; stateProvince: Pará; municipality: Santarém; locality: Igarapé Diamantino; verbatimLatitude: 02°30'16"S; verbatimLongitude: 54°39'32"W; **Event:** verbatimEventDate: 06.IX.2019; **Record Level:** type: PhysicalObject; institutionCode: LETIA; basisOfRecord: PreservedSpecimen
- **Type status:**Other material. **Occurrence:** recordedBy: S.E. Santos; sex: 6 macropterous ?, 4 macropterous ?; **Location:** country: Brazil; stateProvince: Pará; municipality: Santarém; locality: Igarapé Jatobá; verbatimLatitude: 02°34'17"S; verbatimLongitude: 54°51'36"W; **Event:** verbatimEventDate: 10.X.2020; **Record Level:** type: PhysicalObject; institutionCode: LETIA; basisOfRecord: PreservedSpecimen

#### Distribution

Brazil (Amazonas, Mato Grosso, Pará, Roraima), Colombia, Peru, Suriname, Venezuela (Moreira 2021a).

#### Notes

Previously recorded from Belterra (Nieser 1970); first records from Santarém.

#### Photograph

Fig. 6e

### Tachygerris adamsoni

(Drake, 1942)

E3A0ECE2-75BE-5F5A-8EDE-92EE7A52D3D7

Tenagogonus adamsoni – see Drake (1942): 108.
Tenagogonus duolineatus – see Kuitert (1942): 133, pl. X, figs. 4 and 4a (syn. by Drake 1957b: 193).
Tachygonus adamsoni – see Drake (1957a): 111.
Tachygerris adamsoni – see Drake (1957b): 193.

#### Materials

- **Type status:**Other material. **Occurrence:** recordedBy: S.E. Santos; sex: 1 macropterous ?; **Location:** country: Brazil; stateProvince: Pará; municipality: Santarém; locality: Cachoeira da Rocha Negra; verbatimLatitude: 02°29'48.5"S; verbatimLongitude: 54°45'13.3"W; **Event:** verbatimEventDate: 25.IX.2020; **Record Level:** type: PhysicalObject; institutionCode: LETIA; basisOfRecord: PreservedSpecimen

#### Distribution

Bolivia, Brazil (Alagoas, Amazonas, Maranhão, Mato Grosso, Minas Gerais, Pará, Piauí, Rio de Janeiro, Sergipe), Colombia, French Guiana, Paraguay, Peru, Suriname, Trinidad and Tobago, Venezuela (Moreira 2021a).

#### Notes

First record from the study area.

#### Photograph

Fig. 6f

### Rheumatobates crassifemur esakii

Schroeder, 1931

C66BDEB4-AAB8-5F95-9062-0517CAD19860

Rheumatobates crassifemur var. esakii – see Schroeder (1931): 77, pl. VII, fig. 3, pl. VIII, figs 3, 4.
Rheumatobates esakii – see Drake and Harris (1937): 362.
Rheumatobates crassifemur esakii – see Hungerford (1954): 565, pl. XII, fig. 22.
Rheumatobates bonariensis – see Nessimian et al. (2008) (misidentification).

#### Distribution

Brazil (Amazonas, Pará), Colombia, Ecuador, French Guiana, Guyana, Peru, Suriname, Trinidad and Tobago (Moreira 2021a).

#### Notes

Previously recorded from Santarém (Hungerford 1954), but absent from our samples.

### Rheumatobates klagei

Schroeder, 1931

9AF7E53B-BC1B-5881-AE06-2A89D058B921

**Figure 7.**
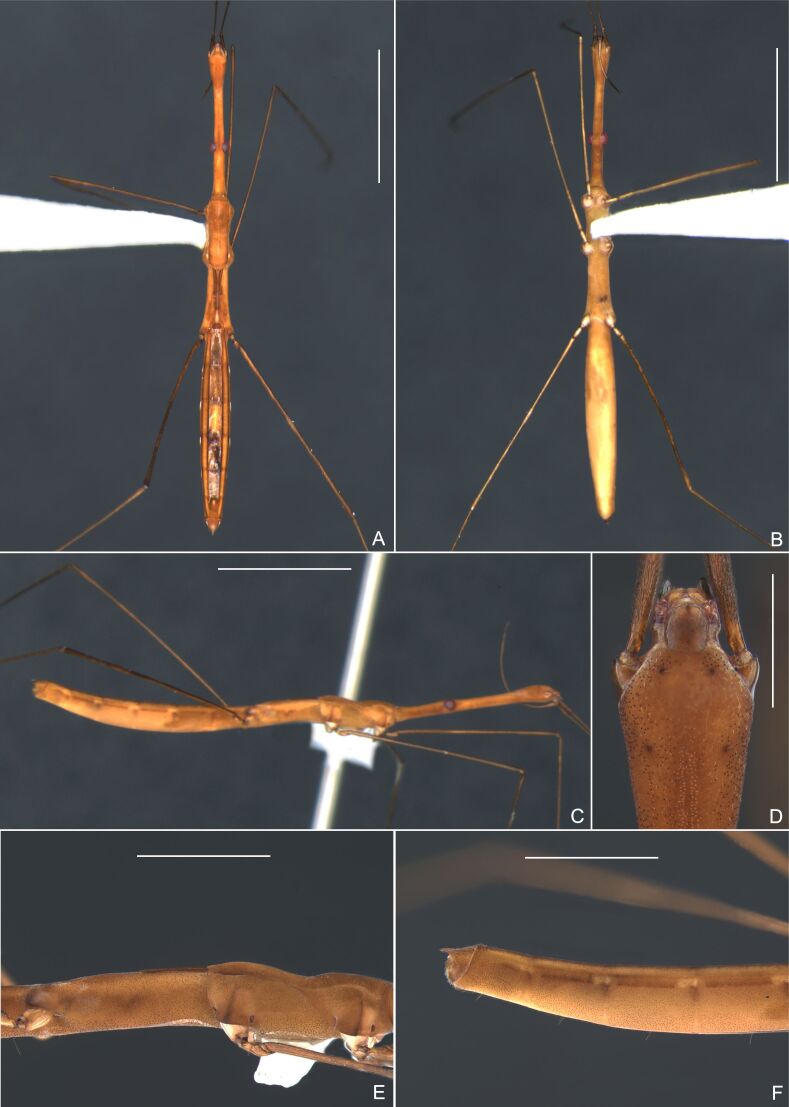
*Hydrometraargentina*, apterous female. **A.** Habitus, dorsal view; **B.** Habitus, ventral view; **C.** Habitus, lateral view; **D.** Anterior portion of head, dorsal view; **E.** Thorax, lateral view; **F.** Abdomen, lateral view. Scale bars: (A–C, E, F) 2 mm, (D) 0.2 mm.

**Figure 8.**
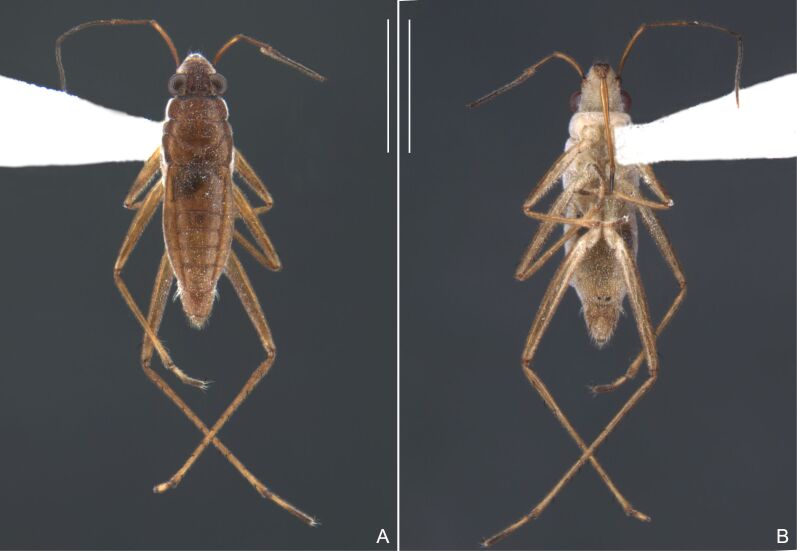
*Mesoveliamulsanti*, apterous male, habitus **A.** Dorsal view; **B.** Ventral view. Scale bars: 1 mm.

Rheumatobates klagei – see Schroeder (1931): 75, pl. VII, figs. 1 and 2, pl. VIII, fig. 5, pl. XI, figs. 7 and 8.

#### Distribution

Brazil (Amazonas, Pará), Peru (Moreira 2021a).

#### Notes

Previously recorded from Santarém (Hungerford 1954, Nieser 1970), but absent from our samples.

### Hydrometra argentina

Berg, 1879

05621B37-7233-5170-8BCD-6FBF9A2A770B

Hydrometra argentina – see Berg (1879): 182.
Hydrometra mensor – see White (1879b): 267 (syn. by Drake 1954: 61).
Limnobates chilensis – see Reed (1901): 70 (syn. by Drake 1953: 41).
Hydrometra kirkaldyana – see Torre-Bueno (1926): 104 (syn. by Drake 1953: 41).
Hydrometra husseyi – see Torre-Bueno (1926): 111 (syn. by Drake 1953: 41).
Hydrometra argenitna – see Drake and Lauck (1959): 51 (incorrect subsequent spelling).

#### Materials

- **Type status:**Other material. **Occurrence:** recordedBy: S.E. Santos; sex: 1 apterous ?; **Location:** country: Brazil; stateProvince: Pará; municipality: Belterra; locality: BR-163, Km-115; verbatimLatitude: 03°17'34.8"S; verbatimLongitude: 54°52'45.6"W; **Event:** verbatimEventDate: 25.IX.2020; habitat: igarapé; **Record Level:** type: PhysicalObject; institutionCode: LETIA; basisOfRecord: PreservedSpecimen
- **Type status:**Other material. **Occurrence:** recordedBy: E.C. Oliveira; sex: 1 apterous ?; **Location:** country: Brazil; stateProvince: Pará; municipality: Santarém; locality: Lago do Juá; verbatimLatitude: 02°25'57.8"S; verbatimLongitude: 54°46'55.0"W; **Event:** verbatimEventDate: 17.II.2020; **Record Level:** type: PhysicalObject; institutionCode: LETIA; basisOfRecord: PreservedSpecimen

#### Distribution

Argentina, Bolivia, Brazil (Alagoas, Amapá, Amazonas, Bahia, Espírito Santo, Mato Grosso, Mato Grosso do Sul, Minas Gerais, Pará, Paraíba, Paraná, Rio de Janeiro, Rio Grande do Sul, Santa Catarina, São Paulo, Sergipe), Chile, Colombia, Ecuador, Panama, Paraguay, Peru, Suriname, Trinidad and Tobago, Uruguay, Venezuela (Moreira 2021c).

#### Notes

Previously recorded from Santarém (Champion 1898; as *H.mensor*); first record from Belterra.

#### Photographs

Fig. 7

### Mesovelia mulsanti

White, 1879

A7C3DC42-4B90-5AAA-BE9C-5AFB9BBEB21D

Mesovelia mulsanti – see White (1879b): 268.
Mesovelia bisignata – see Uhler (1884): 274 (syn. by Champion (1898): 123).
Mesovelia mulsanti mulsanti – see Jaczewski (1930): 5, pl. III (syn. by Herring (1950): 148).
Mesovelia mulsanti bisignata – see Jaczewski (1930): 5, pl. I, figs. 1–5, pl. II, figs. 20 and 21, pl. III (syn. by Herring 1950: 148).
Mesovelia mulsanti meridionalis – see Jaczewski (1930): 6, pl. I, fig. 6, pl. II, fig. 19, pl. III (syn. by Herring 1950: 148).
Mesovelia mulsanti caraiba – see Jaczewski (1930): 6, pl. I, figs. 7–16, pl. II, figs. 17, 18 and 22, pl. III (syn. by Herring 1950: 148).

#### Materials

- **Type status:**Other material. **Occurrence:** recordedBy: S.E. Santos; sex: 1 apterous ?; **Location:** country: Brazil; stateProvince: Pará; municipality: Belterra; locality: Igarapé Aramanaí; verbatimLatitude: 02°42'56.8"S; verbatimLongitude: 54°59'59.3"W; **Event:** verbatimEventDate: 07.XI.2019; **Record Level:** type: PhysicalObject; institutionCode: LETIA; basisOfRecord: PreservedSpecimen
- **Type status:**Other material. **Occurrence:** recordedBy: S.E. Santos; sex: 4 apterous ?, 13 apterous ?; 1 macropterous ?; **Location:** country: Brazil; stateProvince: Pará; municipality: Belterra; locality: Igarapé Jatuarana; verbatimLatitude: 03°15'44.7"S; verbatimLongitude: 54°56'37.5"W; **Event:** verbatimEventDate: 11.II.2020; **Record Level:** type: PhysicalObject; institutionCode: LETIA; basisOfRecord: PreservedSpecimen
- **Type status:**Other material. **Occurrence:** recordedBy: S.E. Santos; sex: 4 apterous ?, 6 apterous ?, 2 ? with broken wings; **Location:** country: Brazil; stateProvince: Pará; municipality: Mojuí dos Campos; locality: Igarapé Terra Preta; verbatimLatitude: 02°43'09.1"S; verbatimLongitude: 54°40'20.7"W; **Event:** verbatimEventDate: 10.II.2020; **Record Level:** type: PhysicalObject; institutionCode: LETIA; basisOfRecord: PreservedSpecimen
- **Type status:**Other material. **Occurrence:** recordedBy: S.E. Santos; sex: 1 apterous ?, 1 apterous ?, 1 ? with broken wings; **Location:** country: Brazil; stateProvince: Pará; municipality: Mojuí dos Campos; locality: Igarapé Terra Preta; verbatimLatitude: 02°43'09.1"S; verbatimLongitude: 54°40'20.7"W; **Event:** verbatimEventDate: 24.II.2020; **Record Level:** type: PhysicalObject; institutionCode: LETIA; basisOfRecord: PreservedSpecimen
- **Type status:**Other material. **Occurrence:** recordedBy: S.E. Santos; sex: 1 apterous ?; **Location:** country: Brazil; stateProvince: Pará; municipality: Santarém; locality: Igarapé Mararú; verbatimLatitude: 02°29'35.9"S; verbatimLongitude: 54°40'06.6"W; **Event:** verbatimEventDate: 23.VIII.2019; **Record Level:** type: PhysicalObject; institutionCode: LETIA; basisOfRecord: PreservedSpecimen
- **Type status:**Other material. **Occurrence:** recordedBy: S.E. Santos; sex: 1 apterous ?, 1 apterous ?; **Location:** country: Brazil; stateProvince: Pará; municipality: Santarém; locality: Igarapé Diamantino; verbatimLatitude: 02°30'16.2"S; verbatimLongitude: 54°39'32.9"W; **Event:** verbatimEventDate: 06.IX.2019; **Record Level:** type: PhysicalObject; institutionCode: LETIA; basisOfRecord: PreservedSpecimen
- **Type status:**Other material. **Occurrence:** recordedBy: S.E. Santos; sex: 1 apterous ?, 1 apterous ?; **Location:** country: Brazil; stateProvince: Pará; municipality: Santarém; locality: Igarapé do Rai; verbatimLatitude: 02°35'35.3"S; verbatimLongitude: 54°30'18.1"W; **Event:** verbatimEventDate: 13.XI.2019; **Record Level:** type: PhysicalObject; institutionCode: LETIA; basisOfRecord: PreservedSpecimen
- **Type status:**Other material. **Occurrence:** recordedBy: S.E. Santos; sex: 4 apterous ?; **Location:** country: Brazil; stateProvince: Pará; municipality: Santarém; locality: Igarapé Guaraná; verbatimLatitude: 02°46'25.9"S; verbatimLongitude: 54°23'20.6"W; **Event:** verbatimEventDate: 06.III.2020; **Record Level:** type: PhysicalObject; institutionCode: LETIA; basisOfRecord: PreservedSpecimen
- **Type status:**Other material. **Occurrence:** recordedBy: S.E. Santos; sex: 1 apterous ?; **Location:** country: Brazil; stateProvince: Pará; municipality: Santarém; locality: Igarapé Mararú; verbatimLatitude: 02°29'35.9"S; verbatimLongitude: 54°40'06.6"W; **Event:** verbatimEventDate: 23.VIII.2019; **Record Level:** type: PhysicalObject; institutionCode: LETIA; basisOfRecord: PreservedSpecimen
- **Type status:**Other material. **Occurrence:** recordedBy: S.E. Santos; sex: 1 apterous ?, 1 macropterous ?; **Location:** country: Brazil; stateProvince: Pará; municipality: Santarém; locality: Igarapé Jatobá; verbatimLatitude: 02°34'17.9"S; verbatimLongitude: 54°51'36.8"W; **Event:** verbatimEventDate: 10.X.2020; **Record Level:** type: PhysicalObject; institutionCode: LETIA; basisOfRecord: PreservedSpecimen
- **Type status:**Other material. **Occurrence:** recordedBy: S.E. Santos; sex: 1 macropterous ?; **Location:** country: Brazil; stateProvince: Pará; municipality: Santarém; verbatimLatitude: 02°27'32.6"S; verbatimLongitude: 54°44'48.4"W; **Event:** verbatimEventDate: 14.IV.2020; habitat: puddle; **Record Level:** type: PhysicalObject; institutionCode: LETIA; basisOfRecord: PreservedSpecimen
- **Type status:**Other material. **Occurrence:** recordedBy: E.C. Oliveira; sex: 4 apterous ?, 1 macropterous ?; **Location:** country: Brazil; stateProvince: Pará; municipality: Santarém; locality: Lago Mapiri; verbatimLatitude: 02°25'28.5"S; verbatimLongitude: 54°44'47.7"W; **Event:** verbatimEventDate: 18.II.2020; **Record Level:** type: PhysicalObject; institutionCode: LETIA; basisOfRecord: PreservedSpecimen
- **Type status:**Other material. **Occurrence:** recordedBy: M. Galúcio; sex: 2 apterous ?; **Location:** country: Brazil; stateProvince: Pará; municipality: Santarém; locality: Igarapé Vila Nova; verbatimLatitude: 02°30'50.4"S; verbatimLongitude: 54°49'29.7"W; **Event:** verbatimEventDate: 10.X.2015; **Record Level:** type: PhysicalObject; institutionCode: LETIA; basisOfRecord: PreservedSpecimen

#### Distribution

Antigua and Barbuda, Argentina, Aruba, Barbados, Belize, Bolivia, Bonaire, Brazil (Alagoas, Amapá, Amazonas, Bahia, Ceará, Espírito Santo, Goiás, Maranhão, Mato Grosso, Mato Grosso do Sul, Minas Gerais, Pará, Paraná, Pernambuco, Piauí, Rio de Janeiro, Rio Grande do Sul, Rondônia, Santa Catarina, São Paulo, Sergipe), Canada, Colombia, Costa Rica, Cuba, Curaçao, Dominica, Dominican Republic, French Guiana, Grenada, Guadeloupe, Guatemala, Guyana, Hawaiian Islands, Honduras, Jamaica, Klein Curaçao, Mexico, Nicaragua, Panama, Paraguay, Peru, Puerto Rico, St. Kitts and Nevis, St. Lucia, St. Martin, St. Vincent and the Grenadines, Trinidad and Tobago, United States, U.S. Virgin Islands, Venezuela (Moreira 2021d).

#### Notes

Previously recorded from Santarém (Neering 1954); first records from Belterra and Mojuí dos Campos.

#### Photographs

Fig. 8

### Mesovelia zeteki

Harris & Drake, 1941

DA62EEA7-0CFD-5D1E-95A9-40AE6F29CAF4

Mesovelia zeteki – see Harris and Drake (1941): 276.

#### Distribution

Brazil (Amapá, Amazonas, Pará), Colombia, Panama (Moreira 2021d)

#### Notes

Previously recorded from Santarém (Moreira et al. 2008), but absent from our samples.

### Microvelia aschnakiranae

Makhan, 2014

98913E5C-B483-52A5-8990-D82034D1F34C

Microvelia aschnakiranae – see Makhan (2014): 2, figs. 7 and 8.

#### Materials

- **Type status:**Other material. **Occurrence:** recordedBy: S.E. Santos; sex: 1 macropterous ?; **Location:** country: Brazil; stateProvince: Pará; municipality: Santarém; locality: Igarapé Guaraná; verbatimLatitude: 02°46'25.9"S; verbatimLongitude: 54°23'20.6"W; **Event:** verbatimEventDate: 06.III.2020; **Record Level:** type: PhysicalObject; institutionCode: LETIA; basisOfRecord: PreservedSpecimen

#### Distribution

Brazil (Pará), Suriname (Makhan 2014).

#### Notes

First record from Brazil.

#### Photographs

Fig. 9

### Microvelia belterrensis sp. n.

47238AB1-AFE8-55BB-B917-3598EC46D13B

ED4F8A12-EA0B-494B-9AA0-008870A3F797

#### Materials

- **Type status:**Holotype. **Occurrence:** recordedBy: S.E. Santos; sex: apterous ?; **Location:** country: Brazil; stateProvince: Pará; municipality: Belterra; locality: BR-163, Km-115; verbatimLatitude: 03°17'34.8"S; verbatimLongitude: 54°52'45.6"W; **Event:** verbatimEventDate: 25.IX.2020; habitat: igarapé; **Record Level:** type: PhysicalObject; institutionCode: CEIOC 81770; basisOfRecord: PreservedSpecimen
- **Type status:**Paratype. **Occurrence:** recordedBy: S.E. Santos; sex: 1 apterous ?, 3 apterous ?; **Location:** country: Brazil; stateProvince: Pará; municipality: Belterra; locality: BR-163, Km-115; verbatimLatitude: 03°17'34.8"S; verbatimLongitude: 54°52'45.6"W; **Event:** verbatimEventDate: 25.IX.2020; habitat: igarapé; **Record Level:** type: PhysicalObject; institutionCode: CEIOC 81771; basisOfRecord: PreservedSpecimen

#### Description

##### Apterous males

Holotype/Paratype. BL 1.15/1.2, HL 0.22/0.21, HW 0.39/0.38, ANT I 0.14/0.14, ANT II 0.10/0.09, ANT III 0.13/0.13, ANT IV 0.20/0.22, INT 0.18/0.7, EYE 0.09/0.09, PL 0.13/0.14, PW 0.48/0.51; FORE LEG: FEM 0.28/0.31, TIB 0.24/0.24, TAR I 0.15/0.15; MID-LEG: FEM 0.36/0.36, TIB 0.29/0.29, TAR I 0.06/0.05, TAR II 0.11/0.11; HIND LEG: FEM 0.42/0.42, TIB 0.42/0.42, TAR I 0.05/0.5, TAR II 0.11/0.11.

Head dark-brown. Antennomere I yellowish, II–IV dark-brown. Eye reddish-brown. Labium yellowish-brown, except apex of article III and entire IV dark-brown. Pronotum with anterior half and lateral margins orange-brown, apical half dark-brown. Meso- and metanota dark brown. Prosternum yellowish-brown, darker at middle groove. Meso- and metasterna dark-brown. Pro- and metacetabula with anterior half yellowish-brown and posterior half dark brown. Anterior and posterior coxae and trochanters yellowish-brown. Mesoacetabulum and middle coxa dark-brown. Femora and tibiae yellowish-brown, darker dorsally on distal third; tarsi dark-brown. Abdominal mediotergites dark-brown. Abdominal laterotergites orange-brown with lateral margins dark. Abdominal sterna dark-brown. Terminalia yellowish-brown.

Head covered with very short setae, longer on clypeus. Antenna covered with short setae. Antennomere I widest, slightly curved laterally, thickened towards apex; II wider than III–IV, thickened towards apex; III cylindrical, thinner than IV; IV fusiform, at middle subequal to II in thickness. Labium reaching base of mesosternum.

**Figure 10.**
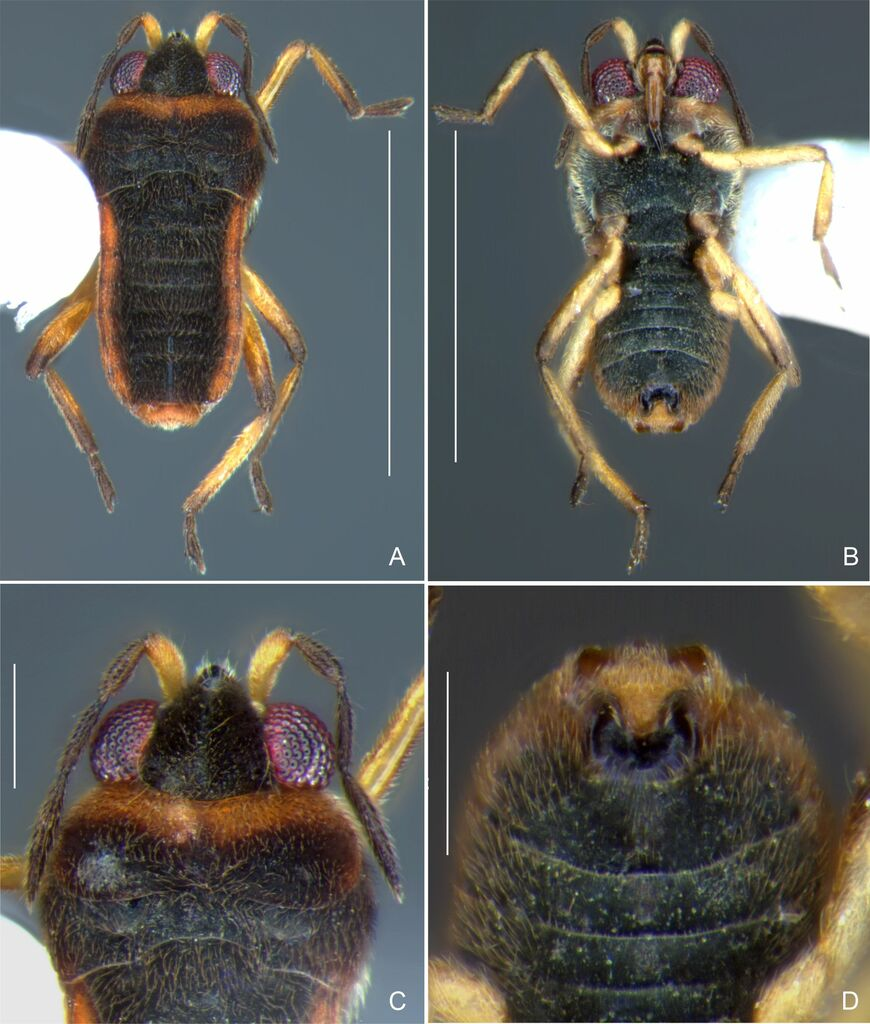
*Microveliabelterrensis***sp. nov.**, apterous male, holotype **A.** Habitus, dorsal view; **B.** Habitus, ventral view; **C.** Head and thorax, dorsal view; **D.** Abdomen, segments IV–VII and terminalia. Scale bars: (A, B) 1 mm, (C, D) 0.2 mm.

Thoracic terga densely covered with moderately long, light setae; sides of thorax, prosternum and acetabula with denser, longer setae; meso- and metasterna with short setae. Pronotum with lateral margins bowed; posterior margin sinuous, concave centrally, exposing two rounded lobes of the mesonotum laterally (Fig. 10C). Metanotum exposed as a short central stripe, with posterior margin widely concave. Legs covered with short setae, with some longer setae on dorsal surfaces of femora and tibiae. Femora without spines. Fore tibia straight, enlarged towards apex, with an obtuse, short spine at apex. Hind femur slightly thicker than middle femur. Hind tibia straight.

**Figure 11.**
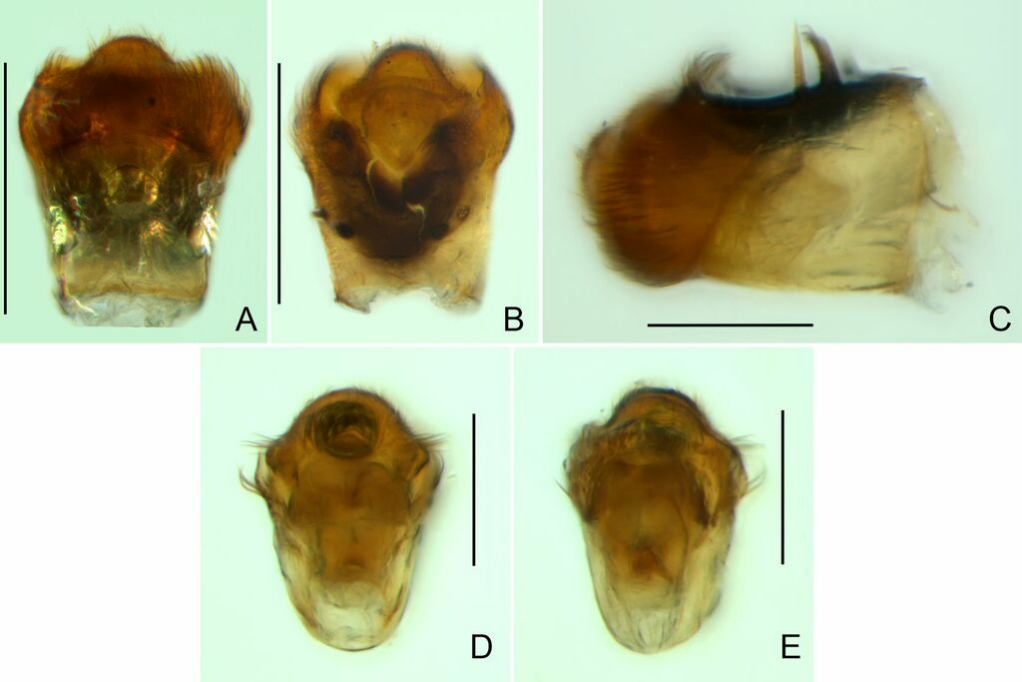
*Microveliabelterrensis***sp. nov.**, male terminalia **A.** Dorsal view; **B.** Ventral view [abdominal segment VIII broken]; **C.** Lateral view; **D, E** Genital capsule: **D.** Dorsal view; **E.** Ventral view. Scale bars: (A, B and E) 0.1 mm, (C and D) 0.2 mm.

Abdominal mediotergites covered with moderately long setae. Mediotergites III–VI depressed; VI–VII with shiny median stripe; VII with posterior margin slightly concave. Abdominal laterotergites elevated to about 90°. Abdominal sterna covered with short setae, posterior margins concave, without tubercles. Abdominal segment VIII slightly exposed, dorsally more than four times wider than long; dorsum with lateral margins convergent and posterior margin slightly concave (Fig. 10A and Fig. 11A); ventre strongly sclerotised, with an evident central notch (Fig. 10B, D and Fig. 11B) and three tufts of long setae on each side (well visible in lateral view, Fig. 11C). Proctiger with lateral projections and apical margin rounded (Fig. 11D); parameres symmetrical (Fig. 11E).

##### Apterous females

BL 1.21–1.26, HL 0.22, HW 0.40–0.42, ANT I 0.14, ANT II 0.08–0.10, ANT III 0.12, ANT IV 0.20–0.22, INT 0.18, EYE 0.09–0.10, PL 0.12–0;14, PW 0.54–0.58; FORE LEG: FEM 0.30, TIB 0.24, TAR I 0.14–0.16; MID-LEG: FEM 0.36, TIB 0.28–0.30, TAR I 0.06, TAR II 0.12; HIND LEG: FEM 0.40–0.42, TIB 0.40, TAR I 0.06, TAR II 0.12.

**Figure 12.**
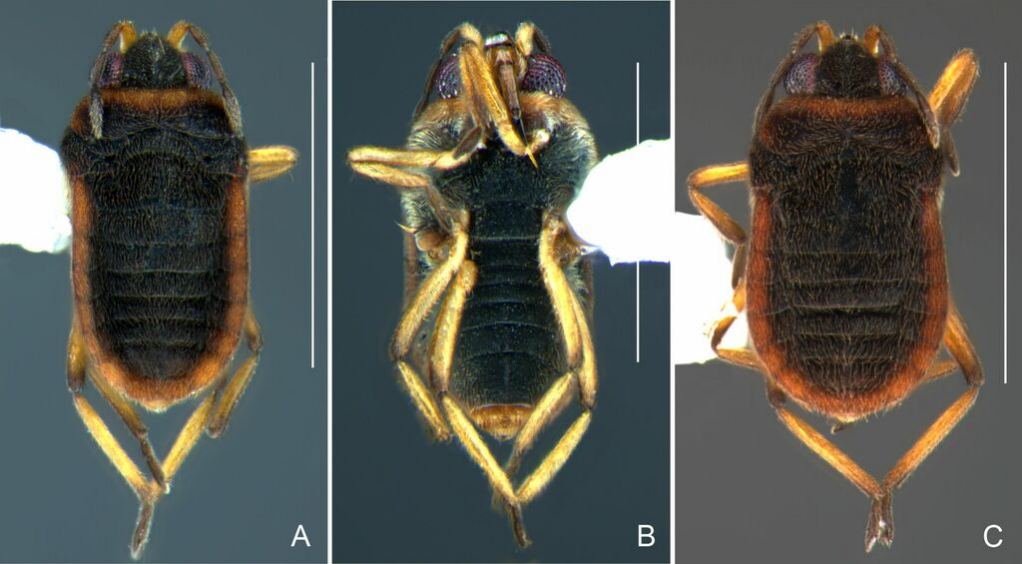
*Microveliabelterrensis***sp. nov.**, apterous females, paratypes **A.** Habitus, dorsal view; **B.** Habitus, ventral view; **C.** Habitus, dorsal view; expanded abdomen, probably with eggs. Scale bars: 1 mm.

Colouration and structure similar to apterous male, but larger and more robust (Fig. 12). Fore tibia straight, enlarged towards apex, without apical spine. Abdomen larger than in in males, as wide as pronotum (Fig. 12A). Abdominal segment VIII yellowish-brown dorsally and ventrally. Posterior margin of abdominal mediotergite VIII straight. Abdominal laterotergites elevated to about 45º.

#### Diagnosis

This new species can be distinguished from other South American *Microvelia* Westwood, 1834 by the pronotum of the apterous form covering the mesonotum centrally, but exposing it laterally; the metanotum exposed centrally, with the posterior margin widely concave; the abdominal segment VIII of the males slightly exposed dorsally, more than four times wider than long, with the lateral margins convergent and the posterior margin slightly concave, ventrally deeply inserted into the pregenital abdomen, strongly sclerotised, with an evident central notch and three tufts of long setae on each side (only one tuft is visible before dissection); and the male proctiger with small, rounded, lateral projections. Additionally, *M.belterrensis* **sp. nov.** has the body shorter than 1.30 mm (apterous males 1.15–1.20 mm; apterous females 1.21–1.26 mm).

*Microveliavenustatis* Drake & Harris, 1933 (Fig. 13a, b, c; apterous male in Rodrigues et al. 2021: fig. 89) and *M.hinei* Drake, 1920 (Fig. 13d, e, f) are small species that share with *M.belterrensis* **sp. nov.** the general appearance and the male terminalia strongly inserted into the pregenital abdomen, although more deeply than in the new species and more weakly sclerotised. In contrast, *M.venustatis* displays a thicker antennomere IV and the abdominal sterna of the male bear tufts of setae medially (at least on segments V–VII; Fig. 13b), which are absent in the other two species. *Microveliahinei* can be distinguished from both by the pronotum of the apterous form completely covering the mesonotum, exposing only the metanotum centrally, the slightly longer body (paratype apterous male 1.40 mm), the different colour pattern and the posterior margin of the male abdominal segment VIII nearly straight dorsally (vs. concave in *M.belterrensis* **sp. nov.**).

*Microveliaubatuba* Moreira & Barbosa, 2011, in turn, shares with the new species the male abdominal segment VIII notched ventrally. Although *M.ubatuba* is known only from macropterous specimens, it can be distinguished from *M.belterrensis* **sp. nov.** by the longer and narrower male abdominal segment VIII, dorsally with a distinct concavity at the posterior margin and ventrally with a strong rounded depression and a smaller and weakly sclerotised notch on the posterior margin (Moreira and Barbosa 2011: 299, figs. 7–11).

Finally, the male proctiger with lateral projections was previously reported in two other Neotropical species, *M.mimula* White, 1879 and *M.quieta* Drake & Carvalho, 1954 (Drake and Carvalho 1954: 224, figs. 1 and 2). Both are easily distinguished from *M.belterrensis* **sp. nov.** by the longer body and the well-exposed male terminalia.

#### Etymology

This species is named after Belterra Municipality, where the type-series was collected.

### Microvelia hamadae sp. n.

DA7E9E4B-7B8D-5139-B575-824F0E90539F

8C5F8ACA-BE1F-461E-9FB1-1D23C55B7DBF

#### Materials

- **Type status:**Holotype. **Occurrence:** recordedBy: S.E. Santos; sex: apterous ?; **Location:** country: Brazil; stateProvince: Pará; municipality: Belterra; locality: BR-163, Km-115; verbatimLatitude: 03°17'34.8"S; verbatimLongitude: 54°52'45.6"W; **Event:** verbatimEventDate: 25.IX.2020; habitat: igarapé; **Record Level:** type: PhysicalObject; institutionCode: CEIOC 81767; basisOfRecord: PreservedSpecimen
- **Type status:**Paratype. **Occurrence:** recordedBy: S.E. Santos; sex: 1 apterous ?, 1 macropterous ?, 2 apterous ?; **Location:** country: Brazil; stateProvince: Pará; municipality: Belterra; locality: BR-163, Km-115; verbatimLatitude: 03°17'34.8"S; verbatimLongitude: 54°52'45.6"W; **Event:** verbatimEventDate: 25.IX.2020; habitat: igarapé; **Record Level:** type: PhysicalObject; institutionCode: CEIOC 81768; basisOfRecord: PreservedSpecimen
- **Type status:**Paratype. **Occurrence:** recordedBy: S.E. Santos; sex: 1 macropterous ?; **Location:** country: Brazil; stateProvince: Pará; municipality: Belterra; locality: BR-163, Km-115; verbatimLatitude: 03°17'34.8"S; verbatimLongitude: 54°52'45.6"W; **Event:** verbatimEventDate: 20.I.2020; habitat: igarapé; **Record Level:** type: PhysicalObject; institutionCode: CEIOC 81769; basisOfRecord: PreservedSpecimen

#### Description

##### Apterous males

Holotype/Paratype. BL 1.23/1.24, HL 0.22/0.22, HW 0.38/0.38, ANT I 0.16/0.16, ANT II 0.10/0.10, ANT III 0.12/0.12, ANT IV 0.24/0.24, INT 0.18/0.18, EYE 0.09/0.09, PL 0.20/0.20, PW 0.46/0.48; FORE LEG: FEM 0.32/0.32, TIB 0.24/0.24, TAR I 0.16/0.16; MID-LEG: FEM 0.40/0.38, TIB 0.28/0.28, TAR I 0.06/0.06, TAR II 0.12/0.12; HIND LEG: FEM 0.40/0.42, TIB 0.40/0.42, TAR I 0.06/0.06, TAR II 0.13/–.

**Figure 14.**
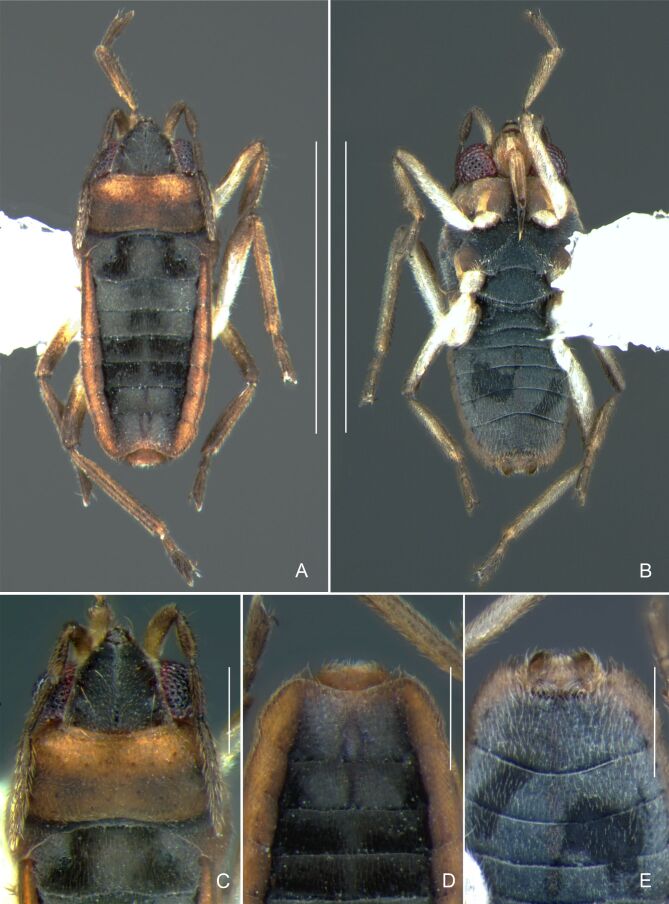
*Microveliahamadae***sp. nov.**, apterous male, holotype **A.** Habitus, dorsal view; **B.** Habitus, ventral view; **C.** Head and thorax, dorsal view; **D, E** Abdomen, segments IV–VII and terminalia: **D.** Dorsal view **E.** Ventral view. Scale bars: (A and B) 1 mm, (C–E) 0.2 mm.

Head black. Antenna dark-brown, proximal half of antennomere I yellowish-brown. Eye reddish-brown. Labium yellowish-brown, except for distal article dark-brown. Pronotum dark-brown with a medially interrupted yellowish-brown transverse band on anterior half (Fig. 14A). Metanotum black. Prosternum yellowish-brown, median groove dark. Meso- and metasterna black. Acetabula brown. Coxae, trochanters and proximal half and ventre of femora pale yellow; remainder of legs brown. Abdominal mediotergites black with whitish pruinosity; I with two darker longitudinal spots sublaterally; II–III darker at middle; IV–V dark with whitish pruinosity at median stripe and posterior third; VI–VII darker at one/two narrow median stripe(s). Abdominal laterotergites yellowish-brown, darker at anterior third and mesal and lateral margins. Abdominal sterna black (Fig. 14B). Terminalia yellowish-brown.

Head covered with short setae, longer on clypeus. Antenna reaching apex of metanotum; covered with short setae, longer and denser on article IV. Antennomere I widest, slightly curved laterally, thickened towards apex; II wider than III–IV, thickened towards apex; III cylindrical, thinner than IV; IV fusiform. Labium reaching middle of mesosternum.

Thoracic terga densely covered with very short setae; sides of thorax, prosternum and acetabula with longer setae. Pronotum long, covering mesonotum and most of metanotum; metanotum visible only as a very short central stripe (Fig. 14C). Circular punctures on a centrally interrupted transverse row adjacent to anterior margin of pronotum; another transverse row posterior to yellowish band; several sparse punctures posteriorly to latter row. Legs covered with short setae, with some longer setae on dorsal surfaces of femora and tibiae. Femora without spines. Fore tibia straight, enlarged towards apex, with an obtuse, short spine at apex. Hind femur thicker than middle femur. Hind tibia straight.

**Figure 15.**
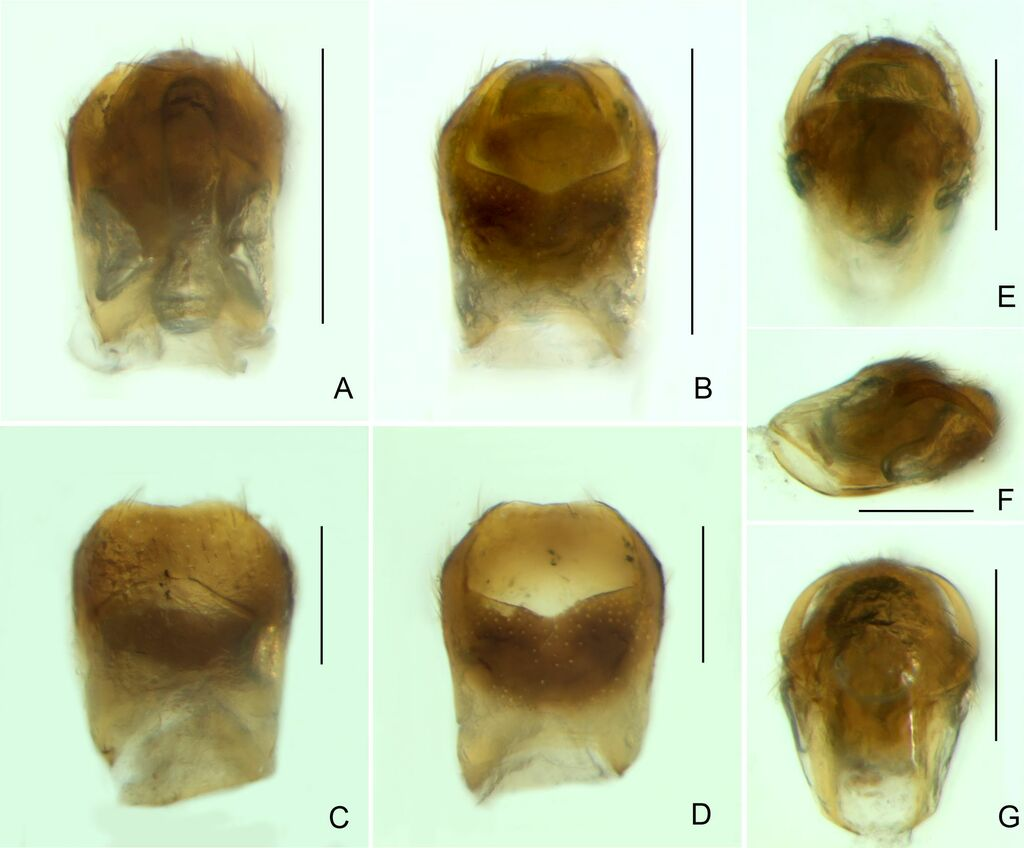
*Microveliahamadae***sp. nov.**, male terminalia **A.** Dorsal view; **B.** Ventral view; **C, D** Abdominal segment VIII: **C.** Dorsal view; **D.** Ventral view; **E–G** Genital capsule: **E.** Dorsal view; **F.** Lateral view; **G.** Ventral view. Scale bars: (A and B) 0.2 mm, (C–G) 0.1 mm.

Abdomen covered with short setae. Posterior margin of abdominal mediotergite I with a slight concavity at middle; VII with a pronounced concavity (Fig. 14D). Abdominal laterotergites elevated to about 45º. Abdominal sterna II–IV moderately short, V–VI longer, VII twice as long as II; posterior margins concave, without tubercles, with many long setae (Fig. 14E). Abdominal segment VIII deeply inserted into pregenital abdomen; partially visible dorsally due to the concavity of mediotergite VII; faintly visible ventrally (Fig. 14D, E); posterior margin dorsally concave, ventrally with a weak median notch (Fig. 15A–D). Proctiger without lateral projections (Fig. 15E); parameres symmetrical (Fig. 15G); shape as in Fig. 15F.

##### Macropterous males

BL 1.42–1.44, HL 0.22–0.23, HW 0.40, ANT I 0.16, ANT II 0.10, ANT III 0.12, ANT IV 0.24–0.26, INT 0.18, EYE 0.08–0.10, PL 0.46–0.48, PW 0.66; FORE LEG: FEM 0.32, TIB 0.24, TAR I 0.16; MID-LEG: FEM 0.38, TIB 0.30, TAR I 0.04–0.06, TAR II 0.12; HIND LEG: FEM 0.42–0.44, TIB 0.44–0.46, TAR I 0.06, TAR II 0.12.

**Figure 16.**
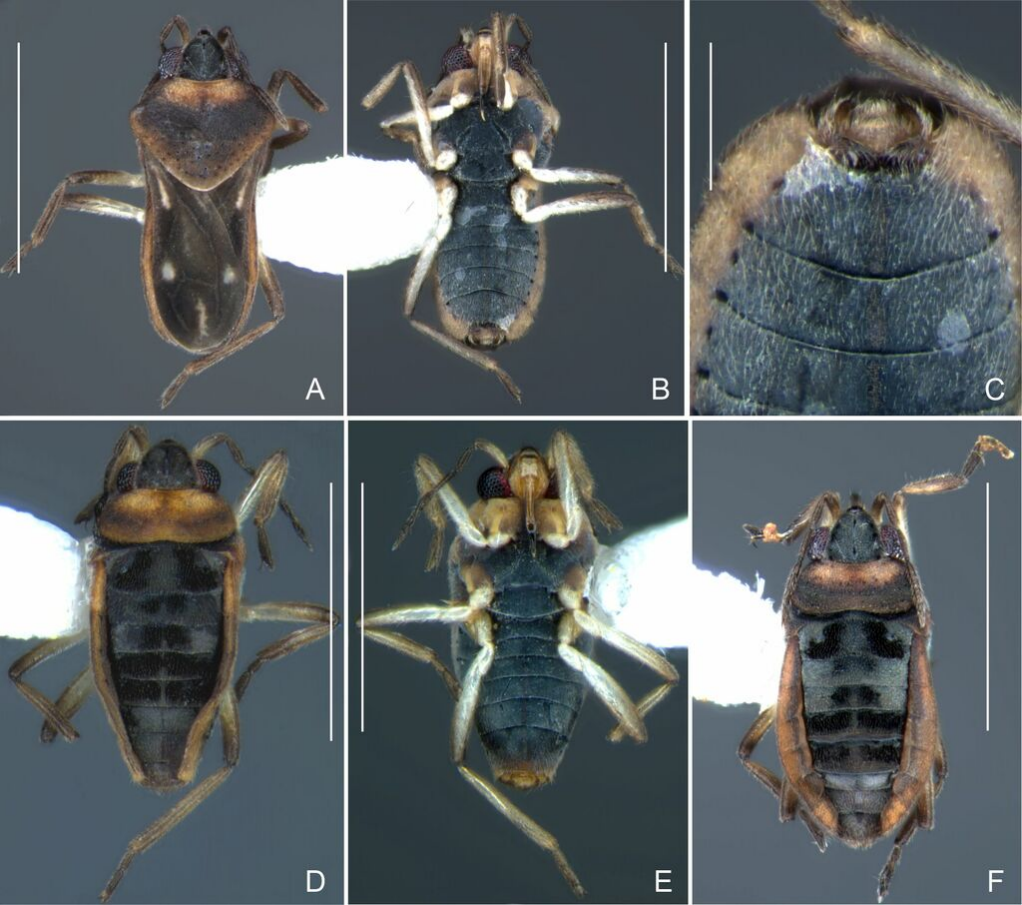
*Microveliahamadae***sp. nov.**, paratypes **A–C** Macropterous male: **A.** Habitus, dorsal view; **B.** Habitus, ventral view; **C.** Abdominal segments V–VII and terminalia, ventral view; **D–F** Apterous females: **D.** Habitus, dorsal view; **E.** Habitus, ventral view; **F** Habitus, dorsal view; abdomen with different shape, probably with eggs. Scale bars: (A, B and D–F) 1 mm, (C) 0.2 mm.

Colouration and structure similar to apterous male (Fig. 16A–C). Pronotum long, subpentagonal, completely covering meso- and metanota; posterior margin rounded. Fore wings dark brown, with a proximal pair of elongated whitish maculae, a distal pair of rounded whitish maculae and a median whitish macula close to apex; lateral margins with rows of white setae.

##### Apterous females

BL 1.36–1.42, HL 0.22–0.24, HW 0.40, ANT I 0.16, ANT II 0.10, ANT III 0.12, ANT IV 0.26, INT 0.18–0.20, EYE 0.09, PL 0.20, PW 0.52–0.54; FORE LEG: FEM 0.34, TIB 0.26, TAR I 0.18; MID-LEG: FEM 0.40, TIB 0.30–0.31, TAR I 0.06, TAR II 0.12; HIND LEG: FEM 0.44, TIB 0.46, TAR I 0.06, TAR II 0.14.

Colouration and structure similar to apterous male, but larger and more robust (Fig. 16D–F). Fore tibia straight, enlarged towards apex, without apical spine. Posterior margins of abdominal mediotergite VII and sternum VII straight. Posterior margin of abdominal tergum VIII rounded, with long setae. Abdominal laterotergites elevated to about 90º, slightly bowed on sides of mediotergites II–V, convergent and slightly reflected on sides of VI–VIII (Fig. 16D). Possibly fecundated females display expanded abdomen with differently shaped laterotergites (Fig. 16F).

#### Diagnosis

This new species can be distinguished from other South American *Microvelia* by the pronotum of apterous specimens long, covering the mesonotum and most of the metanotum; the metanotum visible only as a very short central stripe; the posterior margin of the male abdominal mediotergite VII with a pronounced concavity; the male abdominal segment VIII deeply inserted into the pregenital abdomen, with the posterior margin dorsally concave and ventrally with a weak median notch; the shape of the female abdomen, with abdominal laterotergites elevated to about 90º, slightly bowed on the sides of mediotergites II–V, convergent and slightly reflected on the sides of VI–VIII; and by the pattern of whitish maculae on the fore wings of macropterous specimens: a proximal pair of elongated maculae, a distal pair of rounded maculae and a median macula close to apex. Additionally, *M.hamadae* **sp. nov.** has the body shorter than 1.50 mm (apterous males 1.23–1.24 mm; macropterous males 1.42–1.44 mm; apterous females 1.36–1.42 mm).

*Microveliahamadae***sp. nov.** is very different from other Neotropical species of *Microvelia* with known apterous forms in which the pronotum covers the mesonotum and the metanotum completely or almost completely (e.g. *M.argentata* Nieser & Alkins-Koo, 1991, *M.digitalis* Padilla-Gil, 2019, *M.hambletoni* Drake, 1951, *M.hormiga* Padilla-Gil, 2019, *M.ioana* Drake & Hottes, 1952, *M.limaiana* Drake, 1951, *M.micra* Padilla-Gil, 2019, *M.nelsoni* Moreira, Barbosa & Ribeiro, 2012, *M.potama* Drake, 1958, *M.recifana* Drake, 1951 and *M.reflexa* Polhemus, 1974), because the general shape is distinct and they all have the male terminalia well-exposed, differently from the new species. The females of *M.ioana, M.micra* and *M.reflexa* share with *M.hamadae* **sp. nov.** the abdominal laterotergites reflected over the mediotergites, but the general colour of the body and the shape of the pronotum are quite different from the new species.

The macropterous form of the new species can be distinguished from the small South American species with known macropterous forms (e.g. *M.hinei, M. lujanana* Drake, 1951, *M.munda* Drake, 1951, *M.pudoris* Drake & Harris, 1936, *M.summersi* Drake & Harris, 1928 and *M.venustatis*) because of the pattern of maculae on the fore wings, together with the strongly inserted male terminalia and the shape of the male pygophore, proctiger and parameres.

#### Etymology

This species is named in honour of Dr. Neusa Hamada (Instituto Nacional de Pesquisas da Amazônia, Manaus), one of the most important Brazilian aquatic entomologists.

### Microvelia longipes

Uhler, 1894

A899B821-5E29-5AAF-859F-9523472BA441

Microvelia longipes – see Uhler (1894): 219.
Microvelia modesta – see Uhler (1894): 220 (syn. by Drake 1952: 13).

#### Materials

- **Type status:**Other material. **Occurrence:** recordedBy: S.E. Santos; sex: 1 macropterous ?; **Location:** country: Brazil; stateProvince: Pará; municipality: Santarém; locality: Cachoeira da Cavada; verbatimLatitude: 02°35'48.9"S; verbatimLongitude: 54°31'47.3"W; **Event:** verbatimEventDate: 13.XI.2019; **Record Level:** type: PhysicalObject; institutionCode: LETIA; basisOfRecord: PreservedSpecimen
- **Type status:**Other material. **Occurrence:** recordedBy: S.E. Santos; sex: 1 macropterous ?; **Location:** country: Brazil; stateProvince: Pará; municipality: Santarém; verbatimLatitude: 02°27'32.6"S; verbatimLongitude: 54°44'48.4"W; **Event:** verbatimEventDate: 14.IV.2020; habitat: puddle; **Record Level:** type: PhysicalObject; institutionCode: LETIA; basisOfRecord: PreservedSpecimen

#### Distribution

Argentina, Aruba, Barbados, Bolivia, Bonaire, Brazil (Alagoas, Amazonas, Bahia, Espírito Santo, Mato Grosso do Sul, Minas Gerais, Rio de Janeiro, Roraima, Santa Catarina, São Paulo), Colombia, Cuba, Curaçao, Dominican Republic, Ecuador, French Guiana, Grenada, Guyana, Jamaica, Paraguay, Peru, Puerto Rico, Saint Barthélemy, St. Eustatius, St. Kitts and Nevis, St. Martin, U.S. Virgin Islands, Trinidad and Tobago, Venezuela (Moreira 2021e).

#### Notes

First records from Pará State.

#### Photograph

Fig. 17a

### Microvelia mimula

White, 1879

90B450B1-BB58-58A5-8F10-D82E419B8D80

Microvelia mimula – see White (1879a): 487.
Microvelia capitata – see Uhler (1894): 218 (misidentification).
Microvelia mendozana – see Jensen-Haarup (1920): 220, fig. 4 (syn. by Drake and Hussey 1955: 114).
Microvelia myersi – see McKinstry (1937): 32 (syn. by Drake and Hussey 1951: 144).
Microvelia aemulana – see Drake and Plaumann (1955): 23 (syn. by Moreira and Barbosa 2011: 306).
Microvelia amrishi – see Makhan (2014): 2, figs. 4–6 (syn. by Aristizábal-García et al. 2015: 596).

#### Materials

- **Type status:**Other material. **Occurrence:** recordedBy: S.E. Santos; sex: 1 macropterous ?, 1 macropterous ?; **Location:** country: Brazil; stateProvince: Pará; municipality: Mojuí dos Campos; locality: Igarapé Mojuí dos Caboclos; verbatimLatitude: 02°42'03.0"S; verbatimLongitude: 54°41'01.0"W; **Event:** verbatimEventDate: 02.X.2020; **Record Level:** type: PhysicalObject; institutionCode: LETIA; basisOfRecord: PreservedSpecimen
- **Type status:**Other material. **Occurrence:** recordedBy: S.E. Santos; sex: 2 macropterous ?, 2 macropterous ?; **Location:** country: Brazil; stateProvince: Pará; municipality: Santarém; verbatimLatitude: 02°27'32.6"S; verbatimLongitude: 54°44'48.4"W; **Event:** verbatimEventDate: 10.IV.2020; habitat: puddle; **Record Level:** type: PhysicalObject; institutionCode: LETIA; basisOfRecord: PreservedSpecimen
- **Type status:**Other material. **Occurrence:** recordedBy: S.E. Santos; sex: 1 macropterous ?; **Location:** country: Brazil; stateProvince: Pará; municipality: Santarém; locality: Igarapé Guaraná; verbatimLatitude: 02°46'25.9"S; verbatimLongitude: 54°23'20.6"W; **Event:** verbatimEventDate: 06.III.2020; **Record Level:** type: PhysicalObject; institutionCode: LETIA; basisOfRecord: PreservedSpecimen

#### Distribution

Argentina, Barbados, Brazil (Alagoas, Amazonas, Ceará, Espírito Santo, Maranhão, Mato Grosso, Mato Grosso do Sul, Minas Gerais, Pará, Rio de Janeiro, São Paulo, Santa Catarina, Sergipe), Colombia, Costa Rica, Cuba, Ecuador, French Guiana, Galápagos Islands, Grenada, Panama, Paraguay, Peru, Puerto Rico, St. Vincent and the Grenadines, Suriname, Trinidad and Tobago, Uruguay, Venezuela (Moreira 2021e).

#### Notes

Previously recorded from Santarém (Moreira et al. 2011c); first record from Mojuí dos Campos.

#### Photographs

Fig. 17b, c

### Microvelia pulchella

Westwood, 1834

2B03F77F-08A9-574F-94C4-BF397B1021B8

Microvelia pulchella – see Westwood (1834): pl. VI, fig. 5.
Velia (Microvelia) pulchella – see Westwood (1834): 647.
Hydroessa pulchella – see Herrich-Schäffer (1842): 37, pl. CXCIII, fig. 595.
Microvelia pulchella – see Amyot and Audinet-Serville (1843): 422.
Microvelia capitata – see Guérin-Ménéville (1857): 417 (syn. by Drake and Hussey 1955: 104; Smith and Polhemus 1978: 65).
Rhagovelia incerta – see Kirby (1890): 548 (syn. by Polhemus and Chapman 1979: 53).
Microvelia robusta – see Uhler (1894): 219 (syn. by Drake and Maldonado-Capriles 1954: 219).
Microvelia marginata – see Uhler (1894): 219; Kirkaldy and Torre-Bueno (1909): 207; Torre-Bueno (1910): 150; Banks (1910): 27; Barber (1914): 500; Van Duzee (1917): 433; Hungerford (1920): 127 (partim; misidentification).
Microvelia boreale – see Torre-Bueno (1910): 150 (*nomen nudum*).
Microvelia borealis – see Torre-Bueno (1916): 59 (syn. by Drake and Hussey 1955: 104).

#### Materials

- **Type status:**Other material. **Occurrence:** recordedBy: S.E. Santos; sex: 8 macropterous ?, 4 macropterous ?; **Location:** country: Brazil; stateProvince: Pará; municipality: Mojuí dos Campos; locality: Igarapé Mojuí dos Caboclos; verbatimLatitude: 02°42'03.0"S; verbatimLongitude: 54°41'01.0"W; **Event:** verbatimEventDate: 02.X.2020; **Record Level:** type: PhysicalObject; institutionCode: LETIA; basisOfRecord: PreservedSpecimen
- **Type status:**Other material. **Occurrence:** recordedBy: S.E. Santos; sex: 1 macropterous ?, 3 macropterous ?; **Location:** country: Brazil; stateProvince: Pará; municipality: Mojuí dos Campos; locality: Igarapé Terra Preta; verbatimLatitude: 02°43'09.1"S; verbatimLongitude: 54°40'20.7"W; **Event:** verbatimEventDate: 10.II.2020; **Record Level:** type: PhysicalObject; institutionCode: LETIA; basisOfRecord: PreservedSpecimen
- **Type status:**Other material. **Occurrence:** recordedBy: S.E. Santos; sex: 1 macropterous ?, 1 macropterous ?; **Location:** country: Brazil; stateProvince: Pará; municipality: Mojuí dos Campos; locality: Igarapé Terra Preta; verbatimLatitude: 02°43'09.1"S; verbatimLongitude: 54°40'20.7"W; **Event:** verbatimEventDate: 24.II.2020; **Record Level:** type: PhysicalObject; institutionCode: LETIA; basisOfRecord: PreservedSpecimen
- **Type status:**Other material. **Occurrence:** recordedBy: S.E. Santos; sex: 1 macropterous ?, 1 macropterous ?; **Location:** country: Brazil; stateProvince: Pará; municipality: Santarém; locality: Igarapé Guaraná; verbatimLatitude: 02°46'25.9"S; verbatimLongitude: 54°23'20.6"W; **Event:** verbatimEventDate: 06.III.2020; **Record Level:** type: PhysicalObject; institutionCode: LETIA; basisOfRecord: PreservedSpecimen
- **Type status:**Other material. **Occurrence:** recordedBy: S.E. Santos; sex: 2 apterous ?, 15 macropterous ?, 18 macropterous ?; **Location:** country: Brazil; stateProvince: Pará; municipality: Santarém; locality: Igarapé Jatobá; verbatimLatitude: 02°34'17.9"S; verbatimLongitude: 54°51'36.8"W; **Event:** verbatimEventDate: 10.X.2020; **Record Level:** type: PhysicalObject; institutionCode: LETIA; basisOfRecord: PreservedSpecimen
- **Type status:**Other material. **Occurrence:** recordedBy: S.E. Santos; sex: 39 macropterous ?, 34 macropterous ?; **Location:** country: Brazil; stateProvince: Pará; municipality: Santarém; verbatimLatitude: 02°27'32.6"S; verbatimLongitude: 54°44'48.4"W; **Event:** verbatimEventDate: 10.IV.2020; habitat: puddle; **Record Level:** type: PhysicalObject; institutionCode: LETIA; basisOfRecord: PreservedSpecimen
- **Type status:**Other material. **Occurrence:** recordedBy: E.C. Oliveira; sex: 1 apterous ?; **Location:** country: Brazil; stateProvince: Pará; municipality: Santarém; locality: Lago Mapiri; verbatimLatitude: 02°25'28.5"S; verbatimLongitude: 54°44'47.7"W; **Event:** verbatimEventDate: 18.II.2020; **Record Level:** type: PhysicalObject; institutionCode: LETIA; basisOfRecord: PreservedSpecimen

#### Distribution

Alaska, Anguilla, Argentina, Aruba, Bahamas, Barbados, Bonaire, Brazil (Alagoas, Amazonas, Bahia, Espírito Santo, Maranhão, Mato Grosso do Sul, Minas Gerais, Pará, Pernambuco, Piauí, Rio de Janeiro, Santa Catarina, São Paulo, Sergipe), Canada, Cayman Islands, Colombia, Costa Rica, Cuba, Curaçao, Dominican Republic, Ecuador, French Guiana, Grenada, Guadeloupe, Guatemala, Jamaica, Klein Bonaire, Klein Curaçao, Martinique, Mexico, Panama, Peru, Puerto Rico, Saba, St. Kitts and Nevis, St. Martin, St. Vincent and the Grenadines, Trinidad and Tobago, United States, U.S. Virgin Islands, Venezuela (Moreira 2021e).

#### Notes

First records from the study area.

#### Photographs

Fig. 17d, e, f

### Microvelia sousorum sp. n.

62360FD2-B11E-51EA-834E-7993A60D5822

FE5976C2-FFE8-473E-84C5-484415A7BE3B

#### Materials

- **Type status:**Holotype. **Occurrence:** recordedBy: S.E. Santos; sex: apterous ?; **Location:** country: Brazil; stateProvince: Pará; municipality: Mojuí dos Campos; locality: Igarapé Água Fria; verbatimLatitude: 02°47'19.7"S; verbatimLongitude: 54°38'40.9"W; **Event:** verbatimEventDate: 24.IX.2020; **Record Level:** type: PhysicalObject; institutionCode: CEIOC 81765; basisOfRecord: PreservedSpecimen
- **Type status:**Paratype. **Occurrence:** recordedBy: S.E. Santos; sex: 1 apterous ?; **Location:** country: Brazil; stateProvince: Pará; municipality: Mojuí dos Campos; locality: Igarapé Água Fria; verbatimLatitude: 02°47'19.7"S; verbatimLongitude: 54°38'40.9"W; **Event:** verbatimEventDate: 24.IX.2020; **Record Level:** type: PhysicalObject; institutionCode: CEIOC 81766; basisOfRecord: PreservedSpecimen

#### Description

##### Apterous male

BL 1.62, HL 0.32, HW 0,44, ANT I 0.21, ANT II 0.13, ANT III 0.28, ANT IV 0.42, INT 0.25, EYE 0.09, PL 0.23, PW 0.46; FORE LEG: FEM 0.46, TIB 0.34, TAR I 0.18; MID-LEG: FEM 0.54, TIB 0.38, TAR I 0.08, TAR II 0.12; HIND LEG: FEM 0.57, TIB 0.60, TAR I 0.09, TAR II 0.10.

**Figure 18.**
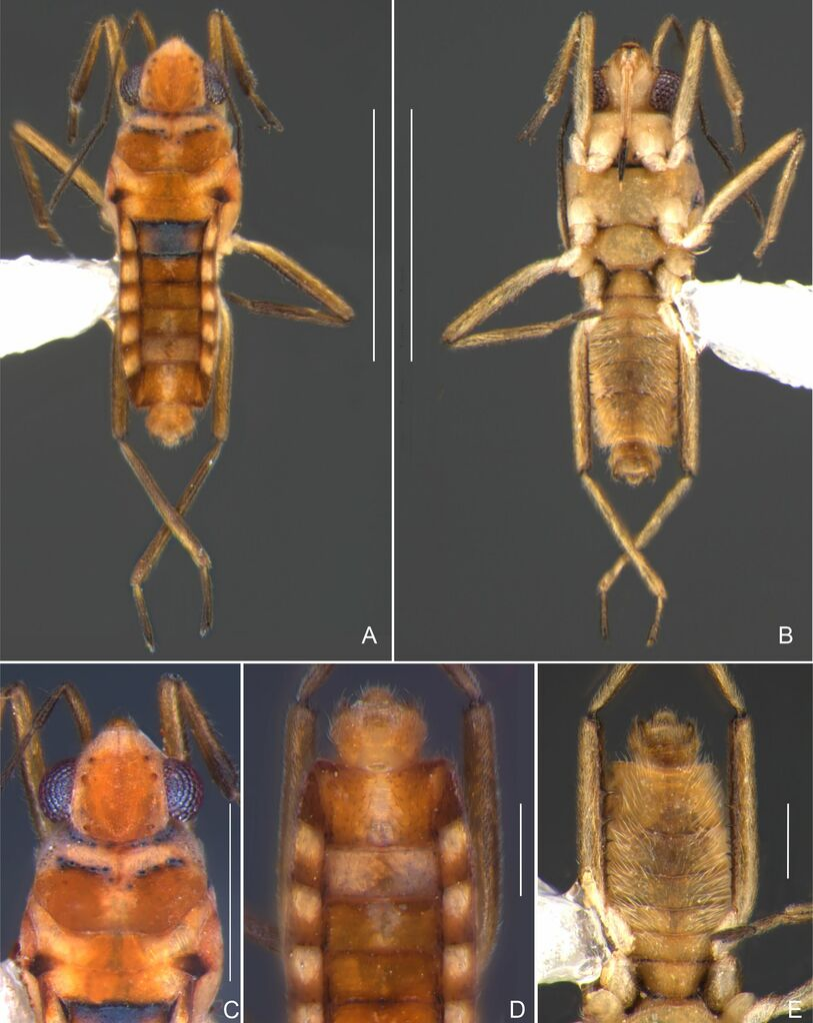
*Microveliasousorum***sp. nov.**, apterous male, holotype **A.** Habitus, dorsal view; **B.** Habitus, ventral view; **C.** Head and thorax, dorsal view; **D.** Abdomen, segments III–VII and terminalia, dorsal view; **E.** Abdomen and hind femora, ventral view. Scale bars: (A and B) 1 mm, (C) 0.5 mm, (D and E) 0.2 mm.

Head dorsally yellowish-brown, lighter on sides and anterior third, dark-brown on insertion of trichobothria; ventrally pale-yellow. Antenna dark-brown, except antennomere I yellowish-brown. Eye reddish. Labium yellowish-brown, except apex of article III and entire IV dark-brown. Pronotum yellowish-brown, lighter on transverse band on anterior third and at middle of posterior two-thirds, dark-brown around punctures adjacent to anterior margin and between anterior and posterior lobes (Fig. 18A, C). Metanotum pale-yellow, darker centrally. Ventre of thorax pale-yellow, anterior margins of meso- and metasterna dark-brown. Acetabula, coxae, trochanters and ventre of femora pale-yellow; dorsum of femora pale-yellow basally, dark-yellow towards apex; fore tibia yellowish-brown, dark-brown on apical third; middle and hind tibiae brown, lighter ventrally on proximal half; tarsi brown. Abdominal mediotergite I yellowish-brown, pale yellow on sides; II dark-brown, yellowish-brown on sides and anterior margin; III yellowish-brown, anterior margin dark brown, small pale-yellow patch at middle; IV yellowish-brown; V and VII yellowish-brown, each with longitudinal light-yellow band at middle; VI light-yellow. Abdominal laterotergite II pale-yellow, III–VI yellowish-brown on anterior half, pale-yellow on posterior half; VII yellowish-brown. Sides of abdomen yellow, dark-brown around opening of scent glands and on anterior margins of segments II–III. Ventre of abdomen yellow, dark-brown on sides of segments I–II (Fig. 18B). Terminalia yellowish-brown.

Head with silvery pubescence adjacent to mesal margins of eyes and on posterior third; impressed median line inconspicuous; clypeus with long setae. Antenna long, reaching abdominal segment I; covered with short brown setae, with longer, lighter setae on antennomere IV. Antennomere I widest, slightly curved laterally, thickened towards apex; II wider than III–IV, thickened towards apex; III cylindrical, thinner than IV; IV fusiform, at middle subequal to II in thickness. Labium reaching middle of mesosternum.

Pronotum completely covering mesonotum, but not metanotum (Fig. 18C); anterolateral angles rounded; anterior third covered with silvery pubescence; circular punctures on a transverse row adjacent to anterior margin; another centrally interrupted transverse row posterior to yellowish band; two submedian punctures posteriorly displaced from latter row; posterior margin slightly concave at middle. Metanotum almost 1/3 as long as pronotum, unpunctured; posterior margin rounded. Propleuron with dense, short, light setae, many small punctures and a posterior row of larger punctures; meso- and metapleuron unpunctured, without depressions. Ventre of thorax without elaborated ornamentation, except two dark punctures on both pro- and mesosternum near coxae. Legs covered with short brown setae, with some longer setae on dorsal surfaces of femora and tibiae. Fore tibia straight, slightly thicker on apex than on base, without apical spine. Hind femur slightly thicker than middle femur, ventrally with a row of distally increasing spines on proximal two-thirds (Fig. 18E). Hind tibia straight.

**Figure 19.**
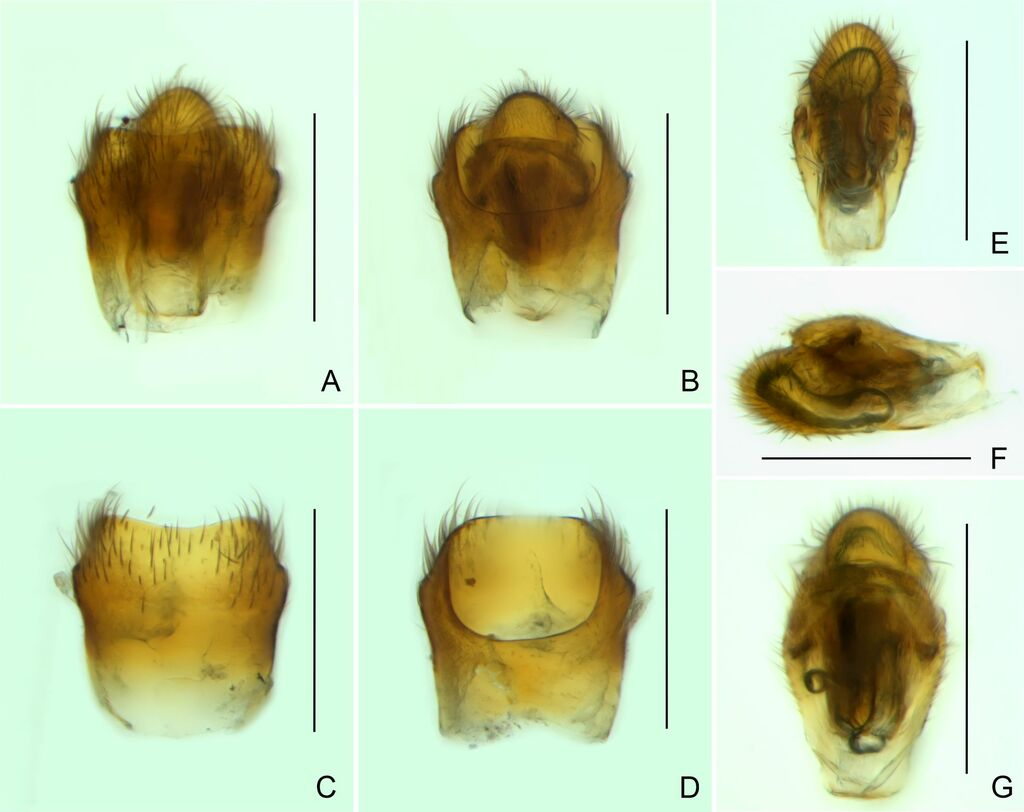
*Microveliasousorum***sp. nov.**, male terminalia **A.** Dorsal view **B.** Ventral view **C, D** Abdominal segment VIII: **C.** Dorsal view; **D.** Ventral view; **E–G** Genital capsule: **E.** Dorsal view; **F.** Lateral view; **G.** Ventral view. Scale bars: 0.2 mm.

Abdominal mediotergites covered with short setae. Mediotergite I shorter than II, with posterior margin slightly concave centrally; II with silvery pubescence medially; IV–VII with median line depressed; posterior margins of II–VI straight or almost straight; VII slightly concave (Fig. 18D). Abdominal sterna IV–VII depressed medially, with two longitudinal bands of dense, long setae laterally; posterior margin of VII slightly concave (Fig. 18E). Terminalia (Fig. 19) well-exposed; abdominal segment VIII dorsally with about two-thirds the length of mediotergite VII; posterior margin concave dorsally (Fig. 19C), ventrally curved, not notched (Fig. 19D). Proctiger exposed dorsally only by apex (Fig. 19A); posterior margin rounded (Fig. 19E). Pygophore convex, posterior margin rounded; parameres symmetrical (Fig. 19G).

##### Apterous female

BL 1.73, HL 0.35, HW 0.47, ANT I 0.19, ANT II 0.12, ANT III 0.26, ANT IV 0.40, INT 0.28, EYE 0.09, PL 0.26, PW 0.56; FORE LEG: FEM 0.46, TIB 0.33, TAR I 0.18; MID-LEG: FEM 0.48, TIB 0.37, TAR I 0.07, TAR II 0.13; HIND LEG: FEM 0.54, TIB 0.56, TAR I 0.10, TAR II 0.14.

**Figure 20.**
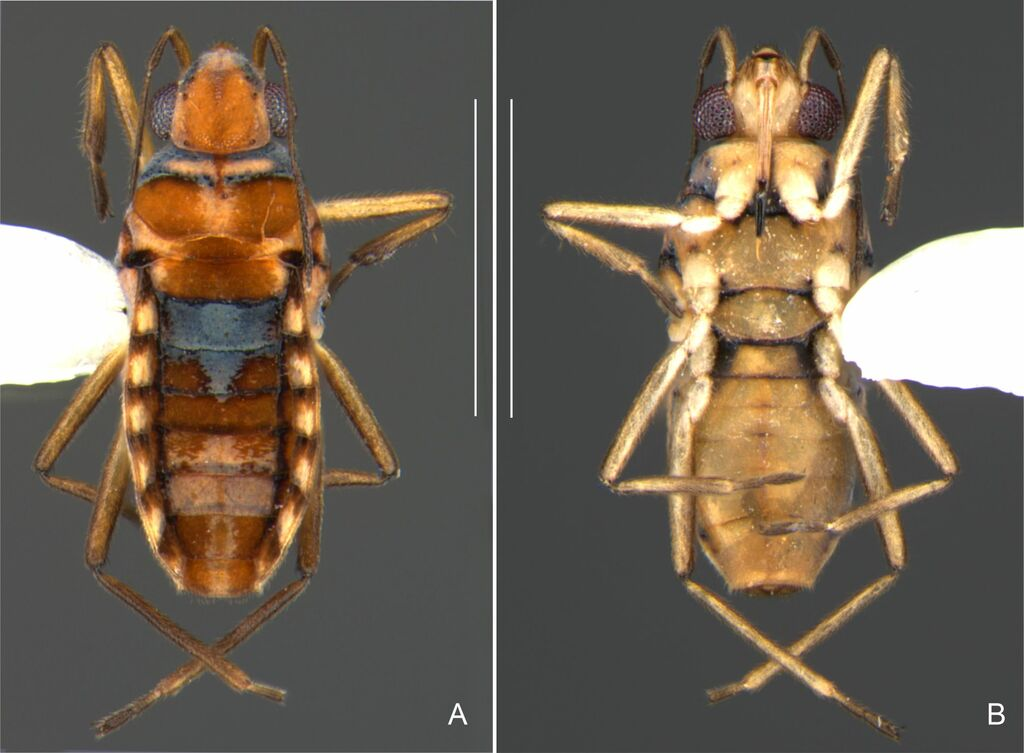
*Microveliasousorum***sp. nov.**, apterous female paratype, habitus **A.** Dorsal view; **B.** Ventral view. Scale bars: 1 mm.

Colouration and structure similar to apterous male, but larger and more robust (Fig. 20). Head dorsally with both sides of the anterior half black. Pronotum with anterior lobe black, except for a transverse pale-yellow band at middle and a straight yellowish-brown longitudinal band. Ventre of thorax pale-yellow, dark at prosternal groove and posterior margin of prosternum. Hind femur without spines. Abdominal mediotergite I with posterior margin straight; II dark-brown, with silvery pubescence; III yellowish-brown, anterior margin and middle patch black with silvery pubescence; VIII yellowish-brown with median pale-yellow band. Mediotergites V–VIII with median line depressed, with small transverse grooves. All abdominal laterotergites with anterior half dark-brown and posterior half pale-yellow. Abdominal laterotergites elevated to about 90°. Posterior margin of abdominal mediotergite VIII slightly rounded, with long setae. Abdominal sterna pale-yellow, covered with short setae, with straight median stripe slightly darker on segments IV–VII, sides with a dark-brown longitudinal stripe; silvery pubescence on dark stripe of sterna V–VI. Sterna V–VII slightly depressed at middle.

#### Diagnosis

This new species can be grouped with other South American *Microvelia* in which the pronotum completely or almost completely covers the mesonotum, but not the metanotum and the terminalia is well-exposed, not deeply inserted into the pregenital abdomen: *M.chilena* Drake & Hussey, 1955, *M.costaiana* Drake & Hussey, 1951, *M.nessimiani* Moreira & Rúdio, 2011, *M.mimula* White, 1879, *M.novana* Drake & Plaumann, 1955, *M.quieta* Drake & Carvalho, 1954 and *M.sarpta* Drake & Harris, 1936.

**Figure 21.**
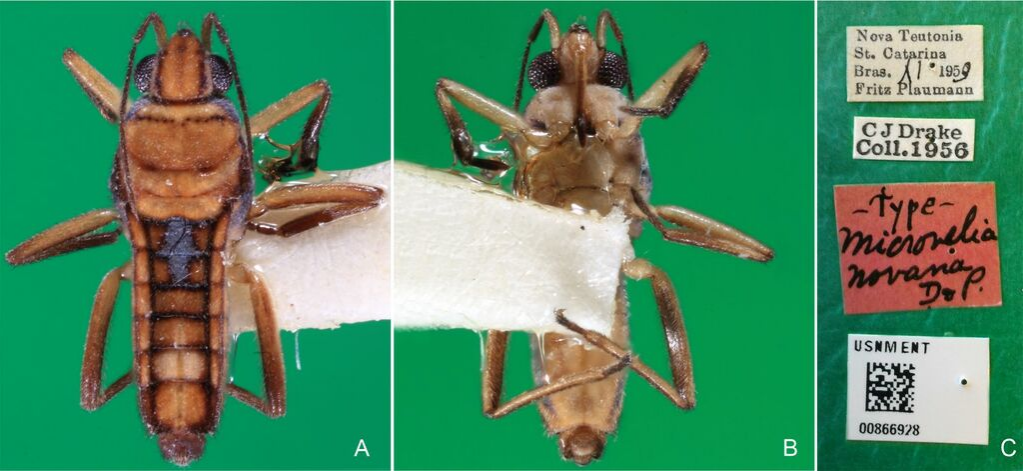
*Microvelianovana*, apterous male holotype deposited in the Entomology Collection of the Smithsonian National Museum of Natural History (NMNH) **A.** Habitus, dorsal view; **B.** Habitus, ventral view; **C.** Labels.

**Figure 22.**
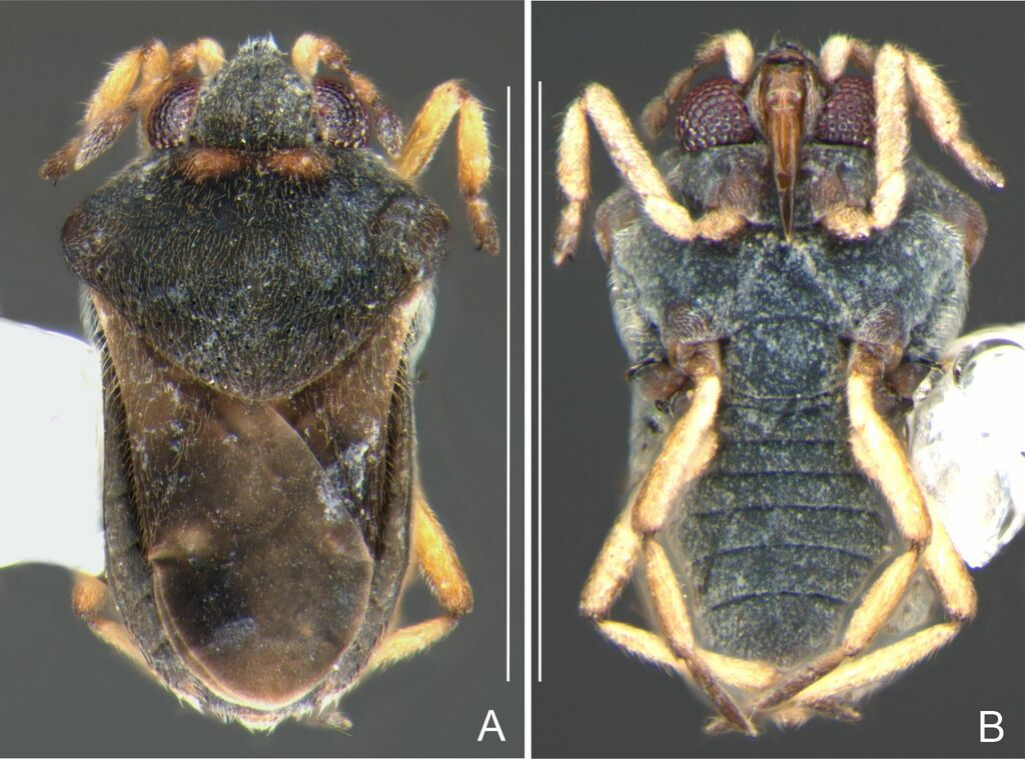
*Microveliavenustatis*, macropterous female, habitus **A.** Dorsal view; **B.** Ventral view. Scale bars: 1 mm.

*Microveliasousorum***sp. nov.** is most similar to *M.novana* (Fig. 21), known only from the type specimens from Santa Catarina State, southern Brazil. They can be separated by the shapes of the posterior margins of the pronotum (slightly concave at the middle in *M.sousorum* **sp. nov.** vs. rounded in *M.novana*), male abdominal mediotergite VII (slightly concave at the middle vs. straight) and male abdominal sternum VIII (straight vs. concave) and by the presence of spines on the hind femur of males in the new species, which are absent in *M.novana*.

#### Etymology

This species is named in honour of Carlos Sousa and Diego Sousa, colleagues who were both instrumental in our fieldwork. In addition, Carlos is SES’ husband and gave all the necessary support for her to obtain her Master’s degree, which resulted in this paper.

### Microvelia summersi

33F2D4A5-C547-55E4-B348-26C87625A6FE

Microvelia summersi – see Drake and Harris (1928): 8.

#### Distribution

Brazil (Amazonas, Pará), Grenada, Guyana, Panama, Trinidad and Tobago (Moreira 2021e).

#### Notes

Previously recorded from Santarém (Moreira et al. 2011a), but absent from our samples.

### Microvelia venustatis

Drake & Harris, 1933

57F59BA0-4E83-5B67-B4B0-625ED1D70B02

Microvelia venustatis – see Drake and Harris (1933): 53.

#### Materials

- **Type status:**Other material. **Occurrence:** recordedBy: S.E. Santos; sex: 1 macropterous ?; **Location:** country: Brazil; stateProvince: Pará; municipality: Santarém; verbatimLatitude: 02°27'32"S; verbatimLongitude: 54°44'48"W; **Event:** verbatimEventDate: 14.IV.2020; habitat: puddle; **Record Level:** type: PhysicalObject; institutionCode: LETIA; basisOfRecord: PreservedSpecimen

#### Distribution

Argentina, Brazil (Alagoas, Amazonas, Espírito Santo, Maranhão, Mato Grosso, Minas Gerais, Pará, Rio de Janeiro, São Paulo, Santa Catarina, Sergipe), Colombia, Paraguay, Peru (Moreira 2021e).

#### Notes

Previously recorded from Santarém (Moreira et al. 2011a).

#### Photographs

Fig. 22

### Rhagovelia amazonensis

Gould, 1931

65C7118E-4021-5512-8087-659C629437DE

Rhagovelia amazonensis – see Gould (1931): 15, pl. IV, fig. 2.

#### Distribution

Brazil (Amazonas, Mato Grosso, Pará, Rondônia), Guyana (Moreira 2021e).

#### Notes

Previously recorded from Santarém (Polhemus 1997), but absent from our samples.

### Rhagovelia brunae

Magalhães & Moreira, 2016

81F1B35A-0BF6-5AB0-97C6-EC33D2E6A3F2

Rhagovelia amazonensis – see Cunha et al. (2015): 427 (misidentification).
Rhagovelia brunae – see Magalhães & Moreira in Magalhães et al. (2016): 587, figs. 1–8 and 11.

#### Materials

- **Type status:**Other material. **Occurrence:** recordedBy: S.E. Santos; sex: 2 apterous ?, 34 apterous ?, 1 macropterous ?; **Location:** country: Brazil; stateProvince: Pará; municipality: Belterra; locality: Igarapé Jatuarana; verbatimLatitude: 03°15'44.7"S; verbatimLongitude: 54°56'37.5"W; **Event:** verbatimEventDate: 11.II.2020; **Record Level:** type: PhysicalObject; institutionCode: LETIA; basisOfRecord: PreservedSpecimen
- **Type status:**Other material. **Occurrence:** recordedBy: S.E. Santos; sex: 7 apterous ?, 20 apterous ?; **Location:** country: Brazil; stateProvince: Pará; municipality: Belterra; locality: Igarapé Coronel Batista; verbatimLatitude: 02°37'50.6"S; verbatimLongitude: 54°58'12.4"W; **Event:** verbatimEventDate: 08.XI.2019; **Record Level:** type: PhysicalObject; institutionCode: LETIA; basisOfRecord: PreservedSpecimen
- **Type status:**Other material. **Occurrence:** recordedBy: S.E. Santos; sex: 10 apterous ?, 9 apterous ?, 1 macropterous ?; **Location:** country: Brazil; stateProvince: Pará; municipality: Mojuí dos Campos; locality: Igarapé Água Fria; verbatimLatitude: 02°47'19.7"S; verbatimLongitude: 54°38'40.9"W; **Event:** verbatimEventDate: 24.IX.2020; **Record Level:** type: PhysicalObject; institutionCode: LETIA; basisOfRecord: PreservedSpecimen
- **Type status:**Other material. **Occurrence:** recordedBy: S.E. Santos; sex: 23 apterous ?, 24 apterous ?; **Location:** country: Brazil; stateProvince: Pará; municipality: Mojuí dos Campos; locality: Igarapé do Manel; verbatimLatitude: 02°25'06.3"S; verbatimLongitude: 54°44'26.3"W; **Event:** verbatimEventDate: 24.II.2020; **Record Level:** type: PhysicalObject; institutionCode: LETIA; basisOfRecord: PreservedSpecimen
- **Type status:**Other material. **Occurrence:** recordedBy: S.E. Santos; sex: 5 apterous ?, 5 apterous ?; **Location:** country: Brazil; stateProvince: Pará; municipality: Mojuí dos Campos; locality: Igarapé Terra Preta; verbatimLatitude: 02°43'09.1"S; verbatimLongitude: 54°40'20.7"W; **Event:** verbatimEventDate: 10.II.2020; **Record Level:** type: PhysicalObject; institutionCode: LETIA; basisOfRecord: PreservedSpecimen
- **Type status:**Other material. **Occurrence:** recordedBy: S.E. Santos; sex: 2 apterous ?; **Location:** country: Brazil; stateProvince: Pará; municipality: Mojuí dos Campos; locality: Igarapé Santa Júlia; verbatimLatitude: 02°40'19.7"S; verbatimLongitude: 54°43'06.9"W; **Event:** verbatimEventDate: 09.XII.2019; **Record Level:** type: PhysicalObject; institutionCode: LETIA; basisOfRecord: PreservedSpecimen
- **Type status:**Other material. **Occurrence:** recordedBy: S.E. Santos; sex: 1 apterous ?, 1 apterous ?; **Location:** country: Brazil; stateProvince: Pará; municipality: Santarém; locality: Igarapé Diamantino; verbatimLatitude: 02°30'16.2"S; verbatimLongitude: 54°39'32.9"W; **Event:** verbatimEventDate: 06.IX.2019; **Record Level:** type: PhysicalObject; institutionCode: LETIA; basisOfRecord: PreservedSpecimen
- **Type status:**Other material. **Occurrence:** recordedBy: S.E. Santos; sex: 5 apterous ?; **Location:** country: Brazil; stateProvince: Pará; municipality: Santarém; locality: Igarapé Diamantino; verbatimLatitude: 02°30'16.2"S; verbatimLongitude: 54°39'32.9"W; **Event:** verbatimEventDate: 16.IX.2019; **Record Level:** type: PhysicalObject; institutionCode: LETIA; basisOfRecord: PreservedSpecimen
- **Type status:**Other material. **Occurrence:** recordedBy: S.E. Santos; sex: 1 apterous ?, 5 apterous ?, 1 macropterous ?; **Location:** country: Brazil; stateProvince: Pará; municipality: Santarém; locality: Igarapé São Braz; verbatimLatitude: 02°29'07.0"S; verbatimLongitude: 54°49'41.9"W; **Event:** verbatimEventDate: 26.VIII.2019; **Record Level:** type: PhysicalObject; institutionCode: LETIA; basisOfRecord: PreservedSpecimen
- **Type status:**Other material. **Occurrence:** recordedBy: S.E. Santos; sex: 8 apterous ?, 4 apterous ?; **Location:** country: Brazil; stateProvince: Pará; municipality: Santarém; locality: Cachoeira da Cavada; verbatimLatitude: 02°35'48.9"S; verbatimLongitude: 54°31'47.3"W; **Event:** verbatimEventDate: 13.XI.2019; **Record Level:** type: PhysicalObject; institutionCode: LETIA; basisOfRecord: PreservedSpecimen
- **Type status:**Other material. **Occurrence:** recordedBy: S.E. Santos; sex: 6 apterous ?, 10 apterous ?, 2 macropterous ?, 1 macropterous ?; **Location:** country: Brazil; stateProvince: Pará; municipality: Santarém; locality: Igarapé Mararú; verbatimLatitude: 02°29'35.9"S; verbatimLongitude: 54°40'06.6"W; **Event:** verbatimEventDate: 23.VIII.2019; **Record Level:** type: PhysicalObject; institutionCode: LETIA; basisOfRecord: PreservedSpecimen
- **Type status:**Other material. **Occurrence:** recordedBy: S.E. Santos; sex: 1 apterous ?, 2 apterous ?; **Location:** country: Brazil; stateProvince: Pará; municipality: Santarém; locality: Igarapé do Rai; verbatimLatitude: 02°35'35.3"S; verbatimLongitude: 54°30'18.1"W; **Event:** verbatimEventDate: 13.XI.2019; **Record Level:** type: PhysicalObject; institutionCode: LETIA; basisOfRecord: PreservedSpecimen
- **Type status:**Other material. **Occurrence:** recordedBy: S.E. Santos; sex: 22 apterous ?, 21 apterous ?; **Location:** country: Brazil; stateProvince: Pará; municipality: Santarém; locality: Igarapé Cajutuba II; verbatimLatitude: 02°27'39.2"S; verbatimLongitude: 54°46'53.4"W; **Event:** verbatimEventDate: 10.X.2020; **Record Level:** type: PhysicalObject; institutionCode: LETIA; basisOfRecord: PreservedSpecimen
- **Type status:**Other material. **Occurrence:** recordedBy: M. Galúcio; sex: 1 apterous ?, 4 apterous ?; **Location:** country: Brazil; stateProvince: Pará; municipality: Santarém; locality: Igarapé Mutunuy; verbatimLatitude: 02°28'53.1"S; verbatimLongitude: 54°41'45.9"W; **Event:** verbatimEventDate: 17.X.2015; **Record Level:** type: PhysicalObject; institutionCode: LETIA; basisOfRecord: PreservedSpecimen
- **Type status:**Other material. **Occurrence:** recordedBy: M. Galúcio; sex: 1 apterous ?; **Location:** country: Brazil; stateProvince: Pará; municipality: Santarém; locality: Igarapé Mutunuy; verbatimLatitude: 02°28'53.1"S; verbatimLongitude: 54°41'45.9"W; **Event:** verbatimEventDate: 29.X.2015; **Record Level:** type: PhysicalObject; institutionCode: LETIA; basisOfRecord: PreservedSpecimen

#### Distribution

Brazil (Alagoas, Maranhão, Pará, Sergipe), Venezuela (Moreira 2021e).

#### Notes

First records from the study area.

#### Photographs

Fig. 23a, b

### Rhagovelia elegans

Uhler, 1894 sp. n.

0292A586-C2E8-57BC-B88F-253A1AE41AB0

#### Materials

- **Type status:**Other material. **Occurrence:** recordedBy: S.E. Santos; sex: 3 apterous ?, 6 apterous ?; **Location:** country: Brazil; stateProvince: Pará; municipality: Belterra; locality: Igarapé do Ailton; verbatimLatitude: 02°35'36.7"S; verbatimLongitude: 54°57'48.4"W; **Event:** verbatimEventDate: 06.XI.2019; **Record Level:** type: PhysicalObject; institutionCode: LETIA; basisOfRecord: PreservedSpecimen
- **Type status:**Other material. **Occurrence:** recordedBy: S.E. Santos; sex: 2 apterous ?, 5 apterous ?; **Location:** country: Brazil; stateProvince: Pará; municipality: Belterra; locality: Igarapé Coronel Batista; verbatimLatitude: 02°37'50.6"S; verbatimLongitude: 54°58'12.4"W; **Event:** verbatimEventDate: 08.XI.2019; **Record Level:** type: PhysicalObject; institutionCode: LETIA; basisOfRecord: PreservedSpecimen
- **Type status:**Other material. **Occurrence:** recordedBy: S.E. Santos; sex: 1 apterous ?, 3 apterous ?; **Location:** country: Brazil; stateProvince: Pará; municipality: Belterra; locality: Floresta Nacional do Tapajós; verbatimLatitude: 03°03'02.6"S; verbatimLongitude: 54°55'30.1"W; **Event:** verbatimEventDate: 20.I.2020; habitat: igarapé; **Record Level:** type: PhysicalObject; institutionCode: LETIA; basisOfRecord: PreservedSpecimen
- **Type status:**Other material. **Occurrence:** recordedBy: S.E. Santos; sex: 3 apterous ?, 2 apterous ?; **Location:** country: Brazil; stateProvince: Pará; municipality: Mojuí dos Campos; locality: Igarapé Água Fria; verbatimLatitude: 02°47'19.7"S; verbatimLongitude: 54°38'40.9"W; **Event:** verbatimEventDate: 24.IX.2020; **Record Level:** type: PhysicalObject; institutionCode: LETIA; basisOfRecord: PreservedSpecimen
- **Type status:**Other material. **Occurrence:** recordedBy: S.E. Santos; sex: 2 apterous ?; **Location:** country: Brazil; stateProvince: Pará; municipality: Mojuí dos Campos; locality: Igarapé Antonio Leite; verbatimLatitude: 03°09'06.2"S; verbatimLongitude: 54°50'28.7"W; **Event:** verbatimEventDate: 18.X.2019; **Record Level:** type: PhysicalObject; institutionCode: LETIA; basisOfRecord: PreservedSpecimen
- **Type status:**Other material. **Occurrence:** recordedBy: S.E. Santos; sex: 2 macropterous ?; **Location:** country: Brazil; stateProvince: Pará; municipality: Santarém; locality: Igarapé São Braz; verbatimLatitude: 02°29'07.0"S; verbatimLongitude: 54°49'41.9"W; **Event:** verbatimEventDate: 26.VIII.2019; **Record Level:** type: PhysicalObject; institutionCode: LETIA; basisOfRecord: PreservedSpecimen
- **Type status:**Other material. **Occurrence:** recordedBy: S.E. Santos; sex: 7 apterous ?, 11 apterous ?; **Location:** country: Brazil; stateProvince: Pará; municipality: Santarém; locality: Cachoeira da Cavada; verbatimLatitude: 02°35'48.9"S; verbatimLongitude: 54°31'47.3"W; **Event:** verbatimEventDate: 13.XI.2019; **Record Level:** type: PhysicalObject; institutionCode: LETIA; basisOfRecord: PreservedSpecimen
- **Type status:**Other material. **Occurrence:** recordedBy: S.E. Santos; sex: 4 apterous ?, 5 apterous ?; **Location:** country: Brazil; stateProvince: Pará; municipality: Santarém; locality: Igarapé da Débora; verbatimLatitude: 02°44'27.7"S; verbatimLongitude: 54°26'01.2"W; **Event:** verbatimEventDate: 21.X.2019; **Record Level:** type: PhysicalObject; institutionCode: LETIA; basisOfRecord: PreservedSpecimen
- **Type status:**Other material. **Occurrence:** recordedBy: S.E. Santos; sex: 3 apterous ?, 8 apterous ?; **Location:** country: Brazil; stateProvince: Pará; municipality: Santarém; locality: Igarapé do Rai; verbatimLatitude: 02°35'35.3"S; verbatimLongitude: 54°30'18.1"W; **Event:** verbatimEventDate: 13.XI.2019; **Record Level:** type: PhysicalObject; institutionCode: LETIA; basisOfRecord: PreservedSpecimen
- **Type status:**Other material. **Occurrence:** recordedBy: S.E. Santos; sex: 7 apterous ?, 6 apterous ?; **Location:** country: Brazil; stateProvince: Pará; municipality: Santarém; locality: Igarapé Cajutuba II; verbatimLatitude: 02°27'39.2"S; verbatimLongitude: 54°46'53.4"W; **Event:** verbatimEventDate: 10.X.2020; **Record Level:** type: PhysicalObject; institutionCode: LETIA; basisOfRecord: PreservedSpecimen

#### Distribution

Brazil (Alagoas, Amapá, Amazonas, Espírito Santo, Mato Grosso, Pará, Rio de Janeiro, Sergipe), Colombia, Costa Rica, Dominica, Ecuador, Grenada, Hispaniola Island, Martinique, Panama, St. Kitts and Nevis, St. Lucia, St. Vincent and the Grenadines, Trinidad and Tobago, Venezuela (Moreira 2021e).

#### Notes

Previously recorded from Santarém (Bacon 1956, Polhemus 1997); first records from Belterra and Mojuí dos Campos.

#### Photograph

Fig. 23c

### Rhagovelia evidis

Bacon, 1948

F19726FA-F121-51D6-B6B8-CCBB9A016594

Rhagovelia evidis – see Bacon (1948): 73, fig. 6.

#### Materials

- **Type status:**Other material. **Occurrence:** recordedBy: S.E. Santos; sex: 1 apterous ?; **Location:** country: Brazil; stateProvince: Pará; municipality: Belterra; locality: Igarapé Jatuarana; verbatimLatitude: 03°15'44.7"S; verbatimLongitude: 54°56'37.5"W; **Event:** verbatimEventDate: 11.II.2020; **Record Level:** type: PhysicalObject; institutionCode: LETIA; basisOfRecord: PreservedSpecimen
- **Type status:**Other material. **Occurrence:** recordedBy: S.E. Santos; sex: 1 apterous ?; **Location:** country: Brazil; stateProvince: Pará; municipality: Mojuí dos Campos; locality: Igarapé Terra de Areia; verbatimLatitude: 02°47'58.7"S; verbatimLongitude: 54°38'15.6"W; **Event:** verbatimEventDate: 24.I.2020; **Record Level:** type: PhysicalObject; institutionCode: LETIA; basisOfRecord: PreservedSpecimen
- **Type status:**Other material. **Occurrence:** recordedBy: S.E. Santos; sex: 1 apterous ?; **Location:** country: Brazil; stateProvince: Pará; municipality: Santarém; locality: Igarapé Sonrizal; verbatimLatitude: 02°32'13.6"S; verbatimLongitude: 54°55'26.6"W; **Event:** verbatimEventDate: 09.VIII.2019; **Record Level:** type: PhysicalObject; institutionCode: LETIA; basisOfRecord: PreservedSpecimen

#### Distribution

Brazil (Amazonas, Pará), Peru (Moreira 2021e).

#### Notes

First records from the study area.

#### Photograph

Fig. 23d

### Rhagovelia graziae

Galindo-Malagón, Morales & Moreira, 2021

5C82D010-81F1-522B-8557-3F931C2D9C42

Rhagovelia graziae – see Galindo-Malagón et al. (2021): 198, figs. 9E, 10E, 11E, 12E, 19N, 20N and 23C.

#### Materials

- **Type status:**Other material. **Occurrence:** recordedBy: S.E. Santos; sex: 7 apterous ?, 6 apterous ?; **Location:** country: Brazil; stateProvince: Pará; municipality: Belterra; locality: Igarapé Aramanaí; verbatimLatitude: 02°42'56.8"S; verbatimLongitude: 54°59'59.3"W; **Event:** verbatimEventDate: 07.XI.2019; **Record Level:** type: PhysicalObject; institutionCode: LETIA; basisOfRecord: PreservedSpecimen

#### Distribution

Brazil (Pará), Colombia (Galindo-Malagón et al. 2021).

#### Notes

First record from Brazil.

#### Photograph

Fig. 23e

### Rhagovelia jubata

Bacon, 1948

293431DB-F573-5BAF-BD17-8D542F0C0761

Rhagovelia jubata – see Bacon (1948): 78, fig. 5.

#### Materials

- **Type status:**Other material. **Occurrence:** recordedBy: S.E. Santos; sex: 2 apterous ?; **Location:** country: Brazil; stateProvince: Pará; municipality: Belterra; locality: BR-163, Km-115; verbatimLatitude: 03°17'34.8"S; verbatimLongitude: 54°52'45.6"W; **Event:** verbatimEventDate: 23.XI.2019; habitat: igarapé; **Record Level:** type: PhysicalObject; institutionCode: LETIA; basisOfRecord: PreservedSpecimen
- **Type status:**Other material. **Occurrence:** recordedBy: S.E. Santos; sex: 1 apterous ?, 1 apterous ?; **Location:** country: Brazil; stateProvince: Pará; municipality: Belterra; locality: BR-163, Km-115; verbatimLatitude: 03°17'34.8"S; verbatimLongitude: 54°52'45.6"W; **Event:** verbatimEventDate: 25.IX.2020; **Record Level:** type: PhysicalObject; institutionCode: LETIA; basisOfRecord: PreservedSpecimen
- **Type status:**Other material. **Occurrence:** recordedBy: S.E. Santos; sex: 7 apterous ?, 2 apterous ?; **Location:** country: Brazil; stateProvince: Pará; municipality: Belterra; locality: Igarapé do Ailton; verbatimLatitude: 02°35'36.7"S; verbatimLongitude: 54°57'48.4"W; **Event:** verbatimEventDate: 06.XI.2019; **Record Level:** type: PhysicalObject; institutionCode: LETIA; basisOfRecord: PreservedSpecimen
- **Type status:**Other material. **Occurrence:** recordedBy: S.E. Santos; sex: 4 apterous ?, 5 apterous ?; **Location:** country: Brazil; stateProvince: Pará; municipality: Santarém; locality: Igarapé Sonrizal; verbatimLatitude: 02°32'13.6"S; verbatimLongitude: 54°55'26.6"W; **Event:** verbatimEventDate: 09.XI.2019; **Record Level:** type: PhysicalObject; institutionCode: LETIA; basisOfRecord: PreservedSpecimen
- **Type status:**Other material. **Occurrence:** recordedBy: S.E. Santos; sex: 86 apterous ?, 66 apterous ?; **Location:** country: Brazil; stateProvince: Pará; municipality: Santarém; locality: Cachoeira da Rocha Negra; verbatimLatitude: 02°29'48.5"S; verbatimLongitude: 54°45'13.3"W; **Event:** verbatimEventDate: 25.IX.2020; **Record Level:** type: PhysicalObject; institutionCode: LETIA; basisOfRecord: PreservedSpecimen
- **Type status:**Other material. **Occurrence:** recordedBy: S.E. Santos; sex: 2 apterous ?, 1 apterous ?; **Location:** country: Brazil; stateProvince: Pará; municipality: Santarém; locality: Igarapé da Débora; verbatimLatitude: 02°44'27.7"S; verbatimLongitude: 54°26'01.2"W; **Event:** verbatimEventDate: 21.X.2019; **Record Level:** type: PhysicalObject; institutionCode: LETIA; basisOfRecord: PreservedSpecimen
- **Type status:**Other material. **Occurrence:** recordedBy: M. Galúcio; sex: 1 apterous ?, 3 apterous ?; **Location:** country: Brazil; stateProvince: Pará; municipality: Santarém; locality: Igarapé Mutunuy; verbatimLatitude: 02°28'53.1"S; verbatimLongitude: 54°41'45.9"W; **Event:** verbatimEventDate: 17.X.2015; **Record Level:** type: PhysicalObject; institutionCode: LETIA; basisOfRecord: PreservedSpecimen

#### Distribution

Brazil (Amazonas, Pará, Rondônia), Ecuador, Peru (Moreira 2021e).

#### Notes

First records from the study area.

#### Photograph

Fig. 23f

### Rhagovelia tenuipes

Champion, 1898

4654693B-E913-5148-939F-2E911D847AF0

Rhagovelia tenuipes – see Champion (1898): 137, pl. VIII, fig. 29.
Rhagovelia gregalis – see *Drake and Harris (1927)*: 136 (syn. by Bacon 1956: 743).
Rhagovelia regalis – see *Drake and Harris (1927)*: 137 (syn. by Bacon 1956: 743).
Rhagovelia confusa – see Gould (1931): 23, pl. V, fig. 5 (syn. by Bacon 1956: 743).
Rhagovelia obscura – see Gould (1931): 38, pl. IV, fig. 6 (syn. by Bacon 1956: 743).
Rhagovelia vega – see Padilla-Gil (2011): 210, figs. 13–16 (syn. by Galindo-Malagón et al. 2021: 210).
Rhagovelia mocoa – see Padilla-Gil (2015): 88, figs. 14 and 41 (syn. by Galindo-Malagón et al. 2021: 210).
Rhagovelia umbria – see Padilla-Gil (2015): 90, figs. 15 and 42 (syn. by Galindo-Malagón et al. 2021: 210).

#### Distribution

Belize, Brazil (Alagoas, Amazonas, Espírito Santo, Maranhão, Mato Grosso, Mato Grosso do Sul, Minas Gerais, Pará, Roraima, Rio de Janeiro, São Paulo, Sergipe), Cayman Islands, Colombia, Costa Rica, Ecuador, Guatemala, Honduras, Mexico, Nicaragua, Peru, Trinidad and Tobago, Venezuela (Moreira 2021e).

#### Notes

Previously recorded from Santarém (Bacon 1956), but absent from our samples.

### Rhagovelia trailii

(White, 1879)

93F8F129-2BE9-53F8-8128-280CFA17A93F

Neovelia trailii – see White (1879a): 487.
Rhagovelia traili – see Kirkaldy and Torre-Bueno (1909): 206; Polhemus and Polhemus (1985): 168; Polhemus (1997): 174; Pereira and Melo (2007): 645; Moreira et al. (2011c): 26; Padilla-Gil and Moreira (2013): 415, 420, 423; Moreira and Barbosa (2014): 599; Cordeiro and Moreira (2015): 21; Floriano and Moreira (2015): 440; Magalhães et al. (2016): 591; Aristizábal-García (2017): 303, 897, [appendix] 210; Magalhães et al. (2019): 395 (incorrect subsequent spelling).
Rhagovelia trailii – see Gould (1931): 45; Bacon (1956): 878.
Rhagovelia perfidiosa – see Bacon (1948): 81, fig. 10 (syn. by Polhemus and Polhemus 1985: 168).

#### Materials

- **Type status:**Other material. **Occurrence:** recordedBy: S.E. Santos; sex: 16 apterous ?, 1 macropterous ?; **Location:** country: Brazil; stateProvince: Pará; municipality: Belterra; locality: Igarapé Jatuarana; verbatimLatitude: 03°15'44.7"S; verbatimLongitude: 54°56'37.5"W; **Event:** verbatimEventDate: 11.II.2020; **Record Level:** type: PhysicalObject; institutionCode: LETIA; basisOfRecord: PreservedSpecimen
- **Type status:**Other material. **Occurrence:** recordedBy: S.E. Santos; sex: 13 apterous ?, 10 apterous ?; **Location:** country: Brazil; stateProvince: Pará; municipality: Mojuí dos Campos; locality: Igarapé Terra de Areia; verbatimLatitude: 02°47'58.7"S; verbatimLongitude: 54°38'15.6"W; **Event:** verbatimEventDate: 24.I.2020; **Record Level:** type: PhysicalObject; institutionCode: LETIA; basisOfRecord: PreservedSpecimen
- **Type status:**Other material. **Occurrence:** recordedBy: S.E. Santos; sex: 5 apterous ?; **Location:** country: Brazil; stateProvince: Pará; municipality: Mojuí dos Campos; locality: Igarapé Santa Júlia; verbatimLatitude: 02°40'19.7"S; verbatimLongitude: 54°43'06.9"W; **Event:** verbatimEventDate: 09.XII.2019; **Record Level:** type: PhysicalObject; institutionCode: LETIA; basisOfRecord: PreservedSpecimen
- **Type status:**Other material. **Occurrence:** recordedBy: S.E. Santos; sex: 1 apterous ?, 2 apterous ?; **Location:** country: Brazil; stateProvince: Pará; municipality: Mojuí dos Campos; locality: Igarapé Antonio Leite; verbatimLatitude: 03°09'06.2"S; verbatimLongitude: 54°50'28.7"W; **Event:** verbatimEventDate: 18.X.2019; **Record Level:** type: PhysicalObject; institutionCode: LETIA; basisOfRecord: PreservedSpecimen
- **Type status:**Other material. **Occurrence:** recordedBy: S.E. Santos; sex: 3 apterous ?, 2 apterous ?; **Location:** country: Brazil; stateProvince: Pará; municipality: Mojuí dos Campos; locality: Igarapé Mojuí dos Caboclos; verbatimLatitude: 02°42'03.0"S; verbatimLongitude: 54°41'01.0"W; **Event:** verbatimEventDate: 21.I.2020; **Record Level:** type: PhysicalObject; institutionCode: LETIA; basisOfRecord: PreservedSpecimen
- **Type status:**Other material. **Occurrence:** recordedBy: S.E. Santos; sex: 2 apterous ?, 2 apterous ?; **Location:** country: Brazil; stateProvince: Pará; municipality: Mojuí dos Campos; locality: Igarapé Terra Preta; verbatimLatitude: 02°43'09.1"S; verbatimLongitude: 54°40'20.7"W; **Event:** verbatimEventDate: 24.II.2020; **Record Level:** type: PhysicalObject; institutionCode: LETIA; basisOfRecord: PreservedSpecimen
- **Type status:**Other material. **Occurrence:** recordedBy: S.E. Santos; sex: 21 apterous ?, 20 apterous ?; **Location:** country: Brazil; stateProvince: Pará; municipality: Santarém; locality: Igarapé Guaraná; verbatimLatitude: 02°46'25.9"S; verbatimLongitude: 54°23'20.6"W; **Event:** verbatimEventDate: 06.III.2020; **Record Level:** type: PhysicalObject; institutionCode: LETIA; basisOfRecord: PreservedSpecimen
- **Type status:**Other material. **Occurrence:** recordedBy: S.E. Santos; sex: 3 apterous ?, 2 apterous ?; **Location:** country: Brazil; stateProvince: Pará; municipality: Santarém; locality: Ponte do Juá; verbatimLatitude: 02°26'40.6"S; verbatimLongitude: 54°47'21.1"W; **Event:** verbatimEventDate: 06.XII.2019; **Record Level:** type: PhysicalObject; institutionCode: LETIA; basisOfRecord: PreservedSpecimen
- **Type status:**Other material. **Occurrence:** recordedBy: S.E. Santos; sex: 1 macropterous ?; **Location:** country: Brazil; stateProvince: Pará; municipality: Santarém; locality: Igarapé Diamantino; verbatimLatitude: 02°30'16.2"S; verbatimLongitude: 54°39'32.9’’W; **Event:** verbatimEventDate: 06.IX.2019; **Record Level:** type: PhysicalObject; institutionCode: LETIA; basisOfRecord: PreservedSpecimen
- **Type status:**Other material. **Occurrence:** recordedBy: S.E. Santos; sex: 3 macropterous ?; **Location:** country: Brazil; stateProvince: Pará; municipality: Santarém; locality: Igarapé Diamantino; verbatimLatitude: 02°30'16.2"S; verbatimLongitude: 54°39'32.9"W; **Event:** verbatimEventDate: 16.IX.2019; **Record Level:** type: PhysicalObject; institutionCode: LETIA; basisOfRecord: PreservedSpecimen
- **Type status:**Other material. **Occurrence:** recordedBy: S.E. Santos; sex: 2 apterous ?, 6 apterous ?, 1 macropterous ?; **Location:** country: Brazil; stateProvince: Pará; municipality: Santarém; locality: Igarapé São Braz; verbatimLatitude: 02°29'07.0"S; verbatimLongitude: 54°49'41.9"W; **Event:** verbatimEventDate: 26.VIII.2019; **Record Level:** type: PhysicalObject; institutionCode: LETIA; basisOfRecord: PreservedSpecimen
- **Type status:**Other material. **Occurrence:** recordedBy: S.E. Santos; sex: 3 apterous ?, 2 apterous ?; **Location:** country: Brazil; stateProvince: Pará; municipality: Santarém; locality: Igarapé Jatobá; verbatimLatitude: 02°34'17.9"S; verbatimLongitude: 54°51'36.8"W; **Event:** verbatimEventDate: 10.X.2020; **Record Level:** type: PhysicalObject; institutionCode: LETIA; basisOfRecord: PreservedSpecimen
- **Type status:**Other material. **Occurrence:** recordedBy: M. Galúcio; sex: 1 apterous ?; **Location:** country: Brazil; stateProvince: Pará; municipality: Santarém; locality: Igarapé Mutunuy; verbatimLatitude: 02°28'53.1"S; verbatimLongitude: 54°41'45.9"W; **Event:** verbatimEventDate: 29.X.2015; **Record Level:** type: PhysicalObject; institutionCode: LETIA; basisOfRecord: PreservedSpecimen

#### Distribution

Brazil (Amazonas, Pará, Roraima), French Guiana, Peru, Suriname, Venezuela (Moreira 2021e).

#### Notes

Previously recorded from Santarém (Bacon 1948; as *R.perfidiosa*); first records from Belterra and Mojuí dos Campos.

#### Photographs

Fig. 24

### Callivelia conata

(Hungerford, 1929)

4995BA73-50B7-5993-8DAE-674C9A12B959

Velia conata – see Hungerford (1929a): 199.
Paravelia conata – see Polhemus (1976): 509.
Callivelia conata – see Polhemus (2021): 349.

#### Materials

- **Type status:**Other material. **Occurrence:** recordedBy: S.E. Santos; sex: 1 macropterous ?; **Location:** country: Brazil; stateProvince: Pará; municipality: Santarém; locality: Cachoeira da Rocha Negra; verbatimLatitude: 02°29'48.5"S; verbatimLongitude: 54°45'13.3"W; **Event:** verbatimEventDate: 25.IX.2020; **Record Level:** type: PhysicalObject; institutionCode: LETIA; basisOfRecord: PreservedSpecimen

#### Distribution

Brazil (Alagoas, Amazonas, Espírito Santo, Goiás, Mato Grosso, Pará, Rondônia), French Guiana, Guyana, Peru, Suriname, Trinidad and Tobago, Venezuela (Moreira 2021e).

#### Notes

First record from the study area.

#### Photograph

Fig. 25a

### Oiovelia chenae

Rodrigues & Melo, 2014

F1711266-266C-551C-802D-E4009FFC06C7

Oiovelia chenae – see Rodrigues & Melo in Rodrigues et al. (2014a): 84, figs. 47–49, 59–61, 74 and 78.

#### Distribution

Brazil (Amazonas, Pará) (Moreira 2021e).

#### Notes

Previously recorded from Santarém (Rodrigues et al. 2014a), but absent from our samples.

### Oiovelia cunucunumana

Drake & Maldonado-Capriles, 1952

F4E8357A-1259-5CF5-B64A-4A0F5364A23C

Oiovelia cunucunumana – see Drake and Maldonado-Capriles (1952): 52, fig. 1.
Paravelia correntina – see Iglesias and Crespo (1999): 259, figs. 1–11 (syn. by Torres et al. 2007: 143).

#### Materials

- **Type status:**Other material. **Occurrence:** recordedBy: S.E. Santos; sex: 23 macropterous ?, 12 macropterous ?; **Location:** country: Brazil; stateProvince: Pará; municipality: Mojuí dos Campos; locality: Igarapé Antonio Leite; verbatimLatitude: 03°09'06.2"S; verbatimLongitude: 54°50'28.7"W; **Event:** verbatimEventDate: 18.X.2019; **Record Level:** type: PhysicalObject; institutionCode: LETIA; basisOfRecord: PreservedSpecimen

#### Distribution

Argentina, Brazil (Amapá, Amazonas, Bahia, Minas Gerais, Pará, São Paulo, Santa Catarina), Colombia, Peru, Paraguay, Venezuela (Moreira 2021e).

#### Notes

First record from the study area.

#### Photograph

Fig. 25b

### Paravelia bullialata

Polhemus & Polhemus, 1984

EBDE7892-5451-5DCA-B6CF-C9069298C4CE

Paravelia bullialata – see Polhemus and Polhemus (1984): 342, figs. 4 and 5a.

#### Materials

- **Type status:**Other material. **Occurrence:** recordedBy: S.E. Santos; sex: 2 macropterous ?, 1 macropterous ?; **Location:** country: Brazil; stateProvince: Pará; municipality: Belterra; locality: Floresta Nacional do Tapajós; verbatimLatitude: 03°03'02.6"S; verbatimLongitude: 54°55'30.1"W; **Event:** verbatimEventDate: 20.I.2020; habitat: igarapé; **Record Level:** type: PhysicalObject; institutionCode: LETIA; basisOfRecord: PreservedSpecimen
- **Type status:**Other material. **Occurrence:** recordedBy: S.E. Santos; sex: 1 macropterous ?; **Location:** country: Brazil; stateProvince: Pará; municipality: Belterra; locality: BR-163, Km-115; verbatimLatitude: 03°17'34.8"S; verbatimLongitude: 54°52'45.6"W; **Event:** verbatimEventDate: 23.XI.2019; habitat: igarapé; **Record Level:** type: PhysicalObject; institutionCode: LETIA; basisOfRecord: PreservedSpecimen

#### Distribution

Bolivia, Brazil (Amazonas, Pará, Rondônia), French Guiana, Guyana, Suriname, Venezuela (Moreira 2021e)

#### Notes

First records from the study area.

#### Photograph

Fig. 25c

### Paravelia dilatata

Polhemus & Polhemus, 1984

BEC45209-6E57-5233-A1AB-47C4236388AD

Paravelia dilatata – see Polhemus and Polhemus (1984): 498, figs. 1 and 3.

#### Materials

- **Type status:**Other material. **Occurrence:** recordedBy: M. Galúcio; sex: 1 macropterous ?; **Location:** country: Brazil; stateProvince: Pará; municipality: Santarém; locality: Igarapé Mutunuy; verbatimLatitude: 02°28'53.1"S; verbatimLongitude: 54°41'45.9"W; **Event:** verbatimEventDate: 17.X.2015; **Record Level:** type: PhysicalObject; institutionCode: LETIA; basisOfRecord: PreservedSpecimen

#### Distribution

Brazil (Amazonas, Pará), Guyana, Suriname (Moreira 2021e).

#### Notes

First record from Pará State.

#### Photograph

Fig. 25d

### Stridulivelia alia

(Drake, 1957)

059068D2-9F7C-58DF-9BED-F6251E242803

Velia alia – see Drake (1957a): 115.
Stridulivelia alia – see Polhemus (1976): 509.

#### Materials

- **Type status:**Other material. **Occurrence:** recordedBy: S.E. Santos; sex: 4 apterous ?; **Location:** country: Brazil; stateProvince: Pará; municipality: Belterra; locality: BR-163, Km-115; verbatimLatitude: 03°17'34.8"S; verbatimLongitude: 54°52'45.6"W; **Event:** verbatimEventDate: 22.XI.2019; habitat: igarapé; **Record Level:** type: PhysicalObject; institutionCode: LETIA; basisOfRecord: PreservedSpecimen
- **Type status:**Other material. **Occurrence:** recordedBy: S.E. Santos; sex: 1 apterous ?; **Location:** country: Brazil; stateProvince: Pará; municipality: Santarém; locality: Igarapé da Débora; verbatimLatitude: 02°44'27.7"S; verbatimLongitude: 54°26'01.2"W; **Event:** verbatimEventDate: 21.X.2019; **Record Level:** type: PhysicalObject; institutionCode: LETIA; basisOfRecord: PreservedSpecimen

#### Distribution

Brazil (Amazonas, Pará), Guyana, Suriname, Venezuela (Moreira 2021e).

#### Notes

First records from the study area.

#### Photograph

Fig. 26a

### Stridulivelia quadrispinosa

(Hungerford, 1929)

51CDA6E2-72F7-5644-804C-90E2E5A919F8

Velia quadrispinosa – see Hungerford (1929b): 52, pl. I, figs. 2, 6 and 11, pl. II, fig. 3.
Stridulivelia quadrispinosa – see Polhemus (1976): 509.

#### Materials

- **Type status:**Other material. **Occurrence:** recordedBy: S.E. Santos; sex: 1 apterous ?; **Location:** country: Brazil; stateProvince: Pará; municipality: Mojuí dos Campos; locality: Igarapé Água Fria; verbatimLatitude: 02°47'19.7"S; verbatimLongitude: 54°38'40.9"W; **Event:** verbatimEventDate: 24.IX.2020; **Record Level:** type: PhysicalObject; institutionCode: LETIA; basisOfRecord: PreservedSpecimen

#### Distribution

Bolivia, Brazil (Alagoas, Espírito Santo, Mato Grosso, Minas Gerais, Pará, Rio de Janeiro), Guyana, Peru, Venezuela (Moreira 2021e).

#### Notes

Previously recorded from Santarém (Hungerford 1929b); first record from Mojuí dos Campos.

#### Photographs

Fig. 26b, c

### Stridulivelia stridulata

(Hungerford, 1929)

D90A3F45-71AC-57D0-8D17-3F27086ACF4B

Velia stridulata – see Hungerford (1929b): 53, pl. I, figs. 3 and 8, pl. II, fig. 6.
Stridulivelia stridulata – see Polhemus (1976): 509.

#### Materials

- **Type status:**Other material. **Occurrence:** recordedBy: S.E. Santos; sex: 2 apterous ?, 2 apterous ?; **Location:** country: Brazil; stateProvince: Pará; municipality: Santarém; locality: Igarapé Sonrizal; verbatimLatitude: 02°32'13.6''S; verbatimLongitude: 54°55'26.6''W; **Event:** verbatimEventDate: 09.VIII.2019; **Record Level:** type: PhysicalObject; institutionCode: LETIA; basisOfRecord: PreservedSpecimen

#### Distribution

Brazil (Amapá, Amazonas, Mato Grosso, Pará), Colombia, Suriname (Moreira 2021e).

#### Notes

First record from the study area.

#### Photograph

Fig. 26d

### Stridulivelia strigosa

(Hungerford, 1929)

6B158D7D-0057-5B7F-B630-C3BA7365EF07

Velia strigosa – see Hungerford (1929b): 50, pl. I, figs. 1, 7, pl. II, fig. 4.
Stridulivelia strigosa – see Polhemus (1976): 509.

#### Materials

- **Type status:**Other material. **Occurrence:** recordedBy: S.E. Santos; sex: 1 micropterous ?; **Location:** country: Brazil; stateProvince: Pará; municipality: Belterra; locality: Floresta Nacional do Tapajós; verbatimLatitude: 03°03'02.6"S; verbatimLongitude: 54°55'30.1"W; **Event:** verbatimEventDate: 20.I.2020; habitat: igarapé; **Record Level:** type: PhysicalObject; institutionCode: LETIA; basisOfRecord: PreservedSpecimen
- **Type status:**Other material. **Occurrence:** recordedBy: S.E. Santos; sex: 1 micropterous ?; **Location:** country: Brazil; stateProvince: Pará; municipality: Mojuí dos Campos; locality: Igarapé Terra de Areia; verbatimLatitude: 02°47'58.7"S; verbatimLongitude: 54°38'15.6"W; **Event:** verbatimEventDate: 24.I.2020; **Record Level:** type: PhysicalObject; institutionCode: LETIA; basisOfRecord: PreservedSpecimen
- **Type status:**Other material. **Occurrence:** recordedBy: S.E. Santos; sex: 1 apterous ?; **Location:** country: Brazil; stateProvince: Pará; municipality: Santarém; locality: Igarapé Sonrizal; verbatimLatitude: 02°32'13.6"S; verbatimLongitude: 54°55'26.6"W; **Event:** verbatimEventDate: 09.VIII.2019; **Record Level:** type: PhysicalObject; institutionCode: LETIA; basisOfRecord: PreservedSpecimen

#### Distribution

Brazil (Amapá, Amazonas, Mato Grosso, Pará), French Guiana, Guyana, Peru, Suriname, Venezuela (Moreira 2021e).

#### Notes

First records from the study area.

#### Photographs

Fig. 26e, f

### Stridulivelia tersa

(Drake & Harris, 1941)

C1D7DFEF-6C6D-50A9-8D41-AE074B712136

Velia tersa – see Drake and Harris (1941b): 338.
Velia nama – see Drake (1957a): 114 (syn. by Polhemus and Spangler 1995: 147).
Stridulivelia tersa – see Polhemus (1976): 509.

#### Materials

- **Type status:**Other material. **Occurrence:** recordedBy: S.E. Santos; sex: 1 micropterous ?; **Location:** country: Brazil; stateProvince: Pará; municipality: Belterra; locality: Igarapé Jatuarana; verbatimLatitude: 03°15'44.7"S; verbatimLongitude: 54°56'37.5"W; **Event:** verbatimEventDate: 11.II.2020; **Record Level:** type: PhysicalObject; institutionCode: LETIA; basisOfRecord: PreservedSpecimen
- **Type status:**Other material. **Occurrence:** recordedBy: S.E. Santos; sex: 1 macropterous ?; **Location:** country: Brazil; stateProvince: Pará; municipality: Mojuí dos Campos; locality: Igarapé Água Fria; verbatimLatitude: 02°47'19.7"S; verbatimLongitude: 54°38'40.9"W; **Event:** verbatimEventDate: 24.IX.2020; **Record Level:** type: PhysicalObject; institutionCode: LETIA; basisOfRecord: PreservedSpecimen
- **Type status:**Other material. **Occurrence:** recordedBy: S.E. Santos; sex: 1 macropterous ?, 2 macropterous ?, 2 micropterous ?, 1 micropterous ?; **Location:** country: Brazil; stateProvince: Pará; municipality: Mojuí dos Campos; locality: Igarapé do Manel; verbatimLatitude: 02°25'06.3"S; verbatimLongitude: 54°44'26.3"W; **Event:** verbatimEventDate: 24.II.2020; **Record Level:** type: PhysicalObject; institutionCode: LETIA; basisOfRecord: PreservedSpecimen
- **Type status:**Other material. **Occurrence:** recordedBy: S.E. Santos; sex: 2 macropterous ?, 3 macropterous ?; **Location:** country: Brazil; stateProvince: Pará; municipality: Santarém; locality: Igarapé Diamantino; verbatimLatitude: 02°30'16.2"S; verbatimLongitude: 54°39'32.9"W; **Event:** verbatimEventDate: 06.IX.2019; **Record Level:** type: PhysicalObject; institutionCode: LETIA; basisOfRecord: PreservedSpecimen
- **Type status:**Other material. **Occurrence:** recordedBy: S.E. Santos; sex: 2 macropterous ?; **Location:** country: Brazil; stateProvince: Pará; municipality: Santarém; locality: Cachoeira da Cavada; verbatimLatitude: 02°35'48.9"S; verbatimLongitude: 54°49'29.7"W; **Event:** verbatimEventDate: 13.XI.2019; **Record Level:** type: PhysicalObject; institutionCode: LETIA; basisOfRecord: PreservedSpecimen
- **Type status:**Other material. **Occurrence:** recordedBy: S.E. Santos; sex: 1 macropterous ?; **Location:** country: Brazil; stateProvince: Pará; municipality: Santarém; locality: Cachoeira da Cavada; verbatimLatitude: 02°35'48.9"S; verbatimLongitude: 54°49'29.7"W; **Event:** verbatimEventDate: 21.X.2019; **Record Level:** type: PhysicalObject; institutionCode: LETIA; basisOfRecord: PreservedSpecimen
- **Type status:**Other material. **Occurrence:** recordedBy: M. Galúcio; sex: 2 apterous ?; **Location:** country: Brazil; stateProvince: Pará; municipality: Santarém; locality: Igarapé Vila Nova; verbatimLatitude: 02°30'50.4"S; verbatimLongitude: 54°49'29.7"W; **Event:** verbatimEventDate: 10.X.2015; **Record Level:** type: PhysicalObject; institutionCode: LETIA; basisOfRecord: PreservedSpecimen
- **Type status:**Other material. **Occurrence:** recordedBy: M. Galúcio; sex: 1 apterous ?; **Location:** country: Brazil; stateProvince: Pará; municipality: Santarém; locality: Igarapé Urumari; verbatimLatitude: 02°28'25.3"S; verbatimLongitude: 54°41'52.3"W; **Event:** verbatimEventDate: 19.X.2015; **Record Level:** type: PhysicalObject; institutionCode: LETIA; basisOfRecord: PreservedSpecimen

#### Distribution

Bolivia, Brazil (Alagoas, Amazonas, Espírito Santo, Mato Grosso, Minas Gerais, Pará, Sergipe), Colombia, Guyana, Peru, Suriname, Trinidad and Tobago, Venezuela (Moreira 2021e).

#### Notes

First records from the study area.

#### Photographs

Fig. 27a, b

### Stridulivelia transversa

(Hungerford, 1929)

DCE8C7CB-8A51-509A-9B5B-8DBE25E705CC

Velia transversa – see Hungerford (1929b): 54, pl. I, fig. 10, pl. II, fig. 7.
Stridulivelia transversa – see Polhemus (1976): 509.

#### Materials

- **Type status:**Other material. **Occurrence:** recordedBy: S.E. Santos; sex: 2 apterous ?, 4 apterous ?; **Location:** country: Brazil; stateProvince: Pará; municipality: Belterra; locality: BR-163, Km-115; verbatimLatitude: 03°17'34.8"S; verbatimLongitude: 54°52'45.6"W; **Event:** verbatimEventDate: 23.X.2019; habitat: igarapé; **Record Level:** type: PhysicalObject; institutionCode: LETIA; basisOfRecord: PreservedSpecimen
- **Type status:**Other material. **Occurrence:** recordedBy: S.E. Santos; sex: 1 micropterous ?; **Location:** country: Brazil; stateProvince: Pará; municipality: Belterra; locality: Igarapé do Ailton; verbatimLatitude: 02°35'36.7"S; verbatimLongitude: 54°57'48.4"W; **Event:** verbatimEventDate: 06.XI.2019; **Record Level:** type: PhysicalObject; institutionCode: LETIA; basisOfRecord: PreservedSpecimen
- **Type status:**Other material. **Occurrence:** recordedBy: S.E. Santos; sex: 1 apterous ?; **Location:** country: Brazil; stateProvince: Pará; municipality: Santarém; locality: Cachoeira da Cavada; verbatimLatitude: 02°35'48.9"S; verbatimLongitude: 54°31'47.3"W; **Event:** verbatimEventDate: 13.XI.2019; **Record Level:** type: PhysicalObject; institutionCode: LETIA; basisOfRecord: PreservedSpecimen
- **Type status:**Other material. **Occurrence:** recordedBy: S.E. Santos; sex: 1 apterous ?; **Location:** country: Brazil; stateProvince: Pará; municipality: Santarém; locality: Igarapé Mararú; verbatimLatitude: 02°29'35''S; verbatimLongitude: 54°40'06''W; **Event:** verbatimEventDate: 23.VIII.2019; **Record Level:** type: PhysicalObject; institutionCode: LETIA; basisOfRecord: PreservedSpecimen
- **Type status:**Other material. **Occurrence:** recordedBy: S.E. Santos; sex: 1 apterous ?, 2 apterous ?; **Location:** country: Brazil; stateProvince: Pará; municipality: Santarém; locality: Igarapé da Débora; verbatimLatitude: 02°44'27.7"S; verbatimLongitude: 54°26'01.2"W; **Event:** verbatimEventDate: 21.X.2019; **Record Level:** type: PhysicalObject; institutionCode: LETIA; basisOfRecord: PreservedSpecimen
- **Type status:**Other material. **Occurrence:** recordedBy: S.E. Santos; sex: 1 apterous ?; **Location:** country: Brazil; stateProvince: Pará; municipality: Santarém; locality: Igarapé Cajutuba II; verbatimLatitude: 02°27'39.2"S; verbatimLongitude: 54°46'53.4"W; **Event:** verbatimEventDate: 10.X.2020; **Record Level:** type: PhysicalObject; institutionCode: LETIA; basisOfRecord: PreservedSpecimen

#### Distribution

Brazil (Amapá, Amazonas, Pará), French Guiana, Suriname, Venezuela (Moreira 2021e).

#### Notes

First records from the study area.

#### Photograph

Fig. 27c

## Discussion

Our survey of the semi-aquatic bugs from the MRS revealed the occurrence of 14 genera and 44 species belonging to the families Gerridae, Hydrometridae, Mesoveliidae and Veliidae in the study region (Table 2). This represents a 100% increase in relation to the specific diversity previously recorded from the area in literature (Champion 1898, Hungerford 1929b, Drake and Harris 1930, Kuitert 1942, Bacon 1948, Hungerford 1954, Neering 1954, Bacon 1956, Nieser 1970, Polhemus 1997, Moreira et al. 2008, Moreira et al. 2011a, Rodrigues et al. 2014a). Amongst the recorded species, *Microveliabelterrensis* **sp. nov.**, *M.hamadae* **sp. nov.** and *M.sousorum* **sp. nov.** are described as new and, together with *M.aschnakiranae*, newly recorded from Brazil, increase the number of species of the genus known to occur in the country from 29 to 33 (Moreira 2021e). This contributes to fill a gap of knowledge about the genus in South America, where many species of Microveliinae remain undescribed (Polhemus and Polhemus 2007). Additionally, the recently-described *Rhagoveliagraziae* is recorded from Brazil for the first time, increasing the distribution range of the species by more than 2000 km eastwards, from the Colombian Llanos to the Brazilian Amazon (Galindo-Malagón et al. 2021). Finally, *M.longipes* and *Paraveliadilatata* are recorded for the first time from Pará State. The former is quite common in temporary water bodies in South America and the new records fill a gap in its distribution between Roraima and Amazonas States and north-eastern Brazil (Polhemus 1990, Moreira and Campos 2012, Rodrigues et al. 2012, Cordeiro and Moreira 2015, Rodrigues et al. 2021). The latter is a much rarer species, known from less than 10 localities in Guyana, Suriname and Brazil (Amazonas State) (Polhemus 2014, Polhemus and Polhemus 1984, Pereira and Melo 2007, Rodrigues et al. 2014b, Rodrigues and Moreira 2016). Our record extends the known distribution of the species by about 600 km to the east of the previous records in its southern edge, in Manaus, Amazonas State, Brazil (Polhemus and Polhemus 1984, Polhemus 2014, Rodrigues et al. 2014b, Rodrigues and Moreira 2016).

## Supplementary Material

XML Treatment for Brachymetra lata

XML Treatment for Brachymetra shawi

XML Treatment for Cylindrostethus drakei

XML Treatment for Cylindrostethus palmaris

XML Treatment for Limnogonus aduncus aduncus

XML Treatment for Limnogonus recurvus

XML Treatment for Neogerris genticus

XML Treatment for Neogerris lotus

XML Treatment for Neogerris lubricus

XML Treatment for Neogerris visendus

XML Treatment for Tachygerris adamsoni

XML Treatment for Rheumatobates crassifemur esakii

XML Treatment for Rheumatobates klagei

XML Treatment for Hydrometra argentina

XML Treatment for Mesovelia mulsanti

XML Treatment for Mesovelia zeteki

XML Treatment for Microvelia aschnakiranae

XML Treatment for Microvelia belterrensis

XML Treatment for Microvelia hamadae

XML Treatment for Microvelia longipes

XML Treatment for Microvelia mimula

XML Treatment for Microvelia pulchella

XML Treatment for Microvelia sousorum

XML Treatment for Microvelia summersi

XML Treatment for Microvelia venustatis

XML Treatment for Rhagovelia amazonensis

XML Treatment for Rhagovelia brunae

XML Treatment for Rhagovelia elegans

XML Treatment for Rhagovelia evidis

XML Treatment for Rhagovelia graziae

XML Treatment for Rhagovelia jubata

XML Treatment for Rhagovelia tenuipes

XML Treatment for Rhagovelia trailii

XML Treatment for Callivelia conata

XML Treatment for Oiovelia chenae

XML Treatment for Oiovelia cunucunumana

XML Treatment for Paravelia bullialata

XML Treatment for Paravelia dilatata

XML Treatment for Stridulivelia alia

XML Treatment for Stridulivelia quadrispinosa

XML Treatment for Stridulivelia stridulata

XML Treatment for Stridulivelia strigosa

XML Treatment for Stridulivelia tersa

XML Treatment for Stridulivelia transversa

## Figures and Tables

**Figure 4a.**
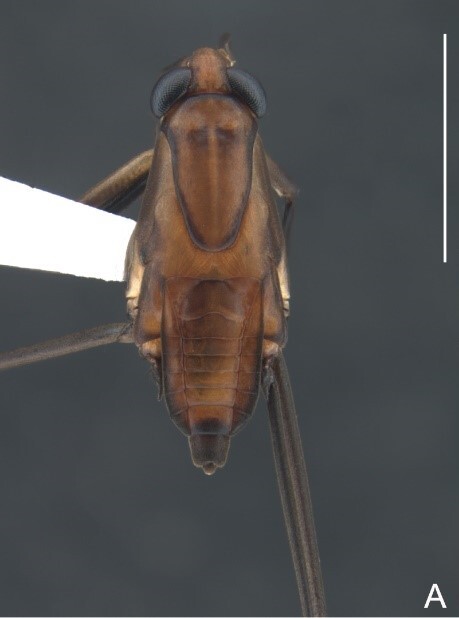
*B.lata*, apterous male

**Figure 4b.**
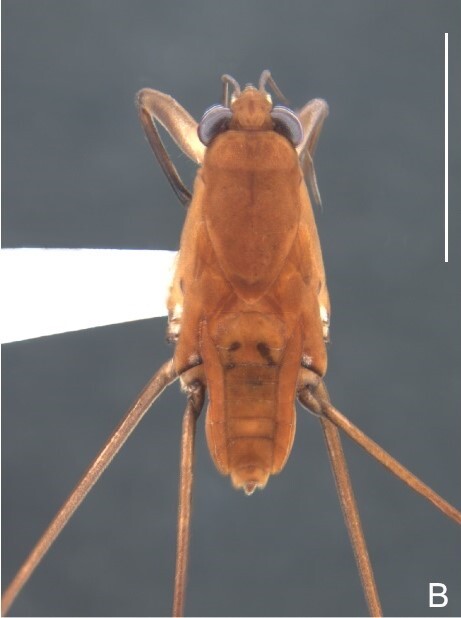
*B.shawi*, apterous male

**Figure 4c.**
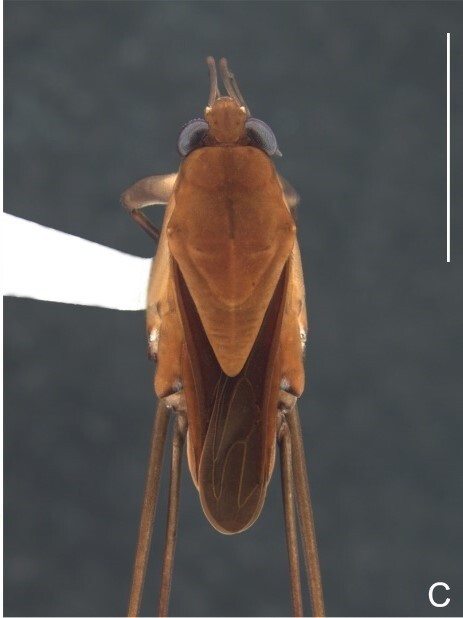
*B.shawi*, macropterous male

**Figure 6a.**
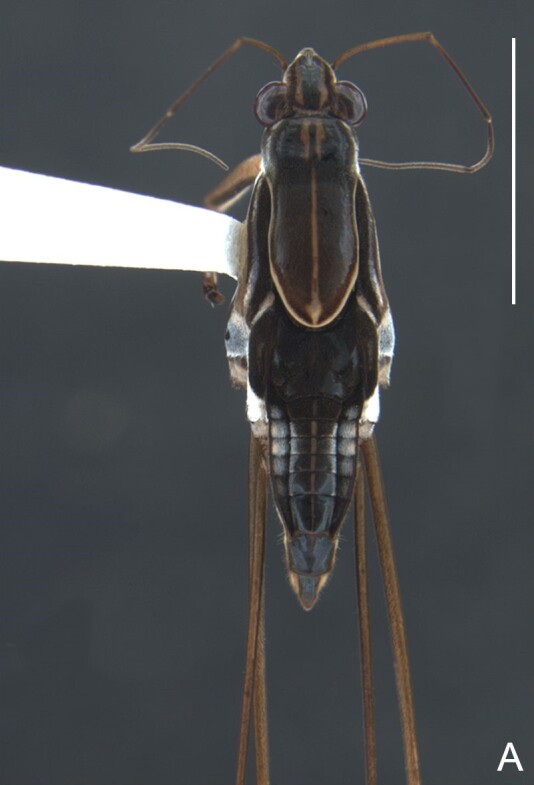
*Limnogonusaduncusaduncus*, apterous male

**Figure 6b.**
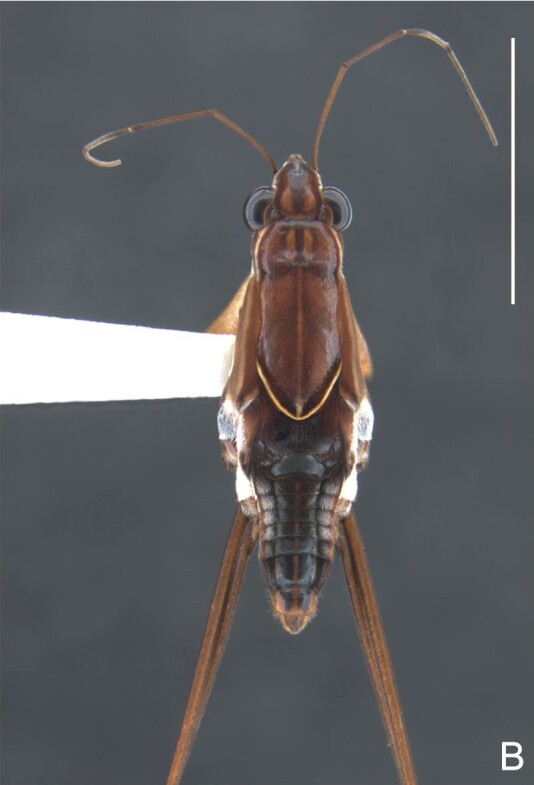
*L.recurvus*, apterous male

**Figure 6c.**
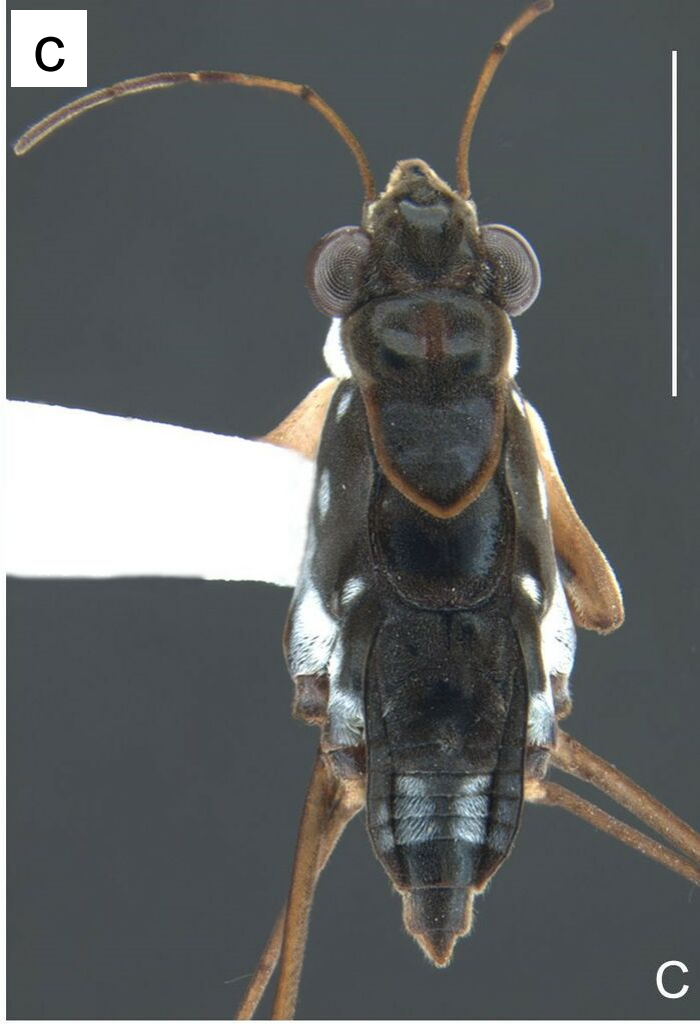
*Neogerrisgenticus*, apterous male

**Figure 6d.**
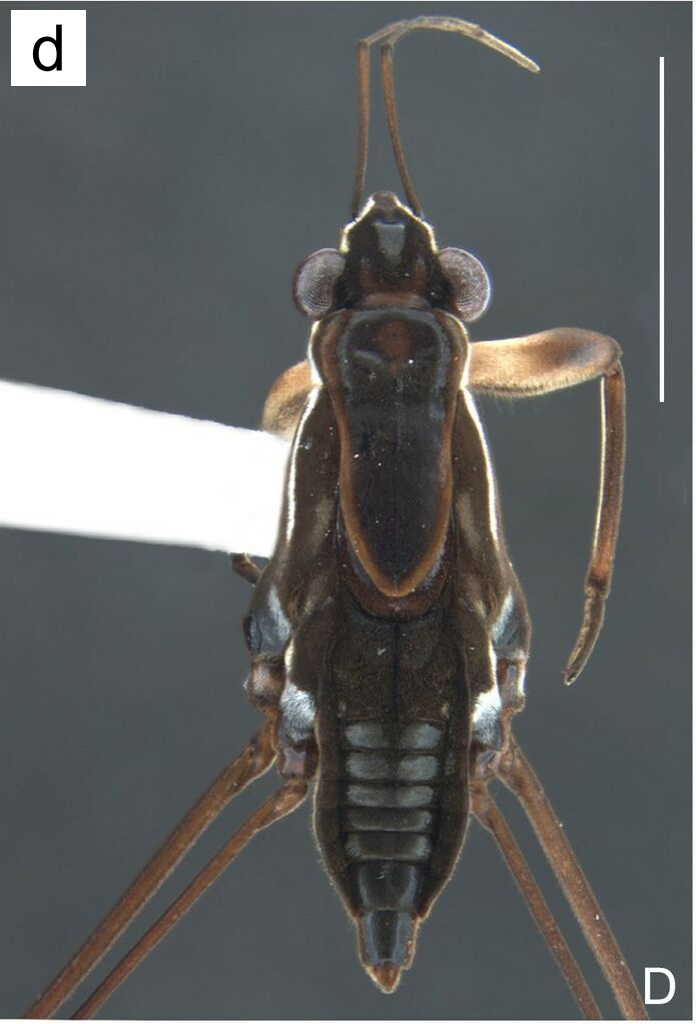
*Neogerrislubricus*, apterous male

**Figure 6e.**
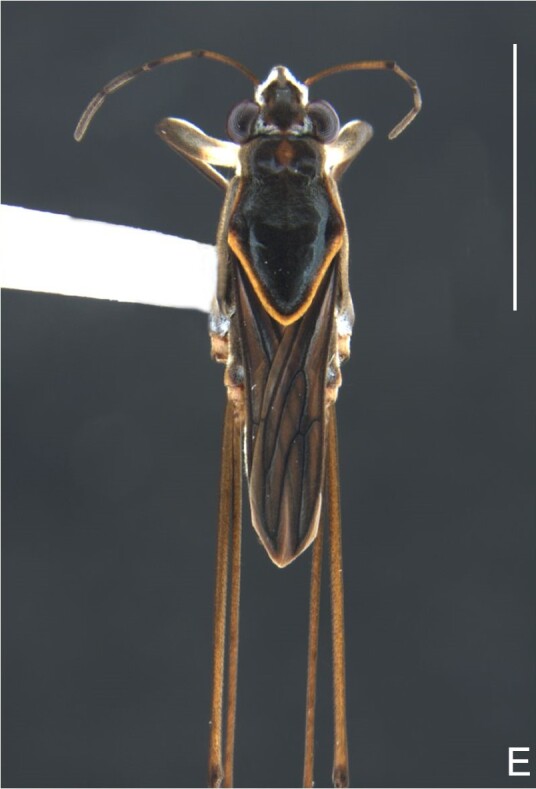
*N.visendus*, macropterous male

**Figure 6f.**
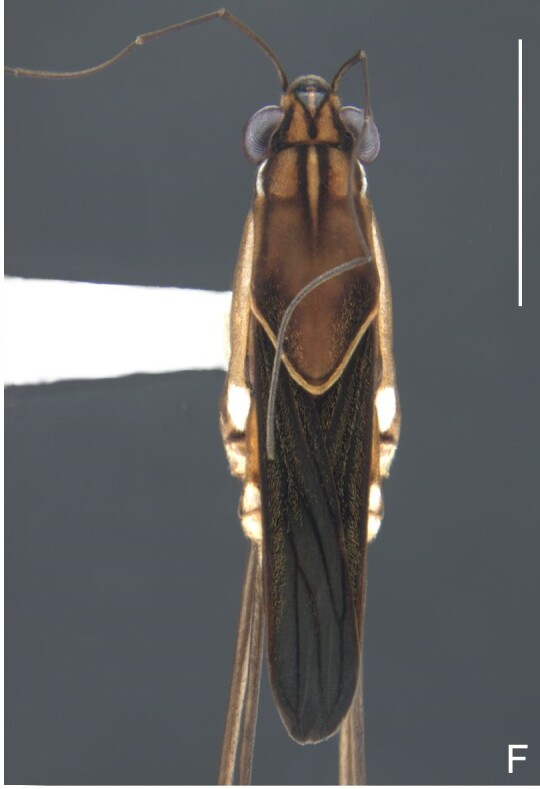
*Tachygerrisadamsoni*, macropterous male

**Figure 9.**
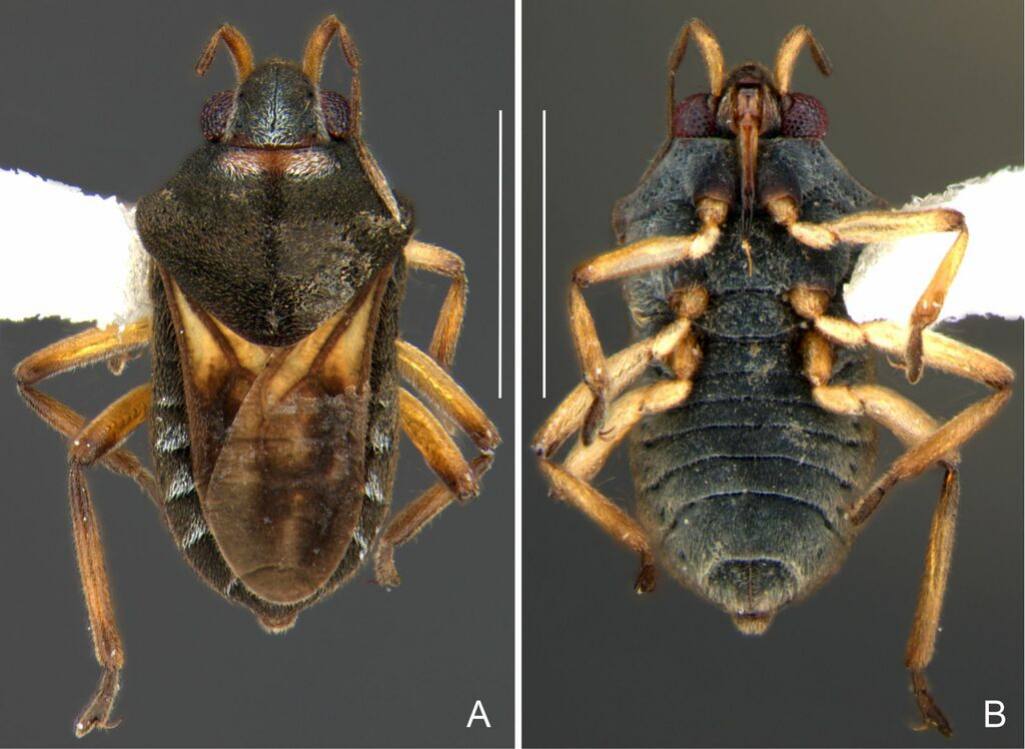
*Microveliaaschnakiranae*, macropterous female, habitus **A.** Dorsal view; **B.** Ventral view. Scale bars: 1mm.

**Figure 13a.**
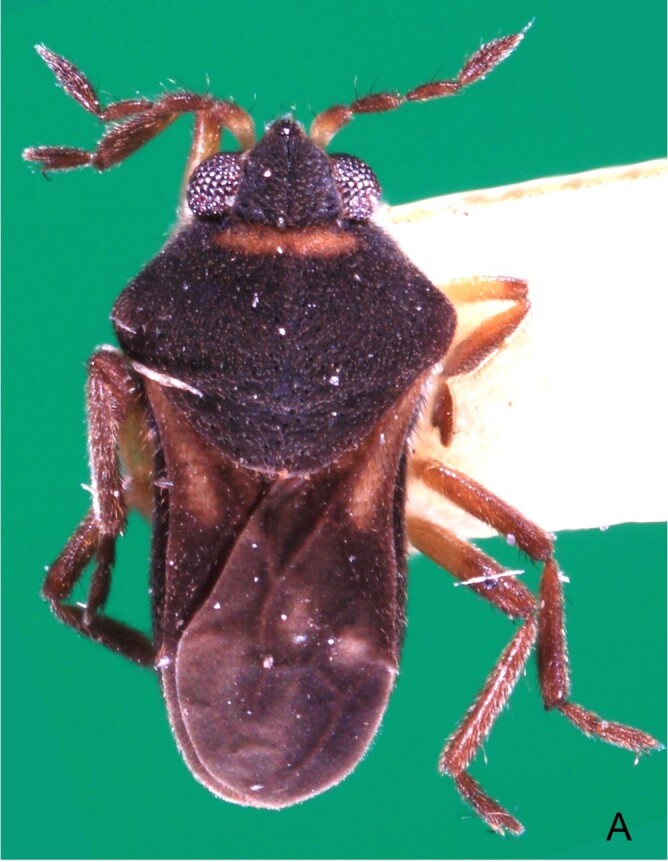
*Microveliavenustatis*, macropterous male, habitus, dorsal view

**Figure 13b.**
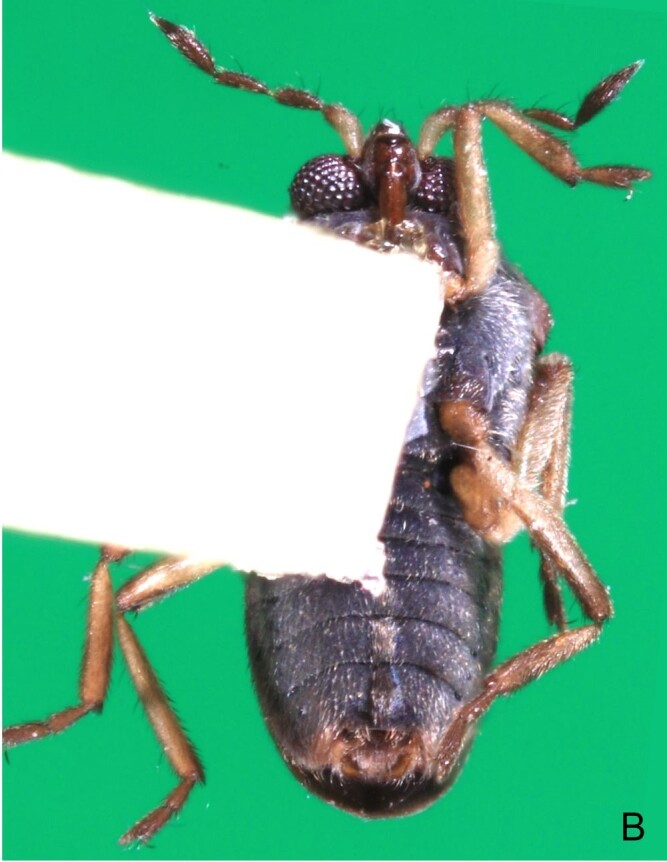
*M.venustatis*, macropterous male, habitus, ventral view

**Figure 13c.**
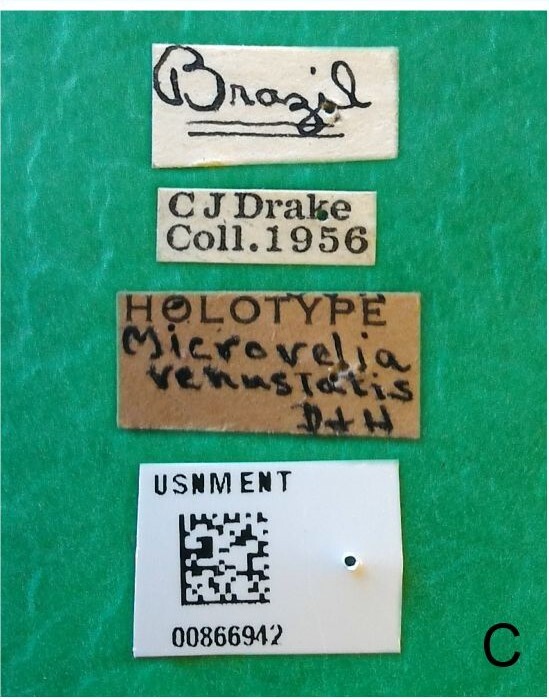
*M.venustatis*, labels

**Figure 13d.**
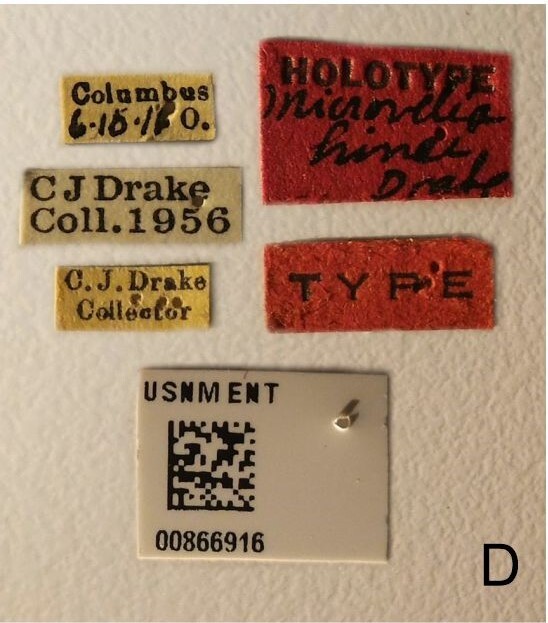
*M.hinei*, labels

**Figure 13e.**
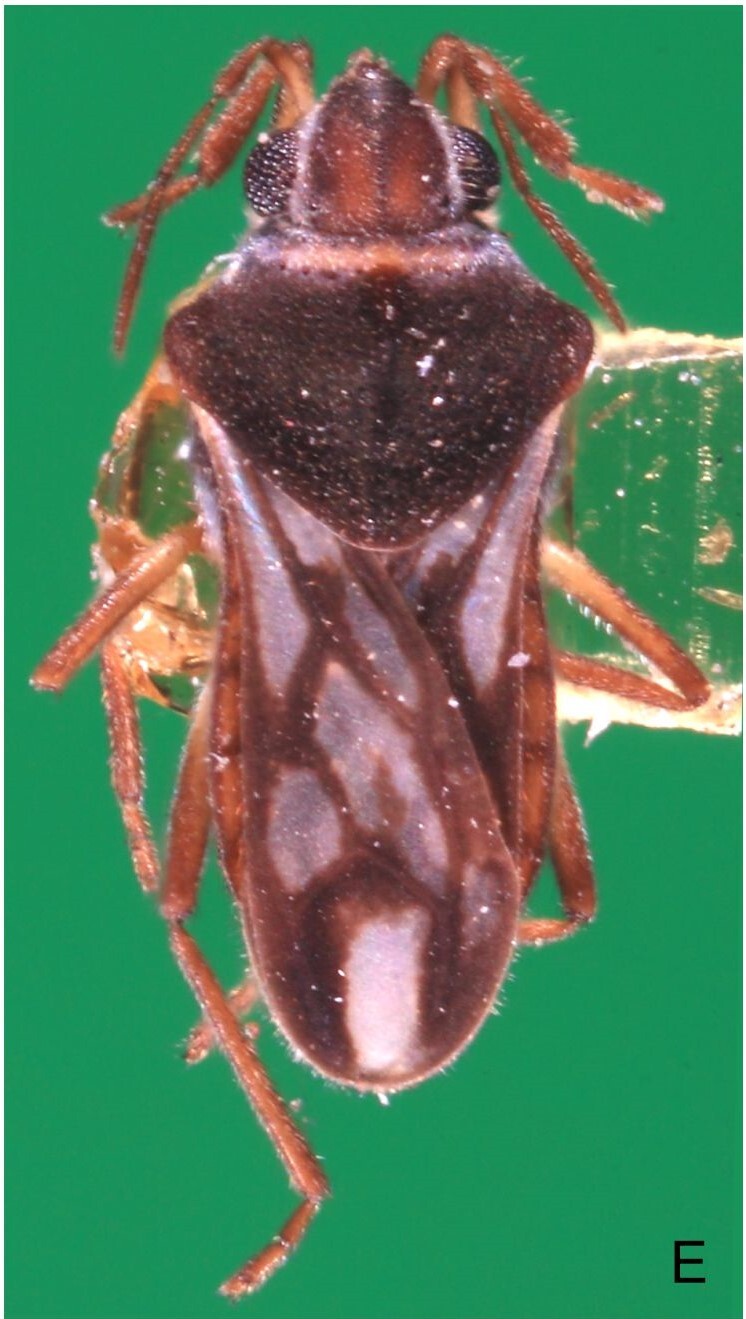
*M.hinei*, macropterous male, habitus, dorsal view

**Figure 13f.**
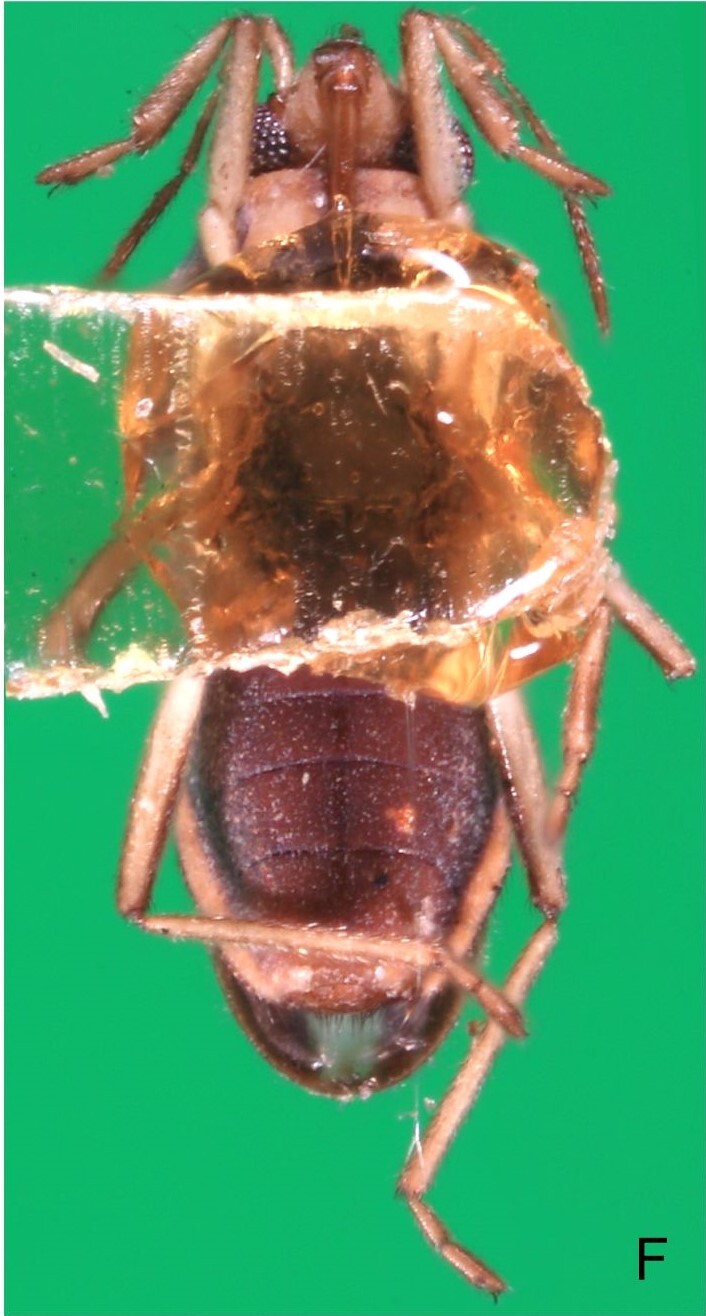
*M.hinei*, macropterous male, habitus, ventral view

**Figure 17a.**
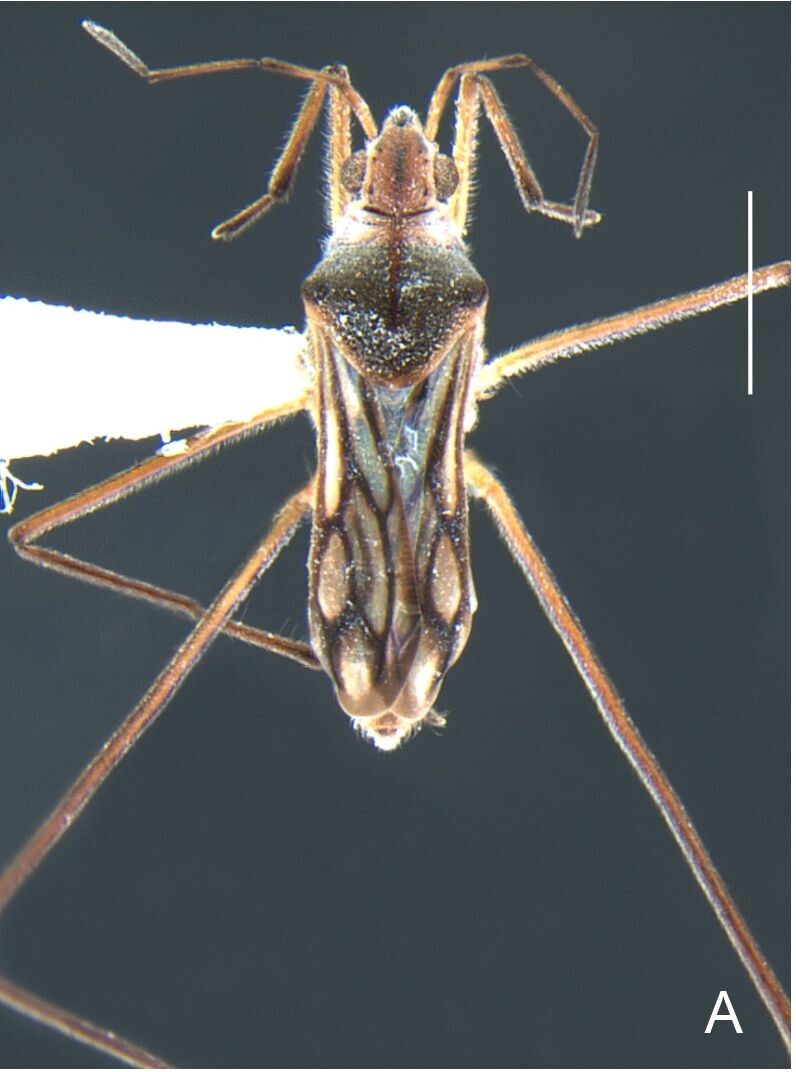
*M.longipes*, macropterous male, dorsal view

**Figure 17b.**
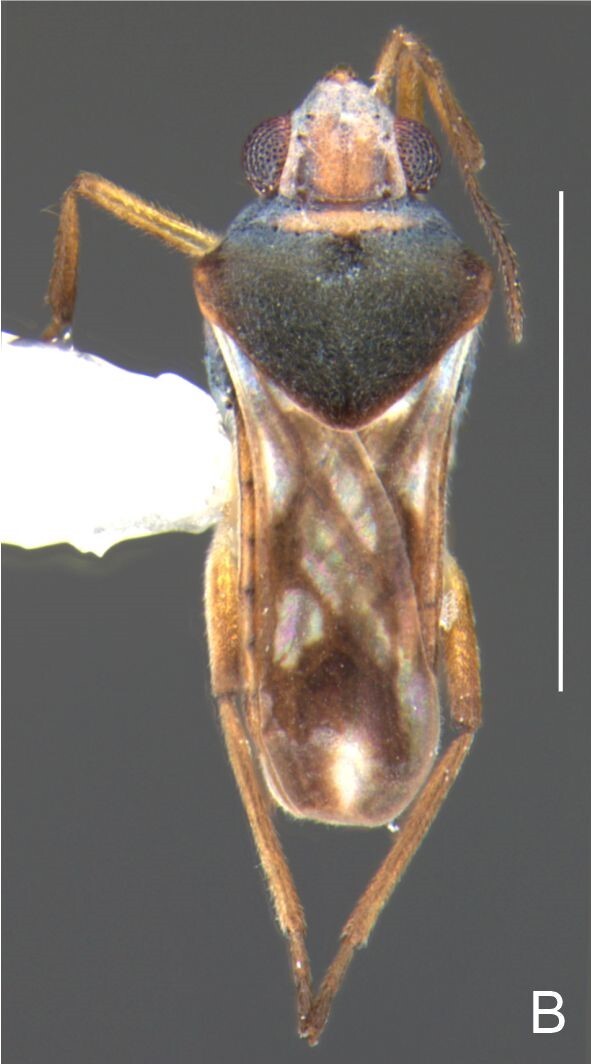
*M.mimula*, macropterous male, dorsal view

**Figure 17c.**
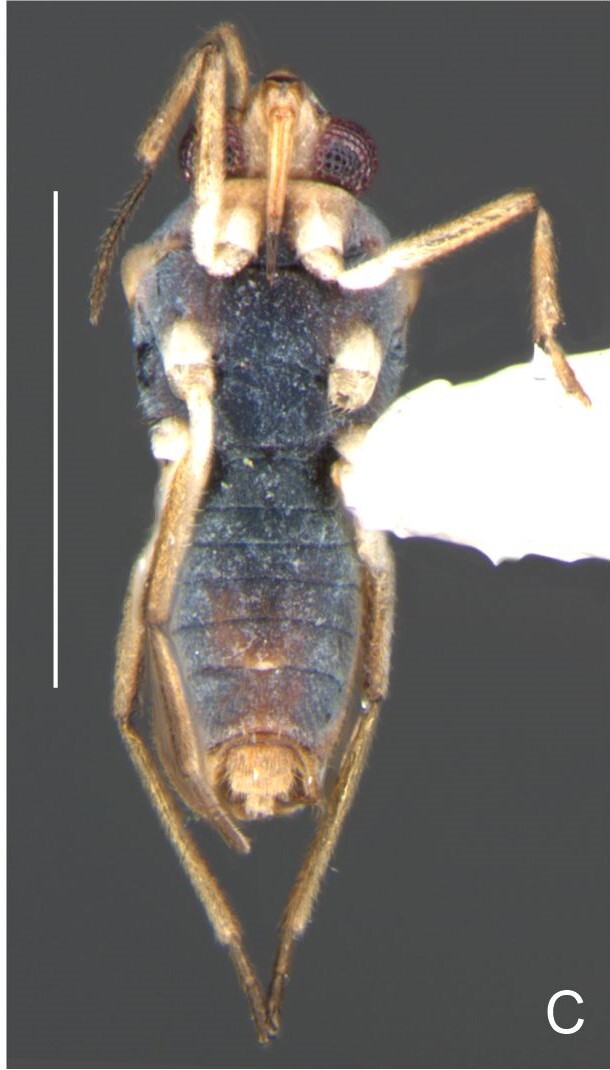
*M.mimula*, macropterous male, ventral view

**Figure 17d.**
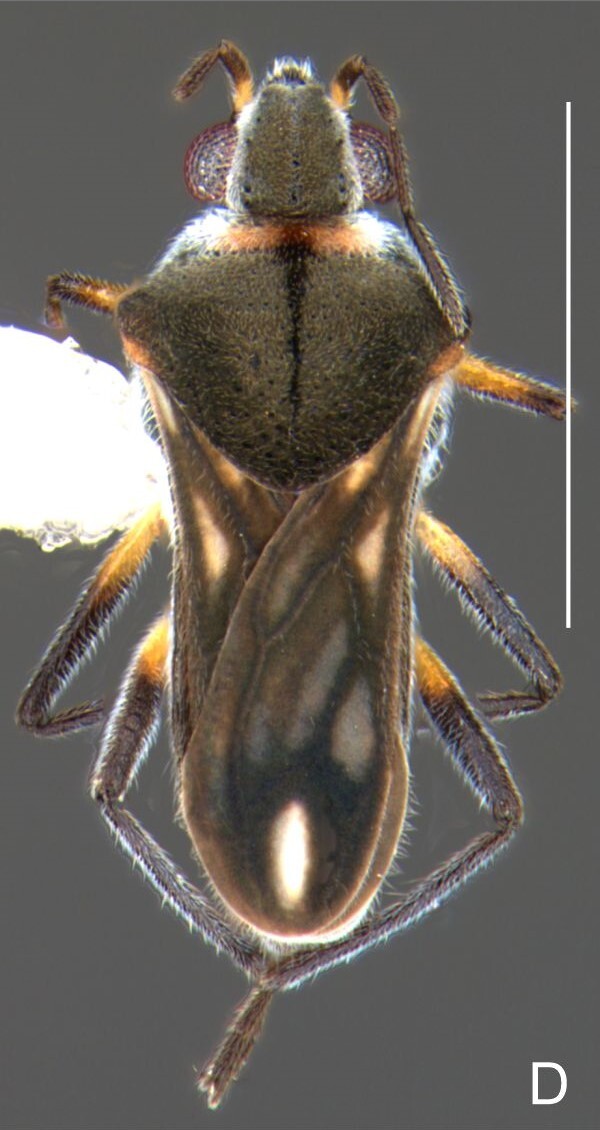
*M.pulchella*, macropterous male, dorsal view

**Figure 17e.**
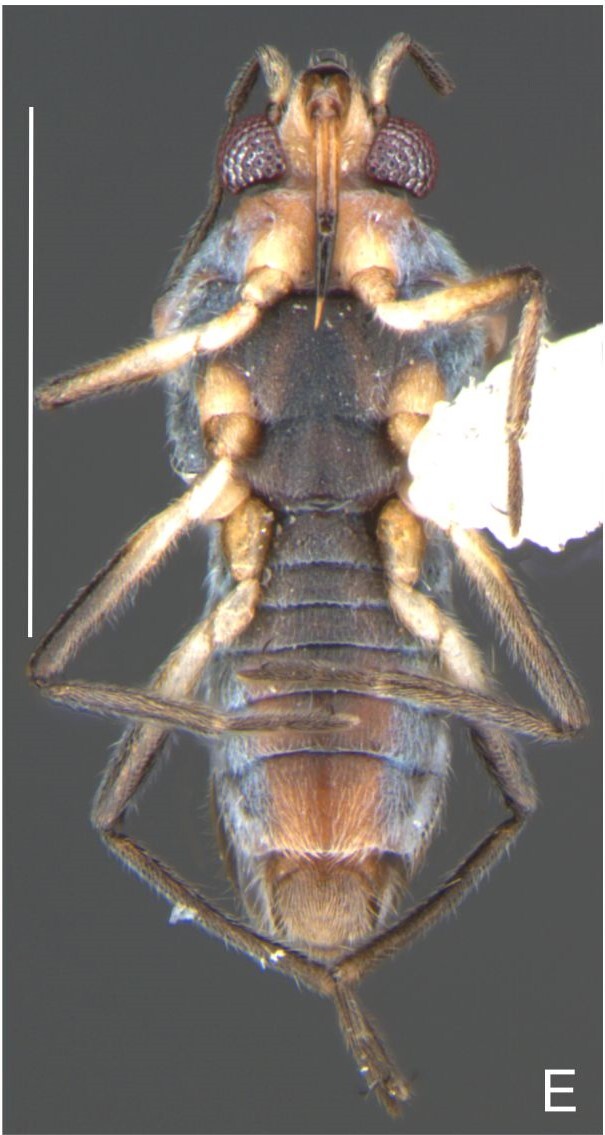
*M.pulchella*, macropterous male, ventral view

**Figure 17f.**
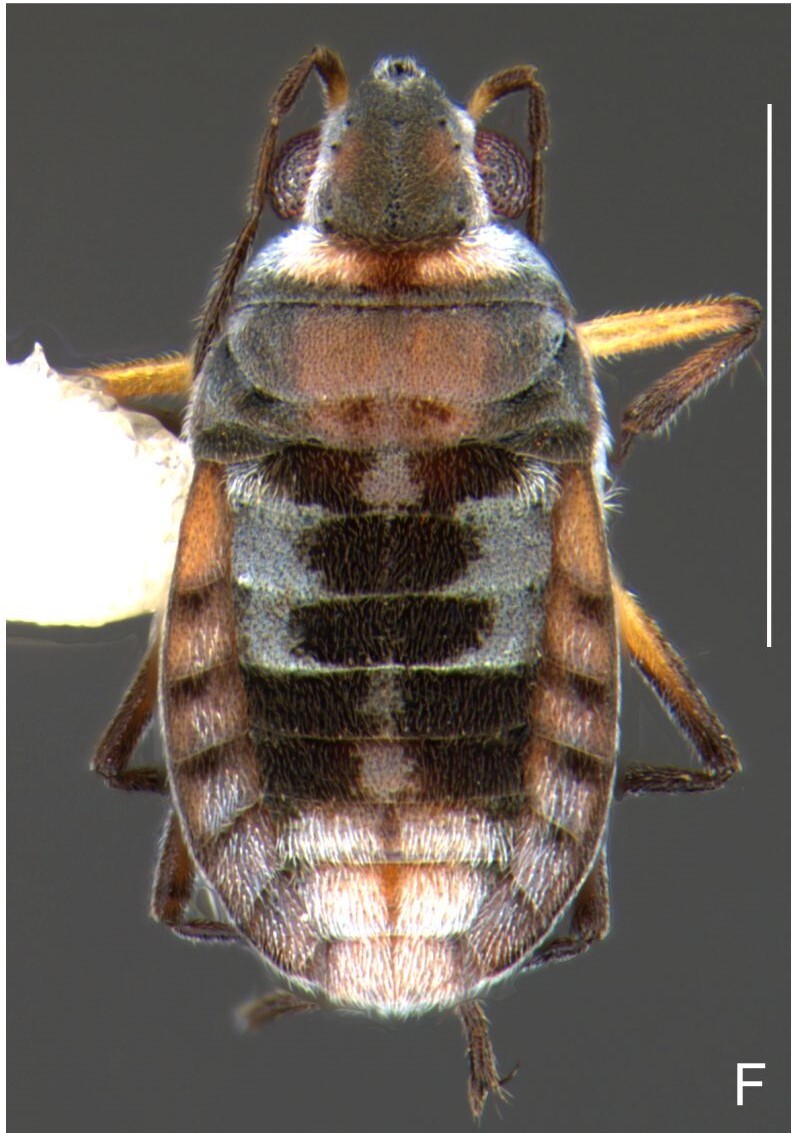
*M.pulchella*, apterous female, dorsal view

**Figure 23a.**
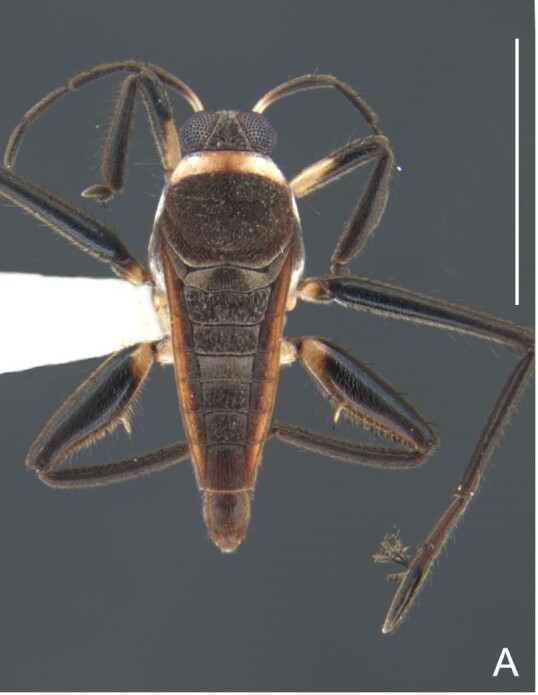
*R.brunae*, apterous male

**Figure 23b.**
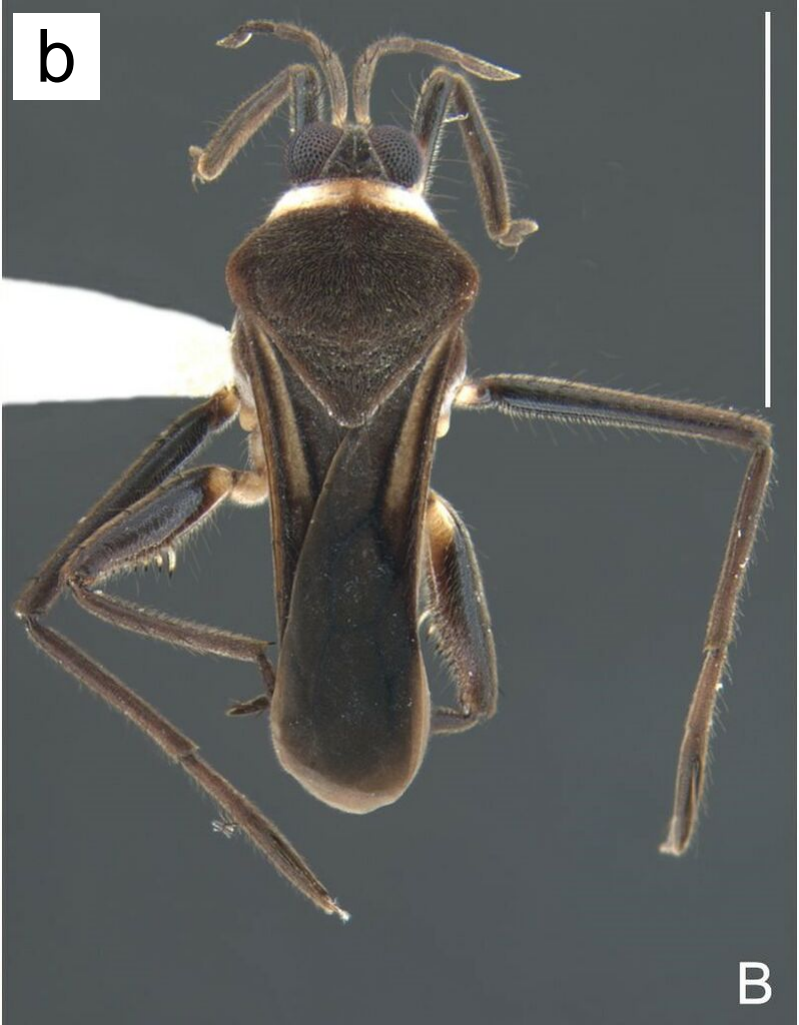
*R.brunae*, macropterous male

**Figure 23c.**
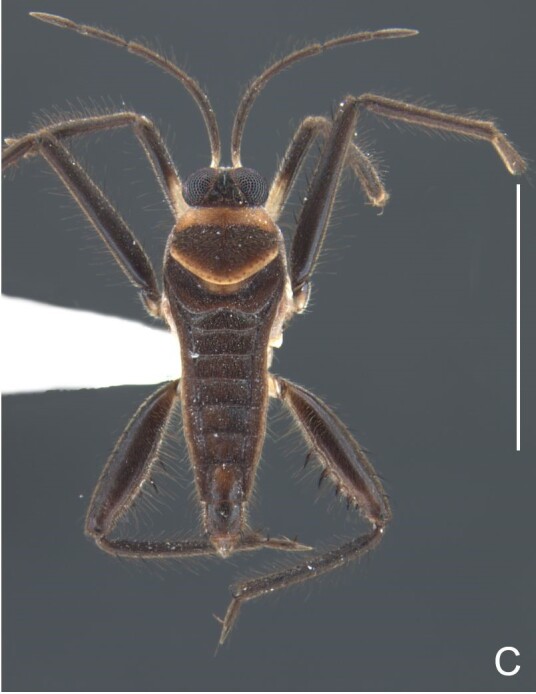
*R.elegans*, apterous male

**Figure 23d.**
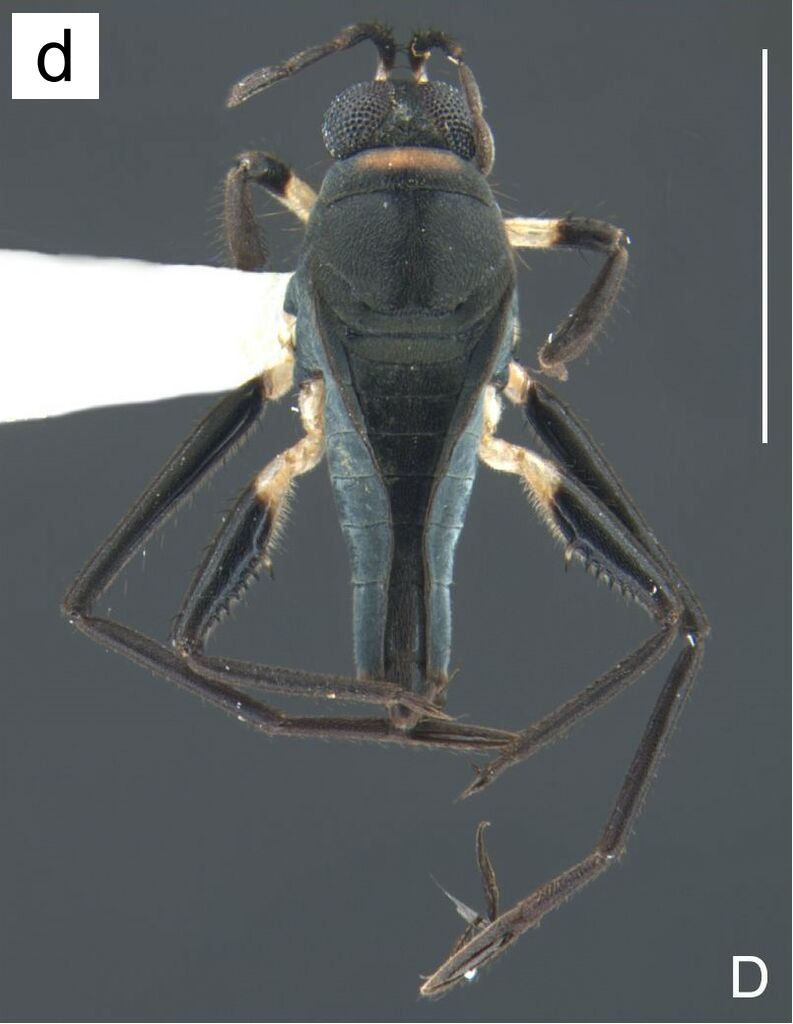
*R.evidis*, apterous female

**Figure 23e.**
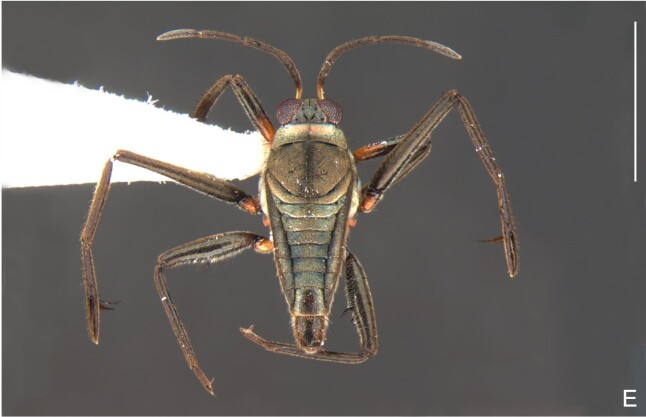
*R.graziae*, apterous male

**Figure 23f.**
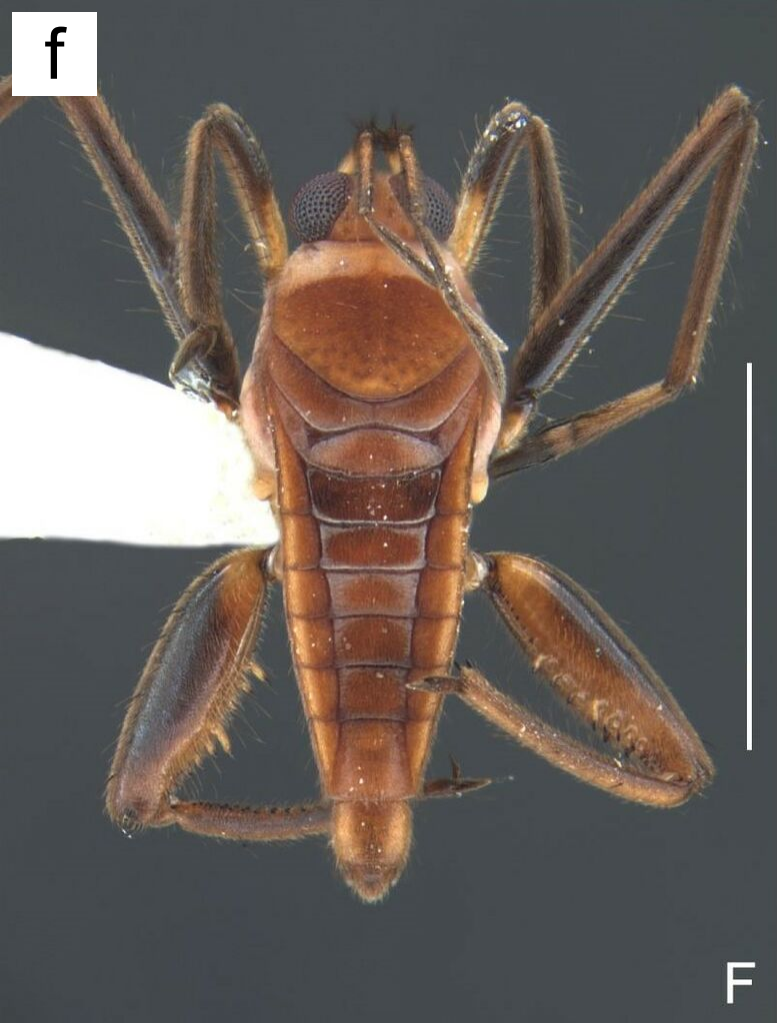
*R.jubata*, apterous male

**Figure 24.**
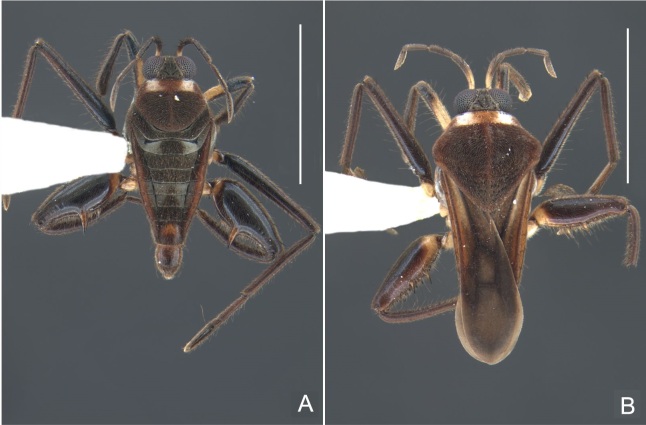
*Rhagoveliatrailii*, habitus, dorsal view **A.** Apterous male; **B.** Macropterous male. Scale bars: 1 mm.

**Figure 25a.**
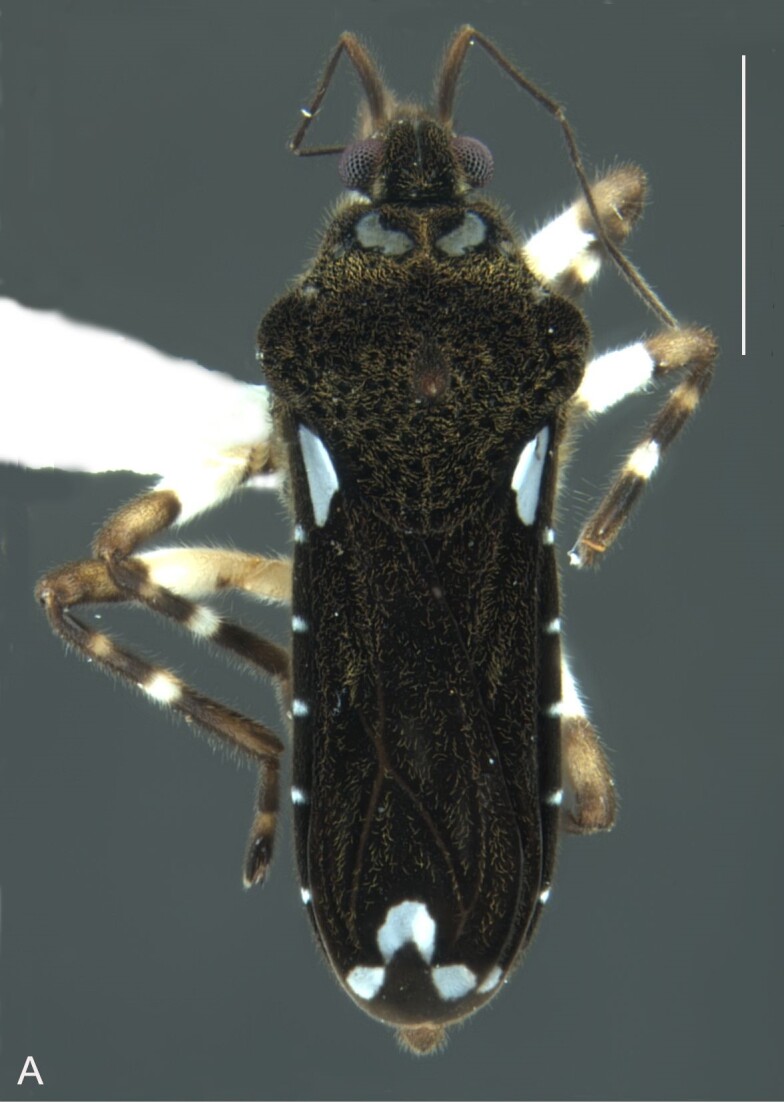
*Calliveliaconata*, macropterous female

**Figure 25b.**
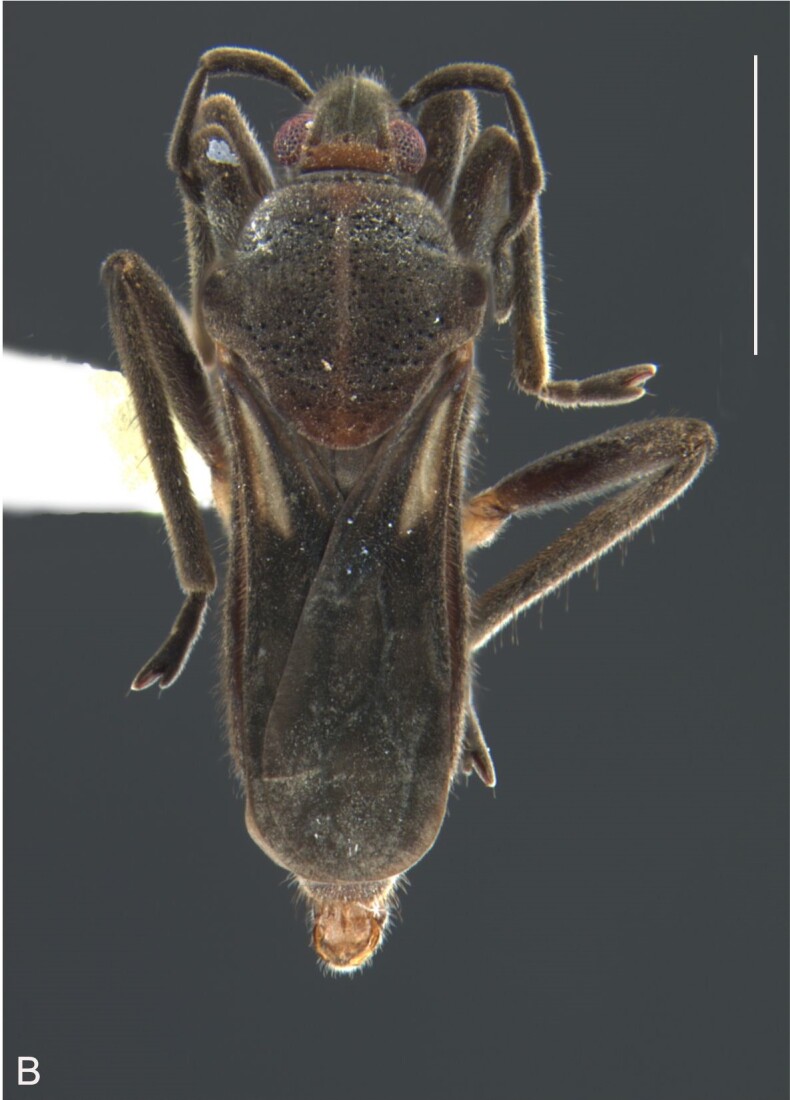
*Oioveliacunucunumana*, macropterous male

**Figure 25c.**
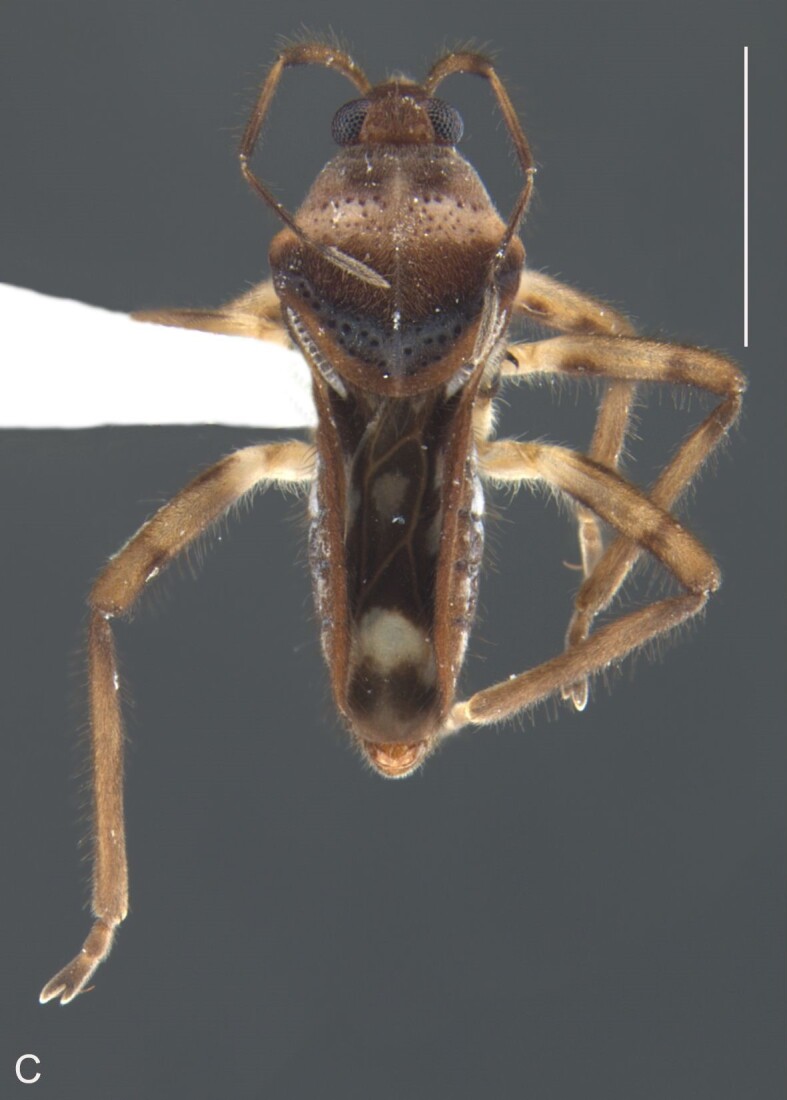
*Paraveliabullialata*, macropterous male

**Figure 25d.**
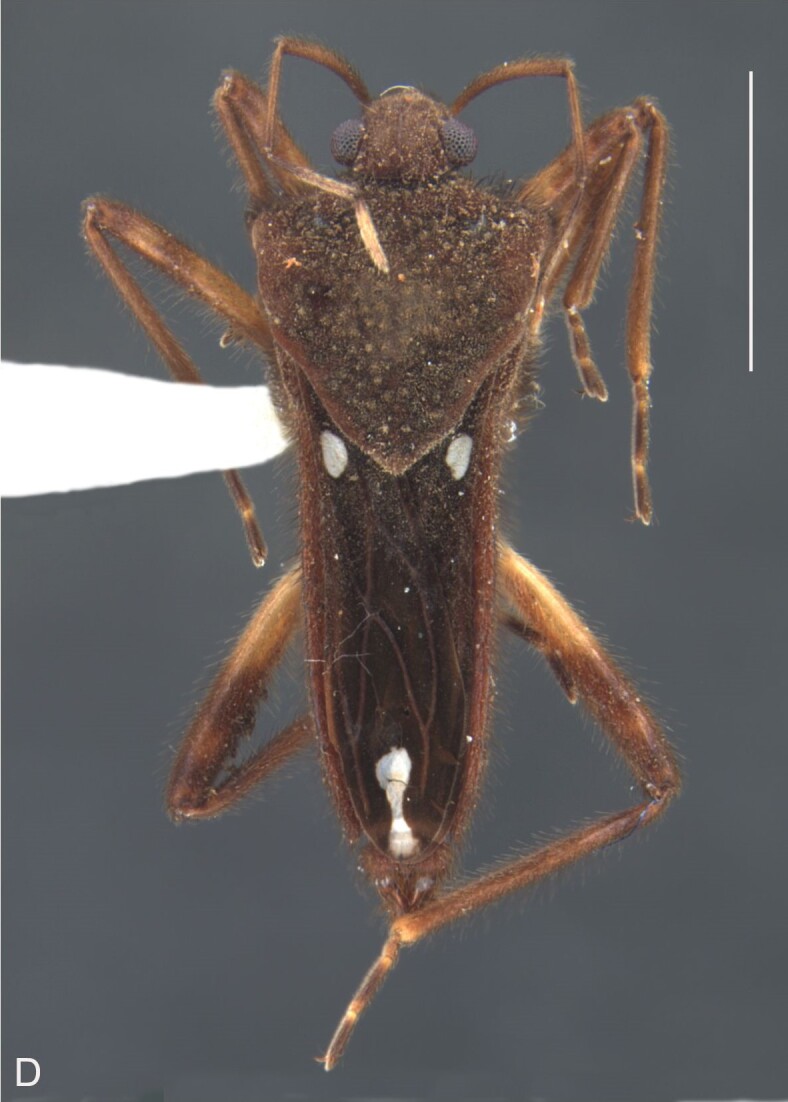
*Paraveliadilatata*, macropterous male

**Figure 26a.**
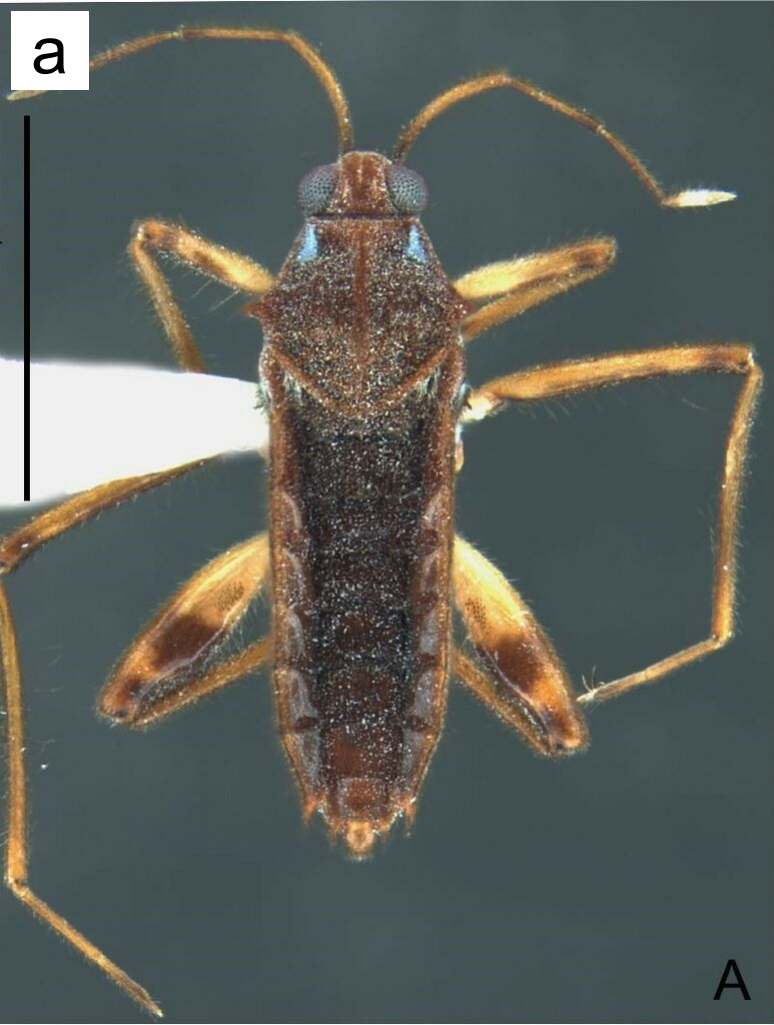
*S.alia*, apterous female, habitus, dorsal view

**Figure 26b.**
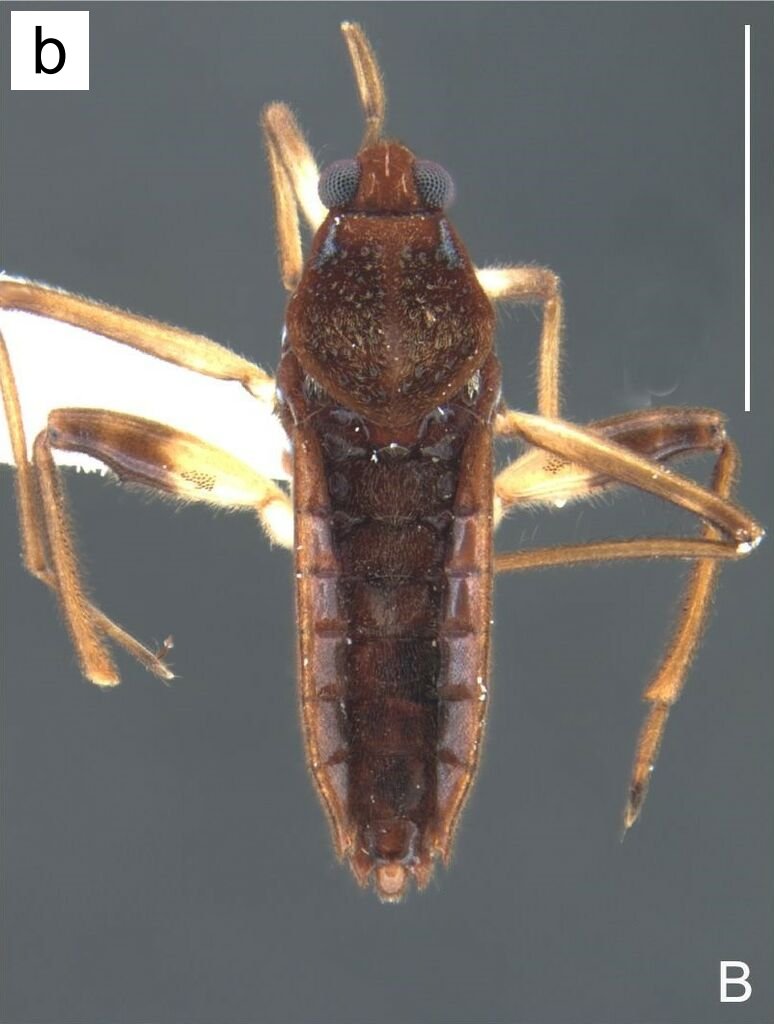
*S.quadrispinosa*, apterous female, habitus, dorsal view

**Figure 26c.**
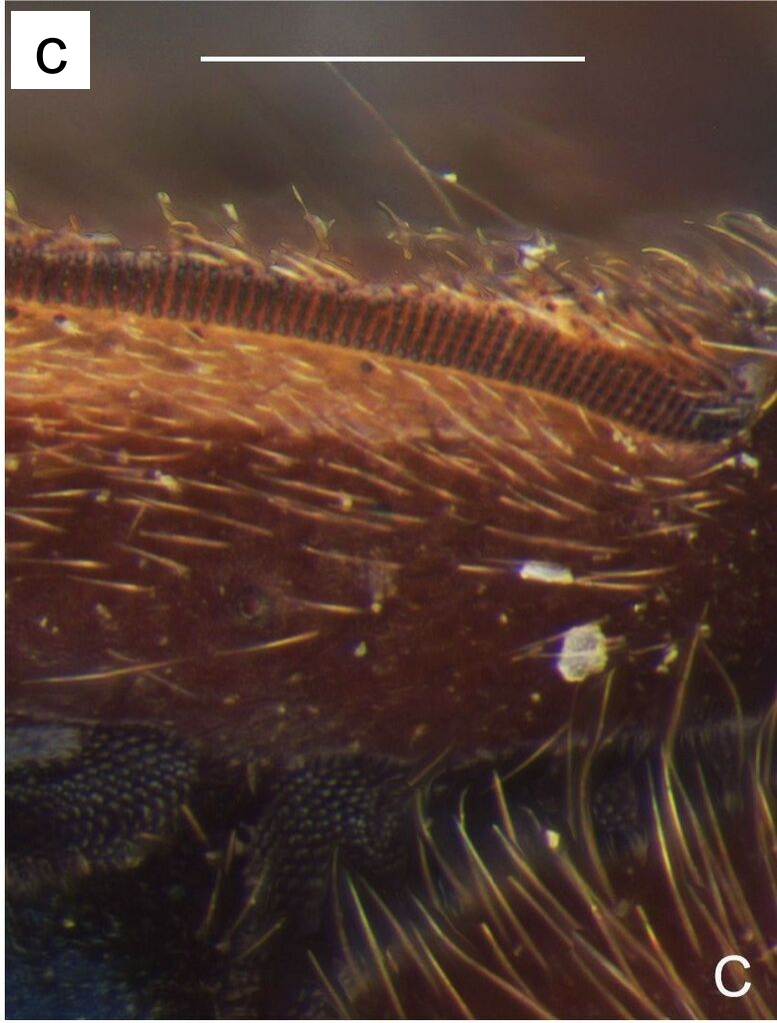
*S.quadrispinosa*, apterous female, detail of stridulatory apparatus, lateral view

**Figure 26d.**
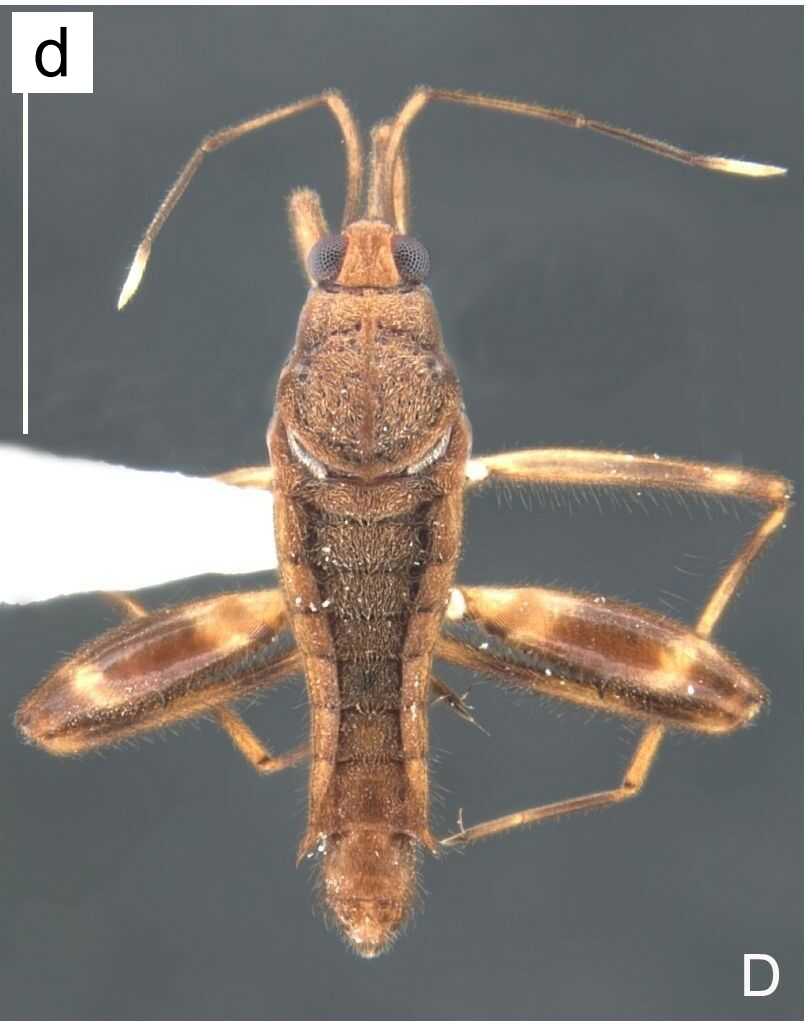
*S.stridulata*, apterous male, habitus, dorsal view

**Figure 26e.**
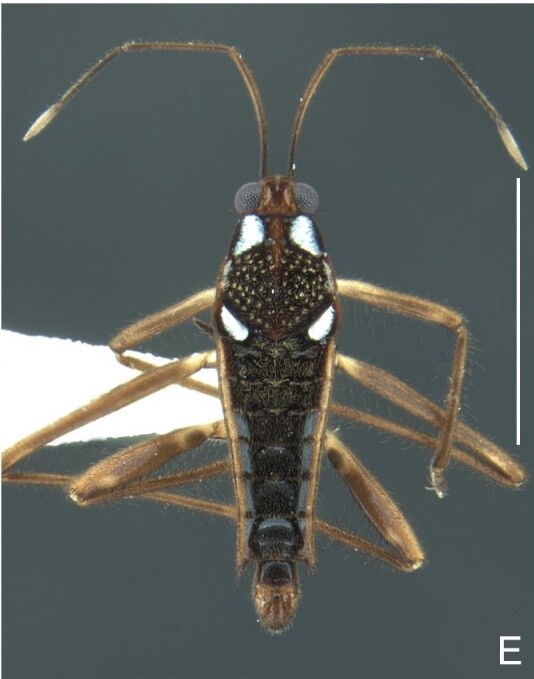
*S.strigosa*, micropterous male, habitus, dorsal view

**Figure 26f.**
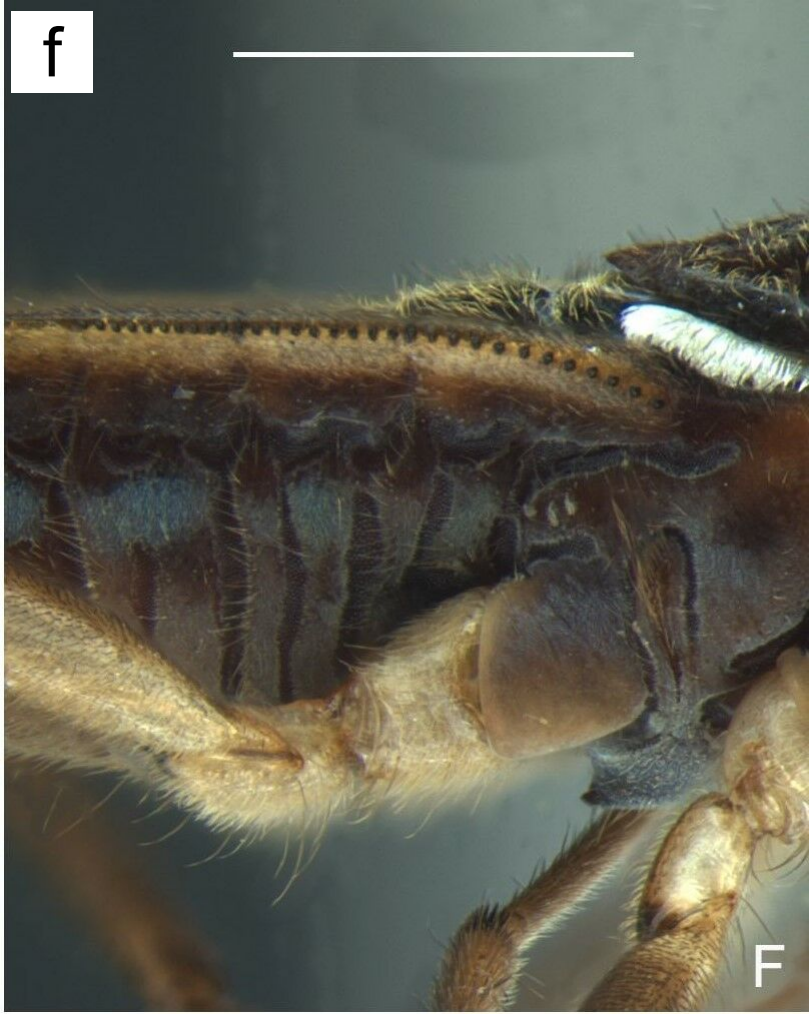
*S.strigosa*, micropterous male, detail of stridulatory apparatus, lateral view

**Figure 27a.**
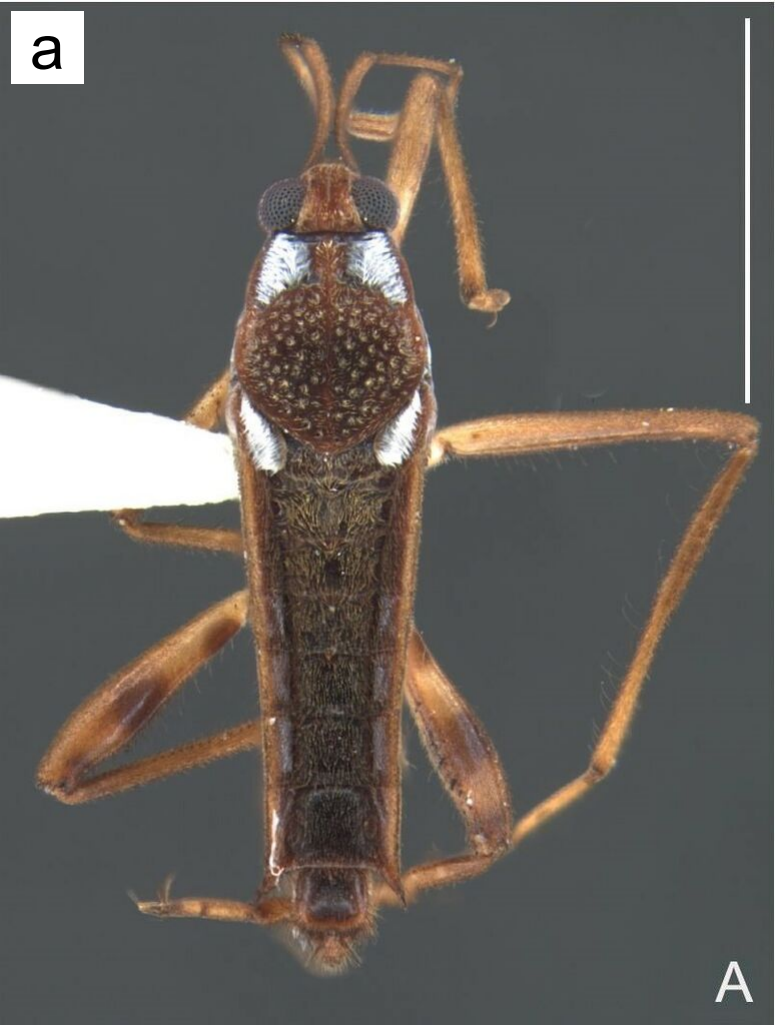
*S.tersa*, micropterous male

**Figure 27b.**
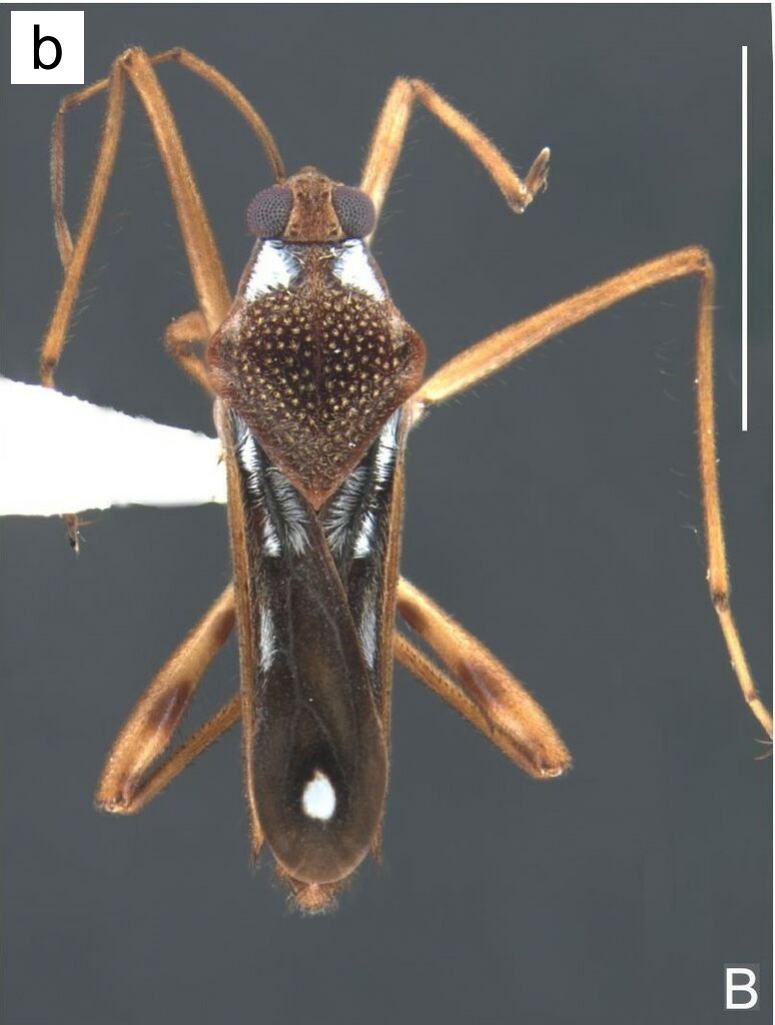
*S.tersa*, macropterous male

**Figure 27c.**
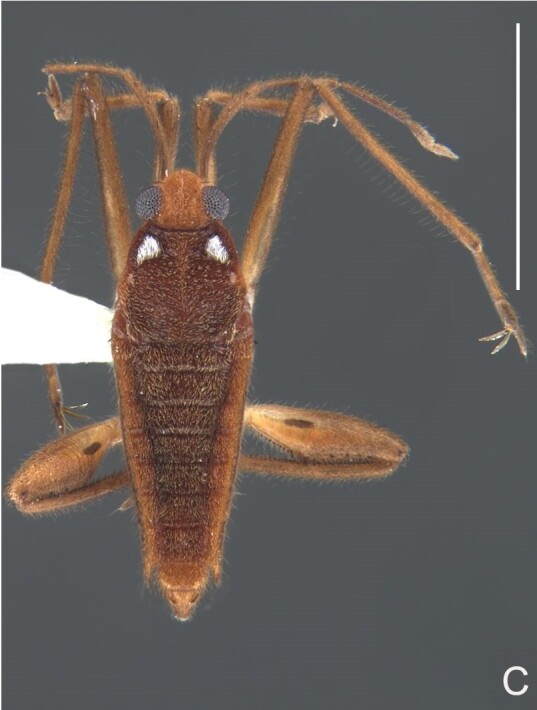
*S.transversa*, apterous male

**Table 1.** Collecting localities of Gerromorpha in aquatic environments of the MRS, Pará, Brazil, from July 2019 to October 2020.

**Municipality**	**Water body**	**Geographic coordinates**	**Abiotic variables**					
			**pH**	**Conductivity (µS/s)**	**Salinity (ppm)**	**OD (ml/l)**	**Turbidity (mtv)**	**Temperature (°C)**
**Belterra**	Igarapé Aramanaí	02°42'56"S; 54°59'59"W	4.61	18.5	0.01	2.4	0.58	26.4
**Belterra**	Igarapé Coronel Batista	02°37'50"S; 54°58'12"W	4.83	12.2	0,00	3.9	1.29	26.6
**Belterra**	Igarapé do Ailton	02°35'36"S; 54°57'48"W	4.81	22.1	0.01	1.3	0.32	28.7
**Belterra**	Igarapé Jatuarana	03°15'44"S; 54°56'37"W	6.53	13.0	0.00	8.9	3.68	26.2
**Belterra**	BR-163, Km-115, igarapé	03°17'34"S; 54°52'45"W	4.25	18.9	-	-	-	26.3
**Belterra**	Floresta Nacional do Tapajós, igarapé	03°03'02"S; 54°55'30"W	4.20	13.3	-	-	-	26.7
**Mojuí dos Campos**	Igarapé Água Fria, nascente	02°47'19"S; 54°38'40"W	6.50	29.1	0.00	2.0	1.61	26.4
**Mojuí dos Campos**	Igarapé Antonio Leite	03°09'06"S; 54°50'28"W	4.40	11.4	-	-	-	26.0
**Mojuí dos Campos**	Igarapé do Manel	02°25'06"S; 54°44'26"W	6.88	10.6	0.00	3.9	14.05	27.9
**Mojuí dos Campos**	Igarapé Mojuí dos Caboclos	02°42'03"S; 54°41'01"W	5.78	13.2	0.00	10.2	3.05	27.5
**Mojuí dos Campos**	Igarapé Santa Júlia	02°40'19"S; 54°43'06"W	5.37	13.4	0.00	4.2	3.17	27.2
**Mojuí dos Campos**	Igarapé Terra de Areia	02°47'58"S; 54°38'15"W	6.58	13.5	0.01	5.3	2.28	29.3
**Mojuí dos Campos**	Igarapé Terra Preta	02°43'09"S; 54°40'20"W	5.25	18.8	0.00	2.9	2.54	30.8
**Santarém**	Cachoeira da Cavada	02°35'48"S; 54°31'47"W	4.77	13.8	0.00	3.3	-	27.2
**Santarém**	Cachoeira da Rocha Negra	02°29'48"S; 54°45'13"W	4.38	11.4	0.00	8.9	2.12	26.6
**Santarém**	Caixa d’água	02°27'31"S; 54°44'49"W	-	-	-	-	-	-
**Santarém**	Igarapé Cajutuba II	02°27'39"S; 54°46'53"W	-	-	-	-	-	-
**Santarém**	Igarapé da Débora, nascente	02°44'27"S; 54°26'01"W	4.5	17.6	-	-	-	26.0
**Santarém**	Igarapé das bananeiras	02°30'52"S; 54°54'20"W	-	-	-	-	-	-
**Santarém**	Igarapé Diamantino	02°30'16"S; 54°39'32"W	4.56	12.6	0.00	-	1.42	27.1
**Santarém**	Igarapé do Rai	02°35'35"S; 54°30'18"W	4.67	12.3	0.00	3.7	1.23	26.7
**Santarém**	Igarapé Guaraná	02°46'25"S; 54°23'20"W	-	-	-	-	-	-
**Santarém**	Igarapé Jatobá	02°34'17"S; 54°51'36"W	-	-	-	-	-	-
**Santarém**	Igarapé Mararú	02°29'35"S; 54°40'06"W	4.92	10.4	0.00	2.1	5.86	28.3
**Santarém**	Igarapé Mutunuy	02°28'53"S; 54°41'45"W	-	-	-	-	-	-
**Santarém**	Ponte do Juá	02°26'40"S; 54°47'21"W	6.17	11.0	0.00	5.5	-	26.0
**Santarém**	Igarapé São Braz	02°29'07"S; 54°49'41"W	5.36	20.7	0.01	-	1.55	26.2
**Santarém**	Igarapé Sonrizal	02°32'13"S; 54°55'26"W	5.8	15.1	0.00	-	2.12	25.6
**Santarém**	Igarapé Urumari	02°28'25"S; 54°41'52"W	-	-	-	-	-	-
**Santarém**	Igarapé Vila Nova	02°30'50"S; 54°49'29"W	-	-	-	-	-	-
**Santarém**	Lago Mapiri	02°25'28"S; 54°44'47"W	6.4	23.2	-	4.4	10.05	30.8
**Santarém**	Puddle	02°27'32"S; 54°44'48"W	-	-	-	-	-	-
**Santarém**	Lago do Juá	02°25'57"S; 54°46'55"W	6.0	9.2	-	4.9	14.13	28.6

**Table 2.** Distribution of semi-aquatic bug species in the three Municipalities of the MRS, Pará, Brazil and references for the records. A single asterisk (*) indicates a new record from Pará State. Two asterisks (**) indicate a new record from Brazil.

**Taxa**	**Belterra**	**Mojuí dos Campos**	**Santarém**	**References**
** GERRIDAE **				
** Charmatometrinae **				
* Brachymetra lata *	**x**	**x**	**x**	This work
* Brachymetra shawi *			**x**	This work
** Cylindrostethinae **				
* Cylindrostethus drakei *			**x**	Nieser 1970
* Cylindrostethus palmaris *	**x**	**x**	**x**	Kuitert 1942, this work
** Gerrinae **				
* Limnogonus aduncus aduncus *			**x**	Nieser 1970, this work
* Limnogonus recurvus *	**x**	**x**	**x**	Kuitert 1942, this work
* Neogerris genticus *	**x**		**x**	Drake and Harris 1930, this work
* Neogerris lotus *			**x**	Drake and Harris 1930
* Neogerris lubricus *	**x**	**x**	**x**	Kuitert 1942, Nieser 1970, this work
* Neogerris visendus *	**x**		**x**	Nieser 1970, this work
* Tachygerris adamsoni *			**x**	This work
** Rhagadotarsinae **				
* Rheumatobates crassifemur esakii *			**x**	Hungerford 1954
* Rheumatobates klagei *			**x**	Hungerford 1954, Nieser 1970
** HYDROMETRIDAE **				
** Hydrometrinae **				
* Hydrometra argentina *	**x**		**x**	Champion 1898, this work
** MESOVELIIDAE **				
** Mesoveliinae **				
* Mesovelia mulsanti *	**x**	**x**	**x**	Neering 1954, this work.
* Mesovelia zeteki *			**x**	Moreira et al. 2008
** VELIIDAE **				
** Microveliinae **				
* Microvelia aschnakiranae *			x**	This work
* Microvelia belterrensis * **sp. nov.**	**x**	**x**		This work
* Microvelia hamadae * **sp. nov.**	**x**			This work
* Microvelia longipes *			x*	This work
* Microvelia mimula *		**x**	**x**	Moreira 2021c, this work
* Microvelia pulchella *		**x**	**x**	This work
* Microvelia sousorum * **sp. nov.**		**x**		This work
* Microvelia summersi *			**x**	Moreira et al. 2011a
* Microvelia venustatis *			**x**	Moreira et al. 2011a, this work
** Rhagoveliinae **				
* Rhagovelia amazonensis *			**x**	Polhemus 1997
* Rhagovelia brunae *	**x**	**x**	**x**	This work
* Rhagovelia elegans *	**x**	**x**	**x**	Bacon 1956, Polhemus 1997, this work
* Rhagovelia evidis *	**x**	**x**	**x**	This work
* Rhagovelia graziae *	x**			This work
* Rhagovelia jubata *	**x**		**x**	This work
* Rhagovelia tenuipes *			**x**	Bacon 1956
* Rhagovelia traili *	**x**	**x**	**x**	Bacon 1948, this work
** Veliinae **				
* Callivelia conata *			**x**	This work
* Oiovelia chenae *			**x**	Rodrigues et al. 2014a
* Oiovelia cunucunumana *		**x**		This work
* Paravelia bullialata *	**x**			This work
* Paravelia dilatata *			x*	This work
* Stridulivelia alia *	**x**		**x**	This work
* Stridulivelia quadrispinosa *		**x**	**x**	Hungerford 1929b, this work
* Stridulivelia stridulata *			**x**	This work
* Stridulivelia strigosa *	**x**	**x**	**x**	This work
* Stridulivelia tersa *	**x**	**x**	**x**	This work
* Striduliveliab transversa *	**x**		**x**	This work
